# Calcium and activity-dependent signaling in the developing cerebral cortex

**DOI:** 10.1242/dev.198853

**Published:** 2022-09-14

**Authors:** Arpana Arjun McKinney, Ralitsa Petrova, Georgia Panagiotakos

**Affiliations:** ^1^ Graduate Program in Developmental and Stem Cell Biology, University of California, San Francisco, CA 94143, USA; ^2^ Eli and Edythe Broad Center of Regeneration Medicine and Stem Cell Research, University of California, San Francisco, CA 94143, USA; ^3^ Department of Biochemistry and Biophysics, University of California, San Francisco, CA 94143, USA; ^4^ Kavli Institute for Fundamental Neuroscience, University of California, San Francisco, CA 94143, USA

**Keywords:** Calcium signaling, Cortical development, Neurodevelopmental disorders

## Abstract

Calcium influx can be stimulated by various intra- and extracellular signals to set coordinated gene expression programs into motion. As such, the precise regulation of intracellular calcium represents a nexus between environmental cues and intrinsic genetic programs. Mounting genetic evidence points to a role for the deregulation of intracellular calcium signaling in neuropsychiatric disorders of developmental origin. These findings have prompted renewed enthusiasm for understanding the roles of calcium during normal and dysfunctional prenatal development. In this Review, we describe the fundamental mechanisms through which calcium is spatiotemporally regulated and directs early neurodevelopmental events. We also discuss unanswered questions about intracellular calcium regulation during the emergence of neurodevelopmental disease, and provide evidence that disruption of cell-specific calcium homeostasis and/or redeployment of developmental calcium signaling mechanisms may contribute to adult neurological disorders. We propose that understanding the normal developmental events that build the nervous system will rely on gaining insights into cell type-specific calcium signaling mechanisms. Such an understanding will enable therapeutic strategies targeting calcium-dependent mechanisms to mitigate disease.

## Teaser

**Summary:** In this Review, we describe calcium-dependent mechanisms regulating early neurodevelopmental events in the cortex and provide evidence for calcium deregulation as a convergence point in the etiology of neurodevelopmental disorders.

## Introduction

Calcium, which lies at the hub of multiple signal transduction pathways, is uniquely situated to transduce dynamic biological inputs into distinct cell behaviors (reviewed by Berridge et al., 2003). Calcium influx in the embryonic brain occurs in response to multiple developmental signals, including electrical activity, and dynamic elevations in cytoplasmic calcium are linked to transcriptional programs that are crucial for development, homeostasis and plasticity (reviewed by Greer and Greenberg, 2008; Lyons and West, 2011). In this Review, we present evidence supporting the idea that spatiotemporally regulated and cell-specific functions of calcium signaling transducers underlie the earliest cellular behaviors that build the cerebral cortex. We focus on calcium entry in developing cortical cells, incorporating lessons gleaned from other developing neural populations. We also detail cell biological outputs influenced by the precise control of cytoplasmic calcium, placing special emphasis on stem/progenitor populations and immature neuroblasts. Moreover, we discuss genetic evidence suggesting that deregulation of intracellular calcium represents a potential node of convergence for neurodevelopmental disorders.

## Mechanisms regulating intracellular calcium signaling

**Fig. 1.**
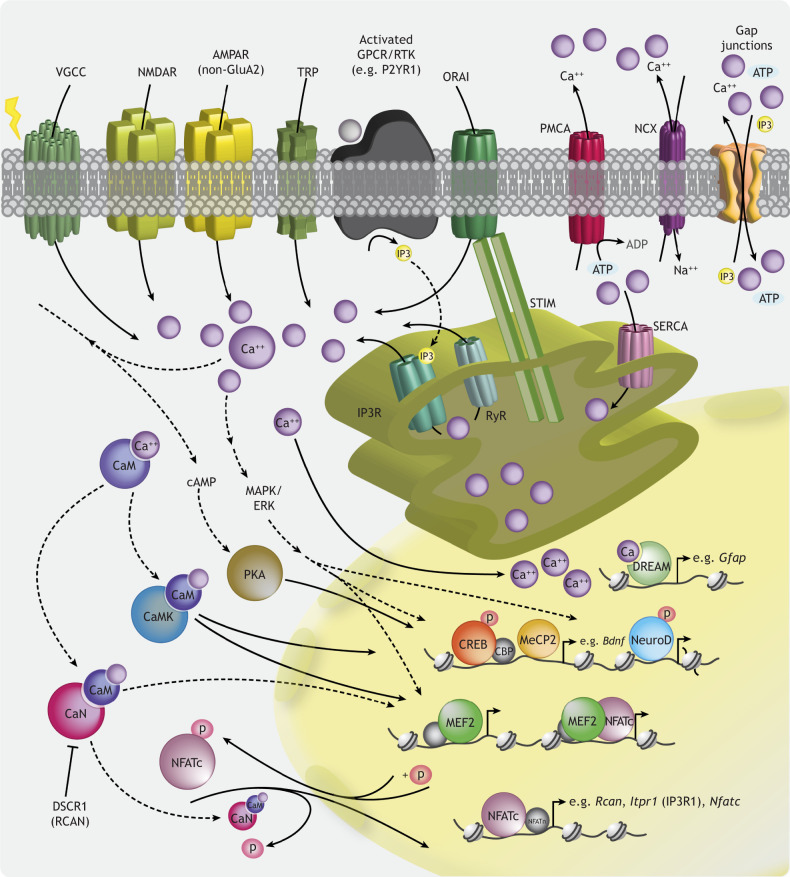
**Overview of intracellular calcium signaling.** Schematic highlighting ion channels and pumps mediating cytoplasmic calcium homeostasis and select calcium-dependent pathways that transduce calcium signals to the nucleus. Low resting cytosolic calcium levels are maintained by plasma membrane calcium ATPases (PMCA1-4, encoded by *ATP2B1*-*4*), which extrude calcium out of the cell and display low calcium efflux capacity but high calcium affinity, and pumps on the surface of the sarco/endoplasmic reticulum (SR/ER) and mitochondria (e.g., the SR/ER calcium ATPases SERCA1-3, encoded by *ATP2A1*-*3*), which transport calcium into intraorganellar stores. Large calcium elevations are countered by sodium calcium exchangers (NCX1-4, encoded by *SLC8A1*-*4*) on the plasma membrane and mitochondrial and ER membranes. Calcium-permeable channels mediating calcium influx from the extracellular space include voltage-gated calcium channels (VGCCs), N-methyl-D-aspartate receptors (NMDARs), α-amino-3-hydroxy-5-methylisoxazole-4-propionic acid receptors (AMPARs), transient receptor potential (TRP) channels and ORAI channels. VGCCs are activated by membrane depolarization, whereas ORAI channels allow calcium influx upon ER calcium store depletion. Successive release of calcium from the ER, mediated by IP3R or RyR, depletes ER calcium, which in turn activates STIM calcium sensors (STIM1 and STIM2) to promote their interaction with ORAI channels (ORAI 1-3) and subsequent calcium influx. Gap junctions in the plasma membrane allow for intercellular propagation of calcium signals, through direct transfer of ions or small molecules that are crucial for intracellular signaling (e.g. IP3). Calcium-sensitive proteins in the cytoplasm (e.g. calmodulin; CaM) undergo a conformational change upon calcium binding, initiating distinct signaling cascades to the nucleus that culminate in calcium-dependent transcription. Note that only select calcium-dependent transcription factors with reported roles in the developing nervous system are highlighted; other factors and organellar calcium stores (e.g. mitochondria, lysosomes, Golgi apparatus) are not depicted.

Calcium signaling involves both the sensing and transduction of extracellular calcium (Brown et al., 1993), as well as the transport of calcium across the plasma membrane (reviewed by Berridge et al., 2003). Homeostatic tuning of intracellular calcium employs an extensive repertoire of cell surface and organellar ion channels, transporters, pumps and buffers (Fig. 1, Box 1), each possessing distinctive properties (e.g. selectivity, conductance, calcium affinity, kinetics). These effectively stage a dynamic process of intracellular compartmentalization, wherein calcium abundance in different subcellular compartments (e.g. cytoplasm, intraorganellar calcium stores) changes across space and time. Movement of calcium into and out of these compartments enables activation of distinct sets of calcium-sensitive proteins, based on their levels and localization (reviewed by Berridge et al., 2003; Clapham, 2007). How distributed networks of calcium signaling proteins confer signaling specificity in various cellular contexts remains a fundamental question. Below, we briefly describe mechanisms of calcium regulation in developing neural cells. We emphasize spatiotemporal properties of calcium signals, alternative splicing of genes regulating calcium signaling, and cell type-specific expression of calcium-dependent effectors. Coupled with the cellular niche and environmental signals to which a cell is exposed, the intersection of these factors influences calcium dynamics, ultimately dictating the selective activation of gene expression programs (reviewed by Clapham, 2007; Rosenberg and Spitzer, 2011).

**Box 1. Key mechanisms of calcium entry in developing neural cells**

An overview of intracellular calcium signaling is depicted in Fig. 1. Here, we focus on channels mediating two types of calcium entry in immature neural cells that are dependent on environmental signals and result in downstream calcium-dependent signaling and transcription.

Voltage-gated calcium channels (VGCCs) are a primary route of calcium influx in developing neural cells (Fig. 1) in response to neural activity. By converting changes in membrane potential at the cell surface into intracellular calcium elevations, VGCCs couple electrical activity to cell biological processes, including phosphorylation and transcription (reviewed by Catterall, 2011; Zamponi et al., 2015). VGCCs consist of a pore-forming subunit, which allows calcium entry in response to depolarizing stimuli, and auxiliary subunits regulating channel localization and function (reviewed by Catterall, 2011; Zamponi et al., 2015). VGCCs are divided into families based on their physiological properties, and different channels display unique properties and localization. This enables distinct roles for VGCCs in different tissues and particular combinations of deficits associated with specific VGCC mutations (reviewed by Catterall, 2011; Zamponi et al., 2015).

Another major source of calcium influx is store operated calcium entry (SOCE), which is activated upon endoplasmic reticulum (ER) calcium store depletion in response to extracellular signals (Fig. 1) (reviewed by Prakriya and Lewis, 2015). SOCE is mediated by ORAI plasma membrane channels (ORAI1-3, also referred to as calcium release-activated calcium, or CRAC, channels) and the stromal interaction molecule (STIM) family of ER calcium sensors (reviewed by Putney et al., 2017). Activation of plasma membrane receptor tyrosine kinases or G-protein coupled receptors by extracellular ligands promotes inositol trisphosphate (IP3)- or ryanodine (Ry)-mediated release of calcium into the cytosol from the ER lumen (reviewed by Prakriya and Lewis, 2015). When intraluminal calcium is depleted, STIM proteins on the ER membrane oligomerize and translocate to ER-plasma membrane junctions, trapping and interacting with ORAI channels to modulate calcium influx (reviewed by Prakriya and Lewis, 2015). SOCE through ORAI channels can generate distinct patterns of intracellular calcium fluctuations to regulate various molecular events, including transcriptional activation (reviewed by Lewis, 2011).

### Spatiotemporal regulation of calcium signals

Calcium influx through different calcium-permeable channels engages distinct signaling cascades (Fig. 1), in part based on the localization of calcium-sensitive signaling proteins (Bading et al., 1993; Graef et al., 1999; reviewed by Parekh, 2008; West et al., 2001). Cytoplasmic calcium chelators, as well as ion pumps and exchangers that bind or extrude calcium (Fig. 1), create transient spatially-restricted high-calcium microenvironments within cells (reviewed by Parekh, 2008). This enables distinct contributions of local and global calcium elevations to intracellular signaling. Functional knock-in experiments and the use of calcium chelators with different affinities have revealed, for example, that local calcium elevations through L-type voltage-gated calcium channels (VGCCs) are transduced to the nucleus via a shuttle protein to promote activation of the transcription factor CREB (Deisseroth et al., 1996; Dolmetsch et al., 2001; Ma et al., 2014). In hippocampal neurons, it has been suggested that the calcium/calmodulin-activated phosphatase, calcineurin (CaN), is tethered to L-type VGCCs via an anchoring protein to contribute to the regulation of calcium influx through these channels (Oliveria et al., 2007, 2012).

Local translation of calcium signaling components can also influence calcium dynamics in specific cellular compartments, such as neuronal dendrites (Sun et al., 2021; reviewed by Holt et al., 2019). Moreover, emerging data reveal that calcium elevations near different intracellular calcium stores (e.g. lysosomes) regulate processes such as autophagy (Medina et al., 2015). In dendrites, endoplasmic reticulum (ER) stores participate in localized calcium signaling that is crucial for synaptic plasticity (Hirabayashi et al., 2017; O'Hare et al., 2022; Takechi et al., 1998). Mitochondrial calcium uptake, regulated by distinct mitochondrial morphologies in different neuronal compartments, can also modulate cytosolic calcium levels and processes such as neurotransmission (Lewis et al., 2018). Notably, spatial regulation of calcium is not restricted to individual cells, as propagation of calcium signals can occur across cohorts of coupled cells in the developing brain (Weissman et al., 2004).

Dynamic patterns of calcium fluctuations also enable signaling specificity, encoding information that is translated into long-lasting biochemical changes by cytosolic proteins with different calcium sensitivities (reviewed by Rosenberg and Spitzer, 2011). In T cells, for example, different patterns of calcium oscillations control transcriptional specificity (reviewed by Dolmetsch et al., 1997, 1998). Seminal studies of the developing *Xenopus* nervous system identified two types of spontaneous calcium transients contributing to distinct aspects of spinal neuron differentiation (Gomez and Spitzer, 1999; Gu and Spitzer, 1995; Gu et al., 1994). Recently, temporal waves of gene expression have been identified in cortical neurons, resulting from activity patterns of different durations (Tyssowski et al., 2018). The initial wave of early-response transcription factors induced by prolonged depolarization, including NPAS4 (Lin et al., 2008), corresponds to activity-dependent programs activated by brief stimulation. Other studies implicate Nuclear Factor of Activated T-cells (NFAT) transcription factors, previously linked to calcium influx through L-type VGCCs (Graef et al., 1999), as somatic calcium spike counters transducing dendritic VGCC activation to the nucleus (Wild et al., 2019). Dissecting how cell type-specific calcium transients encode information in the embryonic cortex will inform how these dynamics drive cell biological changes to impact developmental programs.

### Coordination of routes of calcium entry

VGCCs and store-operated calcium entry (SOCE) are two major sources of calcium influx in developing neural cells (Box 1) (reviewed by Toth et al., 2016). Growing evidence indicates that these parallel entry mechanisms are reciprocally regulated (Park et al., 2010; Wang et al., 2010). In cortical neurons and vascular smooth muscle cells, the ER membrane protein STIM1 (Fig. 1), which activates ORAI channels to promote SOCE, attenuates calcium entry through the L-type VGCC Ca_v_1.2 (Park et al., 2010; Wang et al., 2010). In the reverse direction, changes in membrane potential can alter calcium conductance through ORAI channels (Bakowski and Parekh, 2000). This bidirectional regulation may enable preferential use of store-operated or activity-dependent calcium entry in different cell types. In collaboration with calcium buffers, pumps and transporters, such crosstalk likely tunes calcium dynamics to control intracellular signaling. Much remains to be understood, however, about mechanisms coordinating calcium dynamics in the developing cortex to preferentially activate specific downstream transcriptional pathways.

### Alternative splicing regulates calcium entry and homeostasis

Differentiation in the embryonic cortex is accompanied by global splicing changes (Zhang et al., 2016). Moreover, in different cell and tissue types, alternative splicing of calcium channels and calcium-dependent effectors regulates calcium signaling by generating functionally diverse isoforms (reviewed by Lipscombe et al., 2013). Precise isoform utilization in the embryonic cortex may, therefore, be an important contributor to cell- and region-specific calcium responses to developmental stimuli.

Alternative splicing of VGCC transcripts yields channel variants with specialized roles in different tissues, cells and cellular compartments (reviewed by Abernethy and Soldatov, 2002; Lipscombe et al., 2013). *CACNA1C* (encoding the pore-forming subunit of the VGCC Ca_v_1.2), for example, is extensively spliced to generate channels with distinct properties (Soldatov, 1994; Soldatov et al., 1997; Tang et al., 2004), and long-read sequencing confirms region-specific *CACNA1C* splicing in the adult human brain (Clark et al., 2020). Stereotyped use of specific exons also yields dominant tissue-specific *Cacna1c* isoforms, suggesting the presence of coordinated splicing events (Tang et al., 2007; Welling et al., 1997). Disease-causing mutations restricted to specific isoforms could thus give rise to channelopathies reflecting their expression pattern (reviewed by Abernethy and Soldatov, 2002; Lipscombe et al., 2013).

Calcium channel splicing is also temporally regulated. Our studies and others point to developmental switches in *CACNA1C/Cacna1c* exon use in the brain, implying roles for different channel isoforms over time (Panagiotakos et al., 2019; Tang et al., 2009, 2011). For example, use of two mutually exclusive *CACNA1C* exons (8 and 8a), which are mutated in the syndromic autism spectrum disorder (ASD) Timothy Syndrome (TS), is developmentally controlled (Panagiotakos et al., 2019; Tang et al., 2011). In addition to impeding channel inactivation (Splawski et al., 2004), the TS mutation in exon 8a prevents a normal developmental splicing switch in patient cells (Table S1). This results in continued mutant exon inclusion in developing neurons and ensuing cellular phenotypes contributing to TS (Birey et al., 2017, 2022; Panagiotakos et al., 2019; Paşca et al., 2011; Table S1, discussed in more detail later). How channel subunit isoforms associate with one another, and how their expression is regulated across time, is unclear but likely influences cell type-specific calcium responses.

Tissue- and maturation state-specific PMCA (Fig. 1) isoform expression has also been reported (Brandt and Neve, 1992; Kip et al., 2006). Both neural activity and calcium regulate PMCA splicing (Carafoli et al., 1999; Zacharias and Strehler, 1996), supporting the idea that activity-dependent feedback influences calcium signaling via splicing regulation. Splice variants of many regulators of calcium homeostasis have been identified, including N-methyl-D-aspartate receptors (NMDARs) (An and Grabowski, 2007; Vallano et al., 1999), ORAI1 (Fukushima et al., 2012), STIM1 (Darbellay et al., 2011; Ramesh et al., 2021) and calcium-dependent transcription factors like NFAT (Vihma et al., 2008) and CREB (Walker et al., 1996). Mapping the cell type-specific expression of calcium channel and signaling protein isoforms across development is essential to understanding how calcium elicits specific responses in the embryonic cortex.

## Calcium signaling is deregulated in neurodevelopmental disorders

Mutations in genes impinging on calcium signaling have been implicated in neuropsychiatric disorders of developmental origin. In this section, we discuss genetic evidence highlighting that disrupted calcium-dependent molecular networks may contribute to misregulation of cellular behaviors in the developing brain. To aid our discussion, we surveyed the Simons Foundation Autism Research Initiative (SFARI) Gene database for studies implicating regulators of calcium signaling in the etiology of neurodevelopmental disorders. We restrict our analysis (summarized in Table S1 and discussed below) to genes encoding proteins that either: (1) directly regulate calcium entry, signaling or homeostasis, or (2) indirectly modulate calcium signaling by altering membrane potential or excitability. Due to space constraints, we have not included synaptic structural proteins that regulate ion/calcium channel localization and function at synapses [e.g. ANK2 (Kline et al., 2014), NRXN1-3 (Luo et al., 2020; Missler et al., 2003)], which have been reproducibly implicated in ASD.

Genetic studies have associated mutations in VGCC subunits with increased risk for neuropsychiatric disorders (Table S1). For example, *CACNA1C* variants are associated with bipolar disorder, schizophrenia and ASD (Cross-Disorder Group of the Psychiatric Genomics Consortium, 2013; reviewed by Bhat et al., 2012). Classical TS, a syndromic ASD, is caused by a point mutation in *CACNA1C* (Table S1). This mutation impairs both voltage-dependent Ca_v_1.2 channel inactivation, resulting in elevated depolarization-induced calcium, and channel splicing, leading to persistent mutant channel expression in immature neurons (Panagiotakos et al., 2019; Paşca et al., 2011; Splawski et al., 2004). The ensuing abnormalities in channel signaling yield a constellation of cellular phenotypes, including differentiation deficits, activity-dependent dendritic retraction and impaired interneuron migration (Birey et al., 2017; Krey et al., 2013; Panagiotakos et al., 2019; Paşca et al., 2011). Mutations in genes encoding other calcium-permeable channels, such as GluN2 NMDAR subunits, have also been linked to neurodevelopmental conditions (Endele et al., 2010) (Table S1). In addition, variants of *ATP2B2* (Iossifov et al., 2014), which encodes the PMCA2 calcium pump, and missense mutations in *ITPR1*, which encodes the IP3 receptor type 1 (IP3R1), are implicated in ASD (De Rubeis et al., 2014; Iossifov et al., 2014; Wang et al., 2016). In line with this, fibroblasts from individuals with syndromic and sporadic ASD display attenuated IP3-dependent calcium signaling (Schmunk et al., 2015, 2017).

Mutations in other ion channels and neurotransmitter receptors, which can indirectly influence voltage-dependent calcium influx, are also associated with neurodevelopmental disorders. Genetic variants in *SCN2A*, encoding the Na_v_1.2 sodium channel, for example, are strongly linked to infantile epilepsy, ASD and intellectual disability (ID) (reviewed by Sanders et al., 2018) (Table S1). One such mutation (K1422E) in Na_v_1.2 channels renders them calcium-permeable. *Scn2a^K1442E/+^* cortical neurons display larger action potential-evoked calcium transients compared with wild-type neurons, suggesting that this mutation impacts calcium signaling (Echevarria-Cooper et al., 2022). Similarly, potassium channel mutations that alter excitability (e.g. *KCNQ2* variants) and chromosomal abnormalities in loci containing γ-aminobutyric acid receptor (GABAR) genes (e.g. *15q11-13*) have been associated with neurodevelopmental phenotypes (Table S1) (Cook et al., 1998). Imbalances in GABAergic and glutamatergic signaling are postulated to contribute to the etiology of neurodevelopmental disorders (reviewed by Sohal and Rubenstein, 2019), and a recent study identifying genes with altered expression trajectories in ASD further hints at crucial roles for ion channels and GABAergic neurons in ASD pathophysiology (Berto et al., 2022).

In addition to ion channel mutations, activity-dependent transcriptional regulation, which is dependent on calcium, has been implicated in neurodevelopmental psychiatric disorders (Boulting et al., 2021; Sanchez-Priego et al., 2022). Altered activity-dependent splicing networks have also been reported in ASD cohorts (Gandal et al., 2018; Parikshak et al., 2016; Quesnel-Vallières et al., 2016). Finally, even in examples where known calcium signaling effectors are not mutated, alterations in calcium handling have been observed in cells from individuals with neurodevelopmental conditions. For example, cortical organoids from patients with *22q11.2* deletion syndrome, a highly penetrant cause of neuropsychiatric disease, exhibit calcium signaling deficits (Khan et al., 2020). Transplanted human induced pluripotent stem cell (iPSC)-derived astrocytes from ASD individuals also display elevated calcium responses (Allen et al., 2022). These observations suggest that calcium signaling may be a convergence point for multiple developmental neuropsychiatric disorders.

## Calcium-dependent regulation of cellular behaviors in the developing cortex

Development of the cerebral cortex involves a series of spatiotemporally regulated cellular events, including neural stem and progenitor cell (NSPC) proliferation (reviewed by Libé-Philippot and Vanderhaeghen, 2021; Lin et al., 2021; Llorca and Marín, 2021), migration of newborn neuroblasts into the cortical plate (CP) (reviewed by Francis and Cappello, 2021; Silva et al., 2019) and the differentiation of these cells into mature, synaptically active neurons and glia (reviewed by Bonnefont and Vanderhaeghen, 2021; Taverna et al., 2014) (Box 2). Spontaneous and agonist-induced calcium elevations, neurotransmitter- and depolarization-evoked calcium influx, and SOCE have been observed at various stages of embryonic and adult NSPC lineage progression (reviewed by Toth et al., 2016; Uhlén et al., 2015). These different forms of calcium entry result in the induction of calcium-dependent transcriptional cascades (e.g. CREB-, MEF2-, NFAT- and NPAS4-dependent gene expression) that contribute to developmental regulation of cellular behaviors (reviewed by Greer and Greenberg, 2008). Achieving a granular picture of how cell type-specific calcium signaling impinges on lineage progression in the developing cortex is essential for understanding development and neurodevelopmental disease. Below, we link calcium responses in developing cortical cell populations with the cellular machinery mediating these signals and controlling different cellular behaviors.

**Box 2. Embryonic corticogenesis in rodents and humans**

During early development, neuroepithelial cells give rise to radial glial cells (RGCs), proliferative neural stem cells that populate the cortex with neurons and astrocytes (reviewed by Libé-Philippot and Vanderhaeghen, 2021; Lin et al., 2021). RGCs initially divide symmetrically and subsequently switch to asymmetric divisions to generate postmitotic migratory neuroblasts and intermediate progenitor cells (IPCs; Haubensak et al., 2004; Noctor et al., 2004). Residing in the pseudostratified ventricular zone (VZ) adjacent to the ventricles, RGCs maintain contact with the overlying pia through a long radial fiber. Newborn neuroblasts exit the VZ and migrate along RGC fibers to reach their final laminar position. Newly generated IPCs detach from the ventricular surface and migrate into the subventricular zone (SVZ), where they divide symmetrically to produce daughter neurons. Young neuroblasts sequentially exit the VZ/SVZ to build the cortex in an inside-out fashion, terminally differentiating in the cortical plate (CP) (reviewed by Bonnefont and Vanderhaeghen, 2021). Early-generated subplate (SP) and Cajal-Retzius (CR) neurons, residing beneath the CP and in the marginal zone (MZ), respectively, are central to the development of cortical circuits (reviewed by Hoerder-Suabedissen and Molnár, 2015; Kanold, 2019; López-Bendito, 2018). In particular, the transient SP population plays an indispensable role in guiding thalamocortical axons innervating the developing cortex (reviewed by López-Bendito, 2018). Cortical interneurons are generated in the ventral telencephalon, tangentially migrating from their germinal centers into the cortex (reviewed by Lim et al., 2018; Silva et al., 2019). In humans, an expanded germinal zone overlying the VZ/SVZ (the outer SVZ; oSVZ) harbors outer radial glia (oRG), which generate cortical neurons and are thought to contribute to the evolutionary expansion of the human neocortex (Fietz et al., 2010; Hansen et al., 2010). Not depicted are less abundant cell types with important roles in cortical development, including microglia, endothelial cells and pericytes. iSVZ, inner SVZ; IZ, intermediate zone.

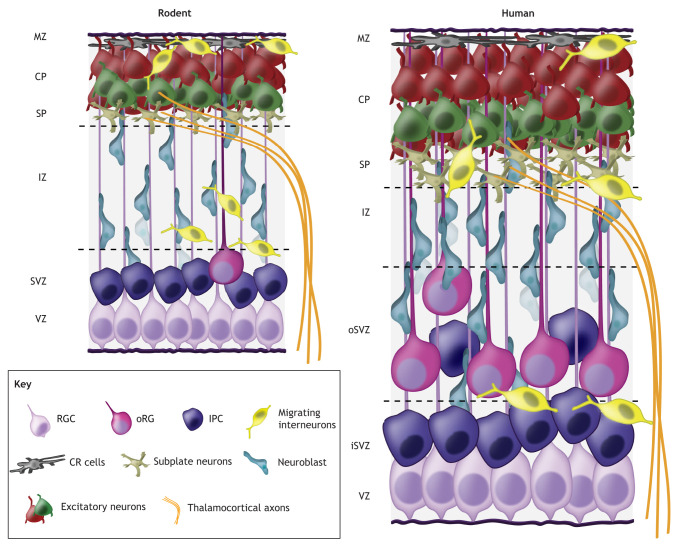

### Cortical NSPC proliferation

#### Spontaneous calcium elevations

**Fig. 2.**
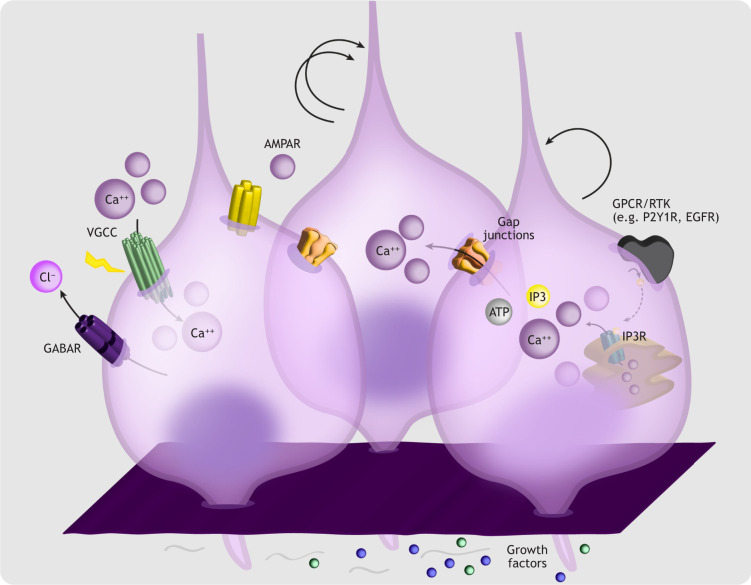
**Calcium-dependent regulation of proliferation during corticogenesis.** Calcium imaging and electrophysiological recordings performed on embryonic rodent cortical slice cultures demonstrate that radial glial cells (RGCs) in the ventricular zone (VZ) exhibit spontaneous and induced calcium rises. Purinergic signaling through metabotropic P2Y1 receptors initiates calcium transients that propagate across VZ cells. These calcium waves, which modulate proliferation, require gap junctions and IP3-mediated calcium release. RGC primary cilia protrude into the ventricles, where they are exposed to diffusible growth factors in the CSF that initiate calcium rises and also influence RGC division. Finally, depolarization mediated by GABA and glutamate acting on GABARs and AMPARs, respectively, controls proliferation by inducing calcium rises through VGCCs.

The proliferative NSPC compartment comprises radial glial cells (RGCs) and intermediate progenitor cells (IPCs) (Box 2), which give rise to cortical excitatory neurons and astrocytes. Residing adjacent to the ventricles, these cells are exposed to environmental stimuli impinging on calcium signaling during development, including growth factors and electrical activity (reviewed by Dehay and Kennedy, 2007; Fame and Lehtinen, 2020). Cortical NSPCs express ion channels, pumps and receptors that generate unique calcium dynamics to contribute to developmental cellular behaviors (Fig. 2). Different patterns of spontaneous calcium elevations are observed in the ventricular zone (VZ): slow rises are contained to individual cells, whereas coordinated transients (calcium waves) propagate across gap junction-coupled, mitotically-active RGCs (Bittman et al., 1997; Lo Turco and Kriegstein, 1991; Owens et al., 2000; Weissman et al., 2004).

Spontaneous calcium rises are dependent on internal calcium stores, as they persist in the absence of extracellular calcium but are eliminated upon ER calcium depletion (Owens and Kriegstein, 1998). VGCC activation, neurotransmitter signaling and depolarization are not necessary to promote spontaneous rises (Owens and Kriegstein, 1998; Weissman et al., 2004). Instead, initiation of these calcium transients requires purinergic signaling via metabotropic P2Y1 ATP receptors (P2Y1Rs) (Liu et al., 2008; Malmersjö et al., 2013; Owens and Kriegstein, 1998; Owens et al., 2000; Weissman et al., 2004), and NSPCs have been identified as a source of ATP eliciting pro-proliferative calcium responses (Lin et al., 2007). Calcium waves are activated by extracellular ATP in a temporally regulated fashion, occurring robustly at the peak of neurogenesis and propagating across dynamically coupled RGCs via connexin hemichannels at specific cell cycle stages (Bittman et al., 1997; Owens and Kriegstein, 1998; Weissman et al., 2004). Abrogating these waves by antagonizing P2Y1Rs or inhibiting gap junctions significantly reduces proliferation and promotes differentiation (Lin et al., 2007; Malmersjö et al., 2013; Weissman et al., 2004). Single cell RNA-sequencing (scRNA-seq) and calcium imaging studies demonstrate that P2Y1Rs are highly expressed in ventricular RGCs and IPCs of the rodent and human fetal cortex, and that P2Y1R agonists induce calcium rises in these cells (Mayer et al., 2019). P2Y1Rs are downregulated in neurons and, intriguingly, in human outer radial glia (oRG), a neural stem cell population abundant in humans that may contribute to evolutionary expansion of the neocortex, pointing to a conserved role for ATP-dependent calcium signaling in ventricular RGCs and IPCs (Liu et al., 2008; Mayer et al., 2019).

#### Links between calcium and growth factor signaling

Growth factors are important cell cycle regulators (reviewed by Dehay and Kennedy, 2007) that can induce calcium elevations in proliferative NSPCs. High concentrations of basic fibroblast growth factor (bFGF), for example, elicit robust cytoplasmic calcium rises in the apical end foot and cell body of RGCs, propagating in a sustained manner through the RGC fiber (Rash et al., 2016). bFGF can promote proliferation in concert with epidermal growth factor (EGF) (Tropepe et al., 1999), which stimulates ER calcium store depletion to activate SOCE and induce NFAT-dependent transcription in ganglionic eminence (GE)-derived ventral progenitors and adult subventricular zone (SVZ) cells (Somasundaram et al., 2014). NFATs have been linked to cell cycle regulation (Carvalho et al., 2007; Teixeira et al., 2016) and progenitor proliferation in the neural tube and postnatal neural cultures (Huang et al., 2011; Serrano-Pérez et al., 2015). In GE progenitors, inhibiting SOCE using genetic and pharmacological approaches decreases proliferation (Somasundaram et al., 2014). Although the function and upstream activators of SOCE in cortical NSPCs remain unknown, spontaneous calcium waves in the VZ are partially mediated by intracellular calcium stores and IP3-dependent calcium release (Weissman et al., 2004). Application of the IP3 receptor antagonist 2-aminoethoxydiphenyl borate, at doses that inhibit SOCE, reduces the amplitude and duration of calcium transients in cortical RGCs (Rash et al., 2016), pointing to potential roles for SOCE in proliferative NSPCs.

#### Neurotransmitter signaling and calcium

Neurotransmitter signaling also influences cortical NSPC calcium elevations to regulate cell division. In the embryonic brain, upon binding to GABA_A_Rs, the neurotransmitter GABA depolarizes immature cells (reviewed by Ben-Ari et al., 2007). GABA_A_R-dependent depolarization results from unopposed developmental activity of the Na^+^-K^+^-2Cl^−^ transporter NKCC1 (also known as SLC12A2), which yields elevated chloride concentrations in embryonic neural cells (Owens et al., 1996, 1999; reviewed by Ben-Ari et al., 2007). The postnatal emergence of the K^+^-Cl^−^ co-transporter KCC2 (SLC12A5) induces a developmental switch in GABA activity from depolarizing to hyperpolarizing (reviewed by Ben-Ari et al., 2007). This maturation of inhibition is regulated by the activity of the largely transient subplate (SP) neuron population (Kanold and Shatz, 2006), by GABA (Ganguly et al., 2001) and by growth factor signaling (Rivera et al., 2004). The role of neural activity in developmental regulation of intracellular chloride and the transition to GABA inhibition is supported by studies demonstrating that manipulating excitatory input or sensory experience modulates KCC2 expression (He et al., 2010; Kanold and Shatz, 2006; Sernagor et al., 2003). Activity and cytosolic calcium also alter chloride gradients in immature hippocampal neurons (Fiumelli et al., 2005), whereas activity-dependent neurotrophins modulate KCC2 expression and GABAergic inhibition (Aguado et al., 2003; Ludwig et al., 2011).

Embryonic cortical NSPCs express functional GABA_A_ receptors (Lo Turco et al., 1995; Mayer et al., 2019; Owens et al., 1996) and GABA-induced depolarization elicits calcium transients in NSPCs through VGCC activation (Lo Turco et al., 1995; Mayer et al., 2019; Owens et al., 1996, 1999; Panagiotakos et al., 2019). GABA depolarization inhibits DNA synthesis, decreasing cortical NSPC proliferation (Antonopoulos et al., 1997; Lo Turco et al., 1995). This effect can be rescued by application of chloride transport blockers, suggesting that the depolarizing activity of GABA underlies its ability to suppress NSPC proliferation (Lo Turco et al., 1995). Importantly, separately dissecting the effects of GABA in the VZ and SVZ reveals that GABA inhibits SVZ progenitor proliferation but has pro-proliferative effects in the VZ, shortening the cell cycle to promote mitotic re-entry (Haydar et al., 2000). This suggests that GABA exerts cell type-specific effects on RGCs and IPCs, although how they are transduced to influence calcium in each cell type remains unknown. Understanding how developmental GABAergic activity is linked to the electrical properties of progenitor types, which can change across developmental time (Vitali et al., 2018), represents an exciting avenue for future studies.

Likewise, the excitatory neurotransmitter glutamate depolarizes NSPCs, promoting calcium rises that control proliferation. Although NMDARs are expressed in the developing brain (Henson et al., 2008; Lo Turco et al., 1991; Monyer et al., 1994), a confluence of data points to NMDARs playing an outsized role in post-mitotic neurons compared with NSPCs (Behar et al., 1999; Lo Turco et al., 1991, 1995; Maric et al., 2000; Mayer et al., 2019). In the rat VZ, NMDA does not elicit currents in NSPCs, whereas in human RGCs and oRG, NMDA elicits magnesium-insensitive currents at resting membrane potentials (Lo Turco et al., 1991, 1995; Maric et al., 2000; Mayer et al., 2019). Calcium imaging of human fetal cells identified a small fraction of VZ cells responding to NMDA, but scRNA-seq reveals that these cells are predominantly excitatory neurons and mostly absent from NSPC clusters (Mayer et al., 2019).

In contrast, ionotropic α-amino-3-hydroxy-5-methylisoxazole-4-propionic acid (AMPA) and kainate (KA) receptors are expressed in VZ/SVZ cells (Haydar et al., 2000; Lo Turco et al., 1995; Maric et al., 2000). Four main subunits, GluA1-4, compose heteromeric AMPA receptors (AMPARs) (reviewed by Traynelis et al., 2010), and the presence of GluA2 renders AMPARs calcium impermeable (Sommer et al., 1991). Intriguingly, GluA2 abundance increases postnatally, suggesting that embryonic neural cells are more likely to express calcium-permeable AMPARs (Kumar et al., 2002; reviewed by Behm and Öhman, 2016). In line with this, AMPA induces calcium elevations in isolated NSPCs completing their final division (Maric et al., 2000), and AMPA/KA antagonists block calcium elevations elicited by glutamate in rat cortical VZ cells (Lo Turco et al., 1995). AMPAR stimulation in isolated human fetal cortical NSPCs also promotes differentiation (Mayer et al., 2019; Whitney et al., 2008). Consistent with these findings, glutamate and KA (but not NMDA) significantly inhibit DNA synthesis in VZ cells (Lo Turco et al., 1995). Once again, separating RGCs from IPCs reveals a more complex picture: glutamate increases proliferation in the VZ and decreases SVZ proliferation, highlighting differential responses of distinct progenitor populations to neurotransmitter signaling (Haydar et al., 2000). It will be important to dissect these responses across development, particularly in light of data demonstrating progressive RGC hyperpolarization during cortical neurogenesis as a key regulator of RGC output (Vitali et al., 2018). Notably, among the channels upregulated to promote this developmental change in RGC membrane potential are several calcium-activated K^+^ channels (Vitali et al., 2018). Although dynamic changes in K^+^ channel expression and membrane potential have been linked to the control of proliferation (reviewed by Blackiston et al., 2009), how such changes influence NSPC calcium signaling remains unclear.

The neurotransmitters serotonin (5-HT) and acetylcholine (Ach) also induce NSPC calcium transients. Maternally-derived 5-HT is reported to regulate proliferation in the developing brain (Côté et al., 2007); however, stimulation of the 5-HT receptors HTR2A and HTR2C promotes rat cortical progenitor survival without affecting proliferation (Dooley et al., 1997). HTR2A is highly expressed in human cortical germinal zones, and the HTR2A agonist TCB-2 induces robust calcium rises in human NSPCs (Mayer et al., 2019). HTR2A inhibition alters fiber length in proliferating human RGCs but does not impact division, positing a human-specific role for serotonergic signaling in maintaining RGC structural integrity during proliferation (Mayer et al., 2019). In contrast, muscarine and Ach stimulate calcium influx in cortical NSPCs via muscarinic Ach receptor (mAchR) activation, and mAchR antagonists and calcium chelators attenuate NSPC proliferation (Atluri et al., 2001; Ma et al., 2000). In GE-derived progenitors, Ach, like EGF, stimulates SOCE via mAchRs, and abrogating SOCE reduces proliferation, pointing to potential links between cholinergic activity, SOCE and NSPC divisions (Somasundaram et al., 2014).

It is possible that agonist-induced SOCE and depolarization-induced calcium entry represent mechanisms that antagonistically regulate NSPC proliferation. Understanding the coordination of different modes of calcium entry and how their interplay impacts proliferative NSPCs is thus essential. Moreover, as calcium influx via VGCCs and SOCE can contribute to calcium oscillations, it will be crucial to determine how cellular responses are encoded in patterns of calcium transients and how these dynamics enable signaling specificity in NSPCs.

### Calcium-dependent cellular motility

#### NSPC motility

Proliferative RGCs undergo interkinetic nuclear migration (INM) – dynamic somatic movements in phase with the cell cycle (reviewed by Taverna et al., 2014). During G1, the somata of RGCs move away from the ventricle to complete S-phase, whereas they move apically during G2, initiating mitosis at the ventricular wall. Pharmacological inhibition of connexin hemichannels suppresses RGC calcium waves (Liu et al., 2010; Weissman et al., 2004) and significantly attenuates INM (Liu et al., 2010). Chelating intracellular calcium also reduces INM distance and speed (Liu et al., 2010), suggesting that calcium propagating through coupled RGCs plays a role in the dynamic changes associated with RGC motility.

IPCs do not exhibit INM, instead delaminating from the VZ and moving into the SVZ along RGC fibers (Noctor et al., 2004; reviewed by Taverna et al., 2014). Pharmacological inhibition of purinergic signaling or P2Y receptor knockdown reduces calcium transient frequency in proliferative IPCs, preventing their migration into the SVZ (Liu et al., 2008). Thus, ATP-mediated calcium signaling is not only necessary for NSPC proliferation, but also for the dynamic progenitor movements that shape the developing cortical cytoarchitecture (Liu et al., 2008).

#### Spontaneous calcium rises in postmitotic migratory cells

**Fig. 3.**
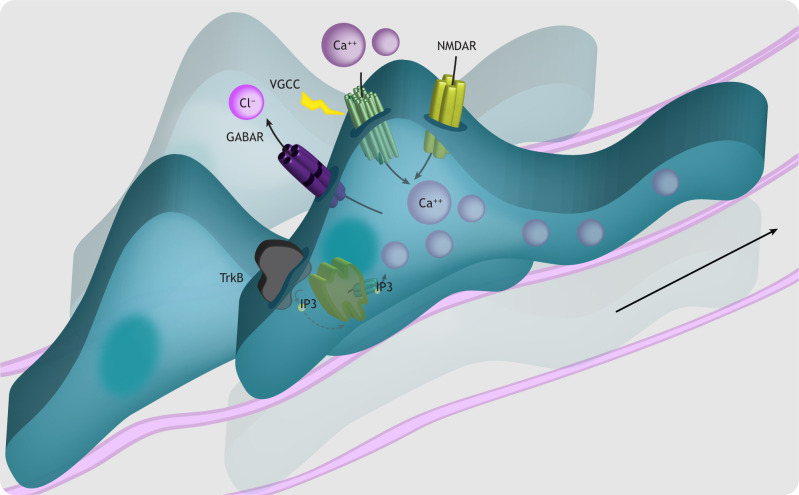
**Calcium and excitatory neuroblast migration.** Excitatory neuroblasts undergo radial migration along RGC fibers to reach their final laminar destination. Migratory neuroblasts exiting the VZ display high frequency spontaneous calcium transients. As they enter the SVZ, neuroblasts adopt a multipolar morphology and exhibit low amplitude calcium events. Calcium transients during neuroblast migration are largely mediated by extracellular agonists like BDNF, which activates TrkB, by neurotransmitter receptors (e.g. NMDARs, GABARs), and by downstream activation of VGCCs. Glutamate influences radial migration by inducing calcium influx primarily through NMDARs. Depolarization via GABARs and calcium influx via VGCCs also control radial migration. Emerging evidence suggests that calcium transients propagating through RGC fibers may also contribute to the regulation of neuroblast migration into the cortical plate.

Spontaneous calcium rises have been linked to the radial migration of excitatory neuroblasts (Box 2; Fig. 3). Newborn neuroblasts exiting the VZ exhibit the highest frequency of somatic bursting calcium transients in the developing cortex (Rash et al., 2016). It has also been postulated that calcium transients in RGC fibers, which act as a scaffold for radial migration (Box 2), signal to neuroblasts to influence their calcium dynamics and migratory behavior (Rash et al., 2016). It remains unclear, however, how calcium dynamics in RGCs and neuroblasts intersect, and what the functional contribution of bursting transients in migratory neuroblasts is to radial migration.

Unlike excitatory neurons, inhibitory cortical interneurons are generated in subpallial structures, including the medial and caudal ganglionic eminences (MGE and CGE, respectively), undertaking saltatory tangential migration to reach the cortex (reviewed by Buchsbaum and Cappello, 2019; Contractor et al., 2021; Lim et al., 2018; Silva et al., 2019). Within the cortex, interneurons undergo both tangential and radial migration to their appropriate laminar destination (Box 2) (Tanaka et al., 2006; reviewed by Silva et al., 2019). Migratory interneurons exhibit spontaneous calcium transients characterized by oscillatory bursts or individual spikes (Bortone and Polleux, 2009; Martini and Valdeolmillos, 2010). Chelating intracellular calcium or pharmacologically blocking VGCC activity significantly disrupts cytoskeletal dynamics and impairs interneuron migration (Bortone and Polleux, 2009; Martini and Valdeolmillos, 2010). Caffeine-induced ER calcium release also promotes cytoskeletal changes to stimulate interneuron motility (Martini and Valdeolmillos, 2010). Interestingly, SOCE is attenuated in migratory neuroblasts isolated from the GE (Somasundaram et al., 2014), again raising the question of how different modes of calcium entry intersect in developing neurons to coordinate migration.

#### Agonist-induced calcium rises in migratory neuroblasts

Tyrosine receptor kinase B (TrkB; Ntrk2), canonically bound by brain derived neurotrophic factor (BDNF), has been linked to embryonic cortical neuroblast migration (Bartkowska et al., 2007; Behar et al., 1997) (Fig. 3). BDNF stimulates calcium transients and increases cortical neuroblast migration, which can be blocked by application of Trk inhibitors or calcium chelators (Behar et al., 1997). This suggests that TrkB activation promotes migration partly by inducing downstream calcium signaling, although it should be noted that EGF robustly transactivates TrkB *in vivo* to stimulate cortical neuroblast migration (Puehringer et al., 2013).

TrkB is also involved in tangential interneuron migration (reviewed by Contractor et al., 2021; Silva et al., 2019). BDNF and neurotrophin 4 (NTF4) activate interneuron TrkB receptors, and Trk inhibitor application reduces the fraction of MGE-derived cells migrating into the cortex. In mice lacking *Trkb*, the number of calbindin-positive interneurons that reach the cortex is attenuated (Polleux et al., 2002). In contrast to TrkB-mediated activation of PLCγ and MAP kinase in excitatory neuroblasts, BDNF activates PI3-kinase signaling in interneurons to control tangential migration (Polleux et al., 2002). Intriguingly, BDNF- and NTF4-dependent TrkB activation has been implicated in KCC2 regulation, positing a link between growth factor signaling and excitability in developing neurons (Rivera et al., 2004).

#### Neurotransmitter-induced calcium signaling in migrating neuroblasts

Intracellular calcium modulation via GABAR-mediated signaling also impacts radially migrating cortical neurons. In excitatory neuroblasts, GABA induces calcium transients and stimulates migration (Fig. 3) (Behar et al., 1996). BAPTA blocks these effects, suggesting that calcium transduces GABA stimulation into migratory behavior (Behar et al., 1996). Indeed, GABA_A_ and GABA_B_ receptors are functionally expressed in migratory cortical neurons (López-Bendito et al., 2002; Owens et al., 1996, 1999). Pharmacological studies in embryonic rat cortical slices indicate that activation of different GABARs plays distinct region-specific roles across the cerebral wall (Behar et al., 2000). Similar observations have been replicated *in vivo*, where local application of GABA_A_R antagonists or chronic administration of desensitizing levels of GABA_A_R agonists attenuate spontaneous calcium transients, resulting in cortical heterotopias (Heck et al., 2007). Notably, although GABA has been reproducibly implicated in migration, mice lacking *Gad65* (*Gad2*) and *Gad67* (*Gad1*) do not display major cortical malformations (Ji et al., 1999), and radial migration is not significantly altered in *Gad67* knockout mice (Furukawa et al., 2014). Other endogenous activators of GABA_A_Rs (e.g. taurine) have thus been proposed to control neuronal migration (Furukawa et al., 2014).

Tangentially migrating cortical interneurons also display GABAR-mediated calcium transients (Soria and Valdeolmillos, 2002). As immature interneurons migrate from the MGE, GABA exerts a depolarizing effect via GABA_A_Rs (Bortone and Polleux, 2009; Cuzon et al., 2006), and antagonizing GABA_A_Rs results in accumulation of migrating interneurons at the pallial-subpallial boundary (Cuzon et al., 2006). GABA depolarization activates VGCCs to promote calcium influx, acting as a pro-migratory signal, presumably via calcium-dependent pathways to the cytoskeleton (Bortone and Polleux, 2009). During the first postnatal week in mice, KCC2 upregulation renders GABA hyperpolarizing, leading to decreased calcium transients and reduced interneuron motility to terminate migration (Bortone and Polleux, 2009). Suppressing the activity of CGE-derived interneurons at distinct developmental stages does not affect tangential migration but reveals essential contributions of activity-dependent signaling in the radial migration of specific subpopulations into their final laminar positions in the CP (De Marco García et al., 2011).

Glycine receptor (GlyR) activation by endogenous ligands (e.g. glycine, taurine) is also depolarizing in cortical neuroblasts, eliciting calcium rises that may modulate migration (Avila et al., 2013; Flint et al., 1998; Yoshida et al., 2004). Although cortices from mice lacking the developmentally enriched α2 GlyR display no gross morphological defects (Young-Pearse et al., 2006), genetic deletion of these receptors results in impaired interneuron migration (Avila et al., 2013). In migratory cortical interneurons, extrasynaptic glycine acting on α2 GlyRs stimulates dynamic calcium fluctuations via VGCCs, promoting motility through calcium-dependent tuning of actomyosin contractions (Avila et al., 2013). In excitatory neuroblasts, the role of GlyRs is less clear (Furukawa et al., 2014), although GlyR activation in organotypic slice cultures, in the presence of glycine uptake inhibitors, impedes radial migration (Nimmervoll et al., 2011).

Seminal experiments in cerebellar granule cells first demonstrated calcium-dependent roles for glutamate in neuroblast migration (Komuro and Rakic, 1993). In the developing cerebral cortex, glutamate released by post-mitotic neurons induces calcium transients in radially migrating neuroblasts, modulating their motility primarily through NMDARs (Fig. 3) (Behar et al., 1999; Hirai et al., 1999). NMDARs are heteromers, the majority consisting of two GluN1 and two GluN2 subunits. Whereas GluN1 subunits are expressed before and after birth in the cortex, GluN2 subunit expression is dynamically regulated during development, resulting in NMDARs with markedly different physiological properties. Compared with those containing GluN2B subunits, GluN2A-containing NMDARs have faster deactivation kinetics, higher open probabilities and lower sensitivity to agonists. Although both NMDAR subtypes display similar calcium permeability, their unique gating properties shape the dynamics of their contribution to calcium influx to influence downstream signaling (Erreger et al., 2005; Sheng et al., 1994; reviewed by Paoletti et al., 2013; Wyllie et al., 2013). In humans, *GRIN2B*, encoding GluN2B, is highly expressed in postmitotic embryonic neurons, whereas *GRIN2A*, encoding GluN2A, is expressed in embryonic RGCs and neurons after birth (Mayer et al., 2019). This developmental subunit switch, which occurs in early postnatal life in the rodent and can be regulated by neural activity (reviewed by Yashiro and Philpot, 2008), results in enriched GluN2B abundance during the peak of neuroblast migration. Consequently, shRNA-dependent GluN2B and GluN1 knockdown in embryonic rodent cortices delays neuronal migration, whereas manipulating GluN2A does not affect neuroblast motility (Jiang et al., 2015). Pharmacological NMDAR inhibition or calcium chelators in the presence of NMDA also abrogate neuroblast migration (Behar et al., 1999; Hirai et al., 1999; Reiprich et al., 2005; Yuryev et al., 2018). More recently, transient glutamatergic transmission from SP neurons onto excitatory neuroblasts was shown to regulate neuroblast migration in an NMDAR-dependent manner (Ohtaka-Maruyama et al., 2018). Although these studies suggest that NMDARs are essential for radial migration, genetic inactivation of *Grin1*, encoding GluN1, reveals no major deficits in neuronal distribution (Messersmith et al., 1997; Iwasato et al., 2000). This may result from mechanisms compensating for long-term GluN1 loss of function (reviewed by Luhmann et al., 2015; Medvedeva and Pierani, 2020). In tangentially migrating cortical interneurons, NMDA and Kainate also induce calcium transients (Soria and Valdeolmillos, 2002), and activation of NMDARs or AMPARs positively regulates interneuron motility in a VGCC-dependent manner (Bortone and Polleux, 2009). Within the intermediate zone (IZ), tangentially migrating interneurons continue to express calcium-permeable AMPARs (Métin et al., 2000) and, in CGE-derived interneuron subtypes, 5-HT3A receptor activation induces calcium transients required for proper migration (Murthy et al., 2014).

#### VGCCs in neuroblast migration

A function for VGCCs in neuronal migration was initially described in cerebellar granule cells (Komuro and Rakic, 1992). In the embryonic cortex, L-type VGCCs are highly expressed in cortical neurons, and migrating upper layer neurons exhibit spontaneous L-type VGCC-mediated calcium transients (Kamijo et al., 2018). *In utero* Ca_v_1.2 overexpression in excitatory neurons destined for upper cortical layers impairs radial migration, and a severe calcium influx-dependent migratory defect in this population results from electroporation of Ca_v_1.2 channels bearing the TS mutation (Kamijo et al., 2018).

L-type VGCCs, and to a lesser extent N-type VGCCs, are also essential for interneuron motility (Bortone and Polleux, 2009). Depolarization of immature interneurons by GABA or glutamate activates VGCCs to promote tangential migration into the cortex (Bortone and Polleux, 2009). Assembloid models using human iPSCs further support a role for Ca_v_1.2 in human cortical interneuron migration, demonstrating that inhibitory neurons from TS patients display abnormal migratory behaviors (Birey et al., 2017). Dissecting these migratory phenotypes demonstrates that VGCC-dependent calcium signaling impinges on distinct molecular networks to influence cellular motility (Birey et al., 2022).

### Calcium and programmed cell death

Developing cortical circuits are refined by precisely regulated apoptosis, which scales down NSPC, pyramidal cell and cortical interneuron populations. Approximately 12% of cortical pyramidal cells undergo apoptosis in the early postnatal period in rodents (Wong et al., 2018), and a substantial fraction of cortical interneurons also undergo postnatal cell death (Priya et al., 2018; Southwell et al., 2012). In both developing populations, a central regulator of apoptosis is neuronal activity and intracellular calcium signaling.

*In vitro* studies in the 1990s first implicated activity-dependent calcium signaling in cortical pyramidal neuron survival (Ghosh et al., 1994; Voigt et al., 1997). Stimulation of embryonic cortical cultures with potassium chloride (KCl) enhances cell survival by promoting neurotrophin expression, an effect that can be eliminated by chelating calcium or pharmacologically blocking L-type VGCCs (Ghosh et al., 1994). NMDARs contribute to developmental apoptosis of pyramidal cells during discrete temporal windows, as early postnatal NMDAR inhibition promotes cell death. This effect can be rescued by concurrent VGCC activation, suggesting that VGCCs and NMDARs have overlapping roles in promoting calcium-dependent survival (Ghosh et al., 1994; Heck et al., 2008; Ikonomidou et al., 1999). The contribution of GABA to cortical neuron survival is again linked to its depolarizing or hyperpolarizing activity. Global NKCC1 inactivation reduces developmental cell death of transient layer 1 Cajal-Retzius (CR) neurons (Blanquie et al., 2017a), and hyperpolarization promotes subtype-specific survival of CR neurons (Riva et al., 2019). Inhibiting activity altogether in developing pyramidal cells significantly reduces their survival (Voigt et al., 1997), and regional variations in apoptosis *in vivo* are partly regulated by corresponding differences in endogenous activity (Blanquie et al., 2017b). *In vitro* evidence suggests, however, that it is not simply the presence or absence of activity that regulates apoptosis in developing cortical neurons; rather, distinct activity patterns may modulate survival, further supporting the notion that information encoded in the frequency of calcium signals can drive changes in cell behavior (Golbs et al., 2011; Wong Fong Sang et al., 2021). Investigating how ion channel subunit composition and localization contribute to activity patterns *in vivo* and how these patterns activate specific calcium-dependent signaling pathways in developing cortical neurons to regulate survival is an essential next step.

Notably, the emergence of synchronous calcium transients within populations of cortical pyramidal and hippocampal neurons is positively correlated with survival (Heck et al., 2008; Murase et al., 2011; Voigt et al., 1997). Such synchronous transients in neuronal domains are partially mediated by gap junctions (Kandler and Katz, 1998; Yuste et al., 1992), and pharmacological gap junction inhibition preserves spontaneous asynchronous activity but increases neuronal apoptosis (Heck et al., 2008). In line with this, multiple calcium-dependent transcription factors have been linked to neuronal survival, including MEF2 (Liu et al., 2003; Mao et al., 1999) and NFATc4 (Benedito et al., 2005; Quadrato et al., 2012).

Increased activity also correlates with improved postnatal survival of cortical interneurons (Denaxa et al., 2018; Priya et al., 2018; Wong et al., 2018, 2022). Interneurons that die are less likely to participate in coordinated network events, displaying fewer synchronized calcium fluctuations *in vivo* compared with cells that survive (Duan et al., 2020; Wong et al., 2018). Accordingly, artificially hyperpolarizing interneurons decreases their survival, whereas increasing their activity promotes survival (Denaxa et al., 2018; Priya et al., 2018). Interestingly, the contribution of activity-dependent signaling to interneuron survival, thought to be mediated at least partly through the calcium-sensitive CaN/NFAT pathway (Priya et al., 2018), is subtype-specific (Denaxa et al., 2018; Priya et al., 2018). The activity of maturing neuronal populations also non-cell autonomously regulates interneuron subtype survival to shape developing circuits (Wong et al., 2018, 2022). Glutamatergic signaling is required for activity-dependent survival of neurogliaform and basket cells, whereas bipolar cells rely on serotonin to modulate activity-dependent survival (Wong et al., 2022). It will be interesting to determine the calcium-dependent pathways that link electrical signals to the survival of specific neuronal subtypes.

### Differentiation

Calcium and electrical activity play central roles in regulating aspects of neuronal identity. We restrict our focus here to a brief discussion of calcium functions in early events underlying cortical neuron differentiation, namely the acquisition of fate determinants and initial elaboration of neurites. The involvement of activity-dependent signaling in later events, such as synaptogenesis, circuit integration, maturation and plasticity, have been extensively reviewed elsewhere (Antón-Bolaños et al., 2018; Greer and Greenberg, 2008; Molnár et al., 2020; Pan and Monje, 2020; Rosenberg and Spitzer, 2011).

Pioneering studies in *Xenopus* spinal neurons demonstrated that intracellular calcium elevations regulate distinct aspects of differentiation, including neurotransmitter specification, maturation of ionic conductances and synaptic development (Borodinsky and Spitzer, 2007; Gu and Spitzer, 1995). In the developing cortex, expression of neuronal fate determinants may be calcium-regulated. Elevated calcium in differentiating human iPSC-derived TS cortical neurons biases neuronal production *in vitro* (Paşca et al., 2011). Moreover, *in vivo* gain and loss of function of the L-type VGCC Ca_v_1.2 in differentiating NSPCs bidirectionally modulates the relative abundance of cells expressing markers of callosal or subcerebral projection neurons in a calcium-dependent manner (Panagiotakos et al., 2019)*.* GABA_A_R-dependent activation of L-type VGCCs also controls the morphological differentiation of pyramidal cells (McAllister et al., 1996; Redmond et al., 2002; Wayman et al., 2006; reviewed by Chen and Ghosh, 2005), and premature KCC2 expression *in vivo* reveals that GABA depolarization is necessary for dendritic maturation of cortical neurons (Cancedda et al., 2007). In addition, elevating neuronal activity and spontaneous calcium transient frequency in migratory neurons arrests migration and induces precocious dendritic branching (Bando et al., 2016). Consistent with this, thalamocortical afferent activity is indispensable for proper development of neuronal morphology (Callaway and Borrell, 2011; Li et al., 2013), as well as barrel column formation in mice (Antón-Bolaños et al., 2019; Li et al., 2013). Recently, a small molecule chemical screen in human iPSC-derived cortical neurons identified epigenetic modifiers and calcium signaling activators as enhancers of neuronal maturation, including neurite elaboration, further supporting that facilitating calcium-dependent gene expression can promote terminal differentiation of cortical neurons (Ciceri et al., 2022 preprint; Hergenreder et al., 2022 preprint).

Calcium-regulated transcription factors or transcriptional activators, including CREB (Redmond et al., 2002; Wayman et al., 2006), NEUROD (Gaudillière et al., 2004) and CREST (Aizawa et al., 2004), have also been implicated in dendrite development. In addition, a CREB-dependent microRNA (miR132) positively regulates cortical neuron neurite outgrowth (Vo et al., 2005). Callosal axon outgrowth in the developing cortex is impeded by suppressing neuronal activity (Mizuno et al., 2007; Rodríguez-Tornos et al., 2016; Suárez et al., 2014; Wang et al., 2007), and axonal pathfinding has been linked to intracellular calcium signaling involving kinases and phosphatases with distinct calcium sensitivities (reviewed by Gomez and Zheng, 2006). How these calcium-dependent mechanisms cooperate to control axonal and dendritic elaboration in the developing cortex remains unclear. Intriguingly, a calcium-independent interaction between Ca_v_1.2 and RhoA was shown to regulate dendritic morphogenesis (Krey et al., 2013), highlighting additional roles for VGCCs as anchors for large signaling complexes at the membrane.

Electrical activity and neurotransmitter signaling regulate cortical interneuron differentiation in a calcium-dependent manner. BDNF, in conjunction with depolarization, enhances dendritic branching and electrophysiological maturation of parvalbumin (PV)-expressing interneurons (Berghuis et al., 2004). Postnatal electrical activity mediated by ionotropic glutamate receptors is also required for morphological maturation of calretinin- and reelin-expressing cortical interneurons (De Marco García et al., 2011). The activity-regulated DNA binding protein SATB1, which is associated with postnatal survival of somatostatin (SST)-expressing MGE-derived interneurons, is necessary for their maturation and terminal differentiation (Close et al., 2012; Denaxa et al., 2012). KCl-induced SATB1 upregulation is dependent on both calcium influx through L-type VGCCs and GABAR activation (Denaxa et al., 2012). More recently, the calcium-dependent transcription factor MEF2C was found to be required for the differentiation of PV-expressing cortical interneurons (Mayer et al., 2018). Activity-dependent splicing regulators such as the ASD-relevant RNA binding protein RBFOX1 (Table S1), which promote neuronal differentiation of cortical NSPCs (Zhang et al., 2016), also regulate distinct aspects of PV- and SST-expressing interneuron maturation and connectivity (Wamsley et al., 2018). These data reveal that activity-dependent calcium signaling is essential for the acquisition of molecular identity and morphology in developing pyramidal and interneuron populations.

### Calcium and gliogenesis

**Fig. 4.**
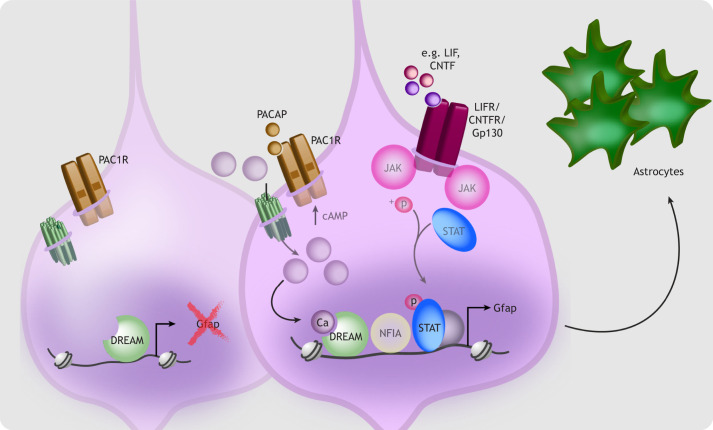
**Calcium and astrogliogenesis.** Although microglia and embryonic oligodendrocytes originate largely outside of the cortex, RGCs (light purple) transition from making neurons to generating cortical astrocytes (green) during mid-to-late gestation in the rodent. This gliogenic switch begins at approximately embryonic day 17 in mice and continues postnatally. Activation of plasma membrane receptors [glycoprotein 130 (Gp130; Il6st), ciliary neurotrophic factor receptor (CNTFR) and leukemia inhibitory factor receptor (LIFR)] regulates *Gfap* transcription and astroglial differentiation via JAK/STAT signaling. In parallel, pituitary adenylate cyclase activating polypeptide (PACAP)-mediated activation of its receptor, PAC1R, generates cAMP-dependent calcium elevations. Calcium binds DREAM to promote expression of astrocyte-specific genes and astrogliogenesis.

To ensure that neurogenesis proceeds faithfully during early cortical development, RGCs actively inhibit intrinsic mechanisms of astrogliogenesis, including transcription of astrocyte-specific genes (reviewed by Miller and Gauthier, 2007). Extrinsic signals acting via neurotrophic factors later stimulate cortical RGCs to become gliogenic (Qian et al., 1997; Song and Ghosh, 2004), in part via JAK/STAT pathway activation (Bonni et al., 1997). G protein signaling initiated by pituitary adenylate cyclase-activating polypeptide (PACAP, encoded by *Adcyap1*) and transduced via calcium has been identified as a complementary mechanism involved in the onset of astrogliogenesis (Fig. 4) (Nishimoto et al., 2007; Vallejo and Vallejo, 2002). Activation of PAC1 receptors by PACAP induces cyclic AMP (cAMP) production, which promotes astrocyte differentiation (Cebolla et al., 2008; McManus et al., 1999; Vallejo and Vallejo, 2002). PACAP stimulation elicits gradual intracellular calcium rises in NSPCs, whereas cAMP antagonism eliminates these rises and blocks astrocyte differentiation (Cebolla et al., 2008). During astrogliogenesis, calcium binding to the downstream regulatory element antagonist modulator (DREAM) is required for expression of the astrocyte-specific glial fibrillary acidic protein (*Gfap*) gene (Cebolla et al., 2008). These data indicate that PACAP induces cAMP-dependent calcium entry, enabling calcium-dependent astrocyte-specific gene transcription (Cebolla et al., 2008; McManus et al., 1999; Vallejo and Vallejo, 2002). PACAP signaling in mice lacking *Dream* (*Kcnip3*) fails to induce astrocyte differentiation, but this can be rescued by JAK/STAT activation, suggesting that calcium-regulated DREAM signaling works in parallel to JAK/STAT signaling during astrogliogenesis (Cebolla et al., 2008).

Neural activity and downstream calcium signaling is also implicated in the development and function of cortical oligodendrocyte precursor cells (OPCs) and microglia (Box 3). Dissecting how activity-dependent calcium signaling facilitates communication between developing neurons and glia will enhance our understanding of how the cortex is built.

**Box 3. Calcium and embryonic glial cells originating outside the cortex**

Embryonic oligodendrocyte precursor cells (OPCs) initially populate the cortex through two waves of migration from the ganglionic eminences (Kessaris et al., 2006). A third group of OPCs is generated in the postnatal cortex, replacing a subset of ventrally-derived OPCs (Kessaris et al., 2006). During these developmental windows, OPCs begin to express ion channels and neurotransmitter receptors that enable them to respond to activity-dependent signals (De Biase et al., 2010; Fulton et al., 2010; Spitzer et al., 2019; reviewed by Bergles and Richardson, 2015). It was first demonstrated in the rodent optic nerve that inhibiting electrical activity reduces the number of mitotic OPCs (Barres and Raff, 1993). Pharmacological, electrophysiological and optogenetic manipulations in the cortex have since reinforced the notion that neuronal activity regulates OPC proliferation and myelination (Demerens et al., 1996; Gary et al., 2012; Gibson et al., 2014; Mitew et al., 2018). The effects of electrical activity on OPCs are likely at least partly transduced via VGCCs, as Ca_v_1.2 loss of function results in impaired OPC proliferation, axon-OPC interactions and myelination (Cheli et al., 2015, 2016). Live imaging of the developing zebrafish spinal cord also reveals that neuronal activity induces different patterns of calcium signals to regulate myelination (Baraban et al., 2017; Krasnow et al., 2018). What initiates these dynamics and how they influence genetic programs driving OPC differentiation and myelination remains unclear. Although OPCs differentially respond to activity-dependent signals in the spinal cord (Marisca et al., 2020), such OPC functional heterogeneity in the developing cortex remains poorly understood.

Microglia originate outside the CNS and migrate into the cortex during embryonic development (Ginhoux et al., 2010; reviewed by Thion and Garel, 2017), where they play important roles in regulating progenitor abundance and sculpting developing circuits (Cunningham et al., 2013; Squarzoni et al., 2014; Ueno et al., 2013; reviewed by Thion and Garel, 2020). Although various neurotransmitters, immune molecules and ligands for purinergic receptors induce microglial calcium rises *in vitro* (reviewed by Umpierre and Wu, 2021), it remains less clear how calcium influences the functions of cortical microglia. Neuronal activity regulates microglial-mediated synaptic pruning (Schafer et al., 2012), and microglia sensitive to GABA preferentially remodel inhibitory synapses in the postnatal cortex (Favuzzi et al., 2021). In the developing zebrafish spinal cord, activity-dependent myelin phagocytosis by microglia is characterized by spontaneous calcium transients in microglial processes contacting myelin (Hughes and Appel, 2020). Future studies probing how activity and calcium facilitate neuron-glia communication during development will aid our understanding of how different cell types function in concert to build the cortex.

## Conclusions

As we have reviewed here, the coordinated spatiotemporal regulation of calcium signaling directs cellular behaviors that underlie early cortical development, including aspects of NSPC function. Calcium is a key mediator of activity-dependent gene expression, serving as a hub linking environmental cues to the cytoskeleton, metabolic pathways and other biochemical cascades. Underscoring these crucial developmental roles, genetic studies reveal that mutations in genes encoding calcium signaling modulators contribute to the pathophysiology of neurodevelopmental disorders. There remain, however, open questions about how activity and calcium-dependent processes are regulated at a cell type-specific level and how this regulation coordinates cellular behaviors during corticogenesis. In other cell and tissue types, properties of intracellular calcium dynamics have been linked to activation of specific downstream transcription factors. It is thus tempting to hypothesize that distinct cell type-specific patterns of electrical signals and calcium elevations in the embryonic cortex may subserve specific developmental roles. A natural corollary to this hypothesis is that disrupting calcium signaling and patterned electrical activity in specific cortical populations during development may promote neurodevelopmental disease.

Much remains to be elucidated about how calcium signaling is initiated and transduced in developing cortical cells. Titration of calcium signaling is not only achieved through dynamic regulation of ion channels and signaling proteins but also via coordination of extrinsic cues, including maternal hormones and metabolic regulation (Rash et al., 2018; Tyzio et al., 2006), which indirectly influence calcium homeostasis. How then do extrinsic signals converge to shape calcium entry in different cortical cell populations? How do different modes of calcium entry work cooperatively to direct gene expression programs across cell types? And how might intracellular calcium signals in one population influence dynamic interactions with other cell types? For example, do compartmentalized calcium signals that propagate across polarized cells, as reported in RGCs, influence local cellular processes (e.g. translation)? Single-cell, long-read and spatial sequencing technologies, organoid modeling, subcellular calcium and voltage imaging approaches, optogenetic and chemogenetic tools, and advances in labeling and isolating developing cortical cell populations will hopefully allow us to address these and other questions with unprecedented cellular resolution.

Determining how disease-relevant mutations affect intracellular signaling and cell type-specific developmental behaviors across space and time (reviewed by Panagiotakos and Pasca, 2022) will also enable the development of therapeutic approaches for neurodevelopmental diseases targeting calcium and activity-dependent mechanisms. Of special interest is determining how early disruption of activity-dependent signaling might cascade into later developmental events to promote disease. Recent findings support the notion that neurodevelopmental mechanisms may be reactivated to promote adult and aging-related disease states (Boxes 4 and 5), reinforcing the significance of exploring how calcium signals are normally regulated to coordinate the development of functional circuits. Looking ahead, it will be important to consider how changes at the organismal level (e.g. immune function, metabolism, gut-brain axis) may contribute to the regulation and misregulation of calcium in the embryonic and adult cortex to better understand normal development and the emergence of neurological disorders.

**Box 4. Reactivation of developmental calcium signaling mechanisms in glioma**

In primary glioma specimens from adult human patients, stem-like glioma cells display molecular signatures reflecting neural stem and progenitor cell (NSPC) identity (Bhaduri et al., 2020; Venteicher et al., 2017; Wang et al., 2020), and their morphology, behavior and lineage trajectory resembles that of embryonic radial glial cells (RGCs) (Bhaduri et al., 2020; Couturier et al., 2020; Wang et al., 2020). Similarities between cortical RGCs and stem-like glioma cells extend into calcium signaling dynamics, suggesting that calcium-regulated developmental mechanisms may be reused to promote glioma initiation and maintenance. For example, patient-derived glioblastoma xenografts in mice display synchronous calcium transients (Venkataramani et al., 2019; Venkatesh et al., 2019) and, as in cortical radial glia, calcium and electrical activity modulates glioma cell proliferation (Urso et al., 2019; Venkatesh et al., 2019; Zhang et al., 2012). Adjacent glioma cells are connected via ‘microtubes’ composed of gap junctions (Osswald et al., 2015), and calcium propagates across coupled cells in a manner reminiscent of calcium waves in the developing cortical ventricular zone (Osswald et al., 2015; Venkataramani et al., 2019). It is plausible that unraveling the calcium signaling mechanisms directing embryonic NSPC proliferation and migration will provide crucial insights into glioma cell biology to identify potential therapeutic targets for the management of glioma invasion and progression.

**Box 5. Implications for neurodegenerative diseases**

Calcium signaling deregulation has been implicated in neurodegenerative disorders, including Alzheimer's disease (AD) (reviewed by Pchitskaya et al., 2018; Verkhratsky, 2019). In human induced pluripotent stem cell models of AD and frontotemporal lobar degeneration-tauopathy, activity-dependent calcium elevations are abnormally high (Imamura et al., 2016; Park et al., 2018). Neurons and glia also display elevated basal calcium in AD mouse models (reviewed by Pchitskaya et al., 2018; Verkhratsky, 2019), and genes related to increased cytosolic calcium are enriched in patients with heightened risk for sporadic AD (Heck et al., 2015). Intriguingly, calcium- and activity-dependent pathways altered in neurodevelopmental diseases are thought to be affected in AD (Ivashko-Pachima et al., 2021; Mencer et al., 2021). Key calcium signaling effectors like DYRK1A (Table S1) are deregulated both in neurodevelopmental disorders and cortices of sporadic AD patients (Ferrer et al., 2005). Consistent with this, aging and AD rodent models exhibit aberrant calcium-dependent CaN/NFAT signaling (Foster et al., 2001; Norris et al., 2005), and blocking CaN/NFAT in AD models improves synaptic function, amyloid pathology and astrogliosis (Furman et al., 2012).

Emerging studies also suggest that individuals with neurodevelopmental disorders such as autism spectrum disorder (ASD) and Down syndrome (DS) have increased risk of developing AD and related dementias early in life (Vivanti et al., 2021; reviewed by Lott and Head, 2019). Although, in the case of DS, association with early-onset dementia is related to increased amyloid precursor protein (APP) resulting from trisomy of all or part of chromosome 21, multiple genetic and environmental factors likely contribute to increased AD susceptibility. Deregulation of electrical activity, which impinges on calcium, represents one possible contributing mechanism common to neurodevelopmental and neurodegenerative disorders. In DS and ASD mouse models, abnormalities in NKCC1/KCC2 expression promote persistent depolarizing activity of GABA, akin to the developing brain, in adult and postnatal hippocampal neurons (Deidda et al., 2015; Tyzio et al., 2014). Consequently, administration of the NKCC1 antagonist bumetanide in these models restores chloride gradients and rescues cognitive and behavioral abnormalities (Deidda et al., 2015; Tyzio et al., 2014). Intriguingly, bumetanide was recently shown to improve pathological and behavioral deficits in an AD rodent model (Taubes et al., 2021). Furthermore, APP has been linked to the regulation of KCC2, GABAR and VGCC expression (Chen et al., 2017; Doshina et al., 2017; Yang et al., 2009), implicating it as a regulator of electrical activity and calcium homeostasis.

## Supplementary Material

10.1242/develop.198853_sup1 Supplementary information
