# Supplementary material for: Calcium and activity-dependent signaling in the developing cerebral cortex
Source: Development. 2022 Sep 14;149(17):dev198853. doi: 10.1242/dev.198853 (PMC9578689; doi:10.1242/dev.198853)
Supplement: Supplementary information [file develop-149-198853-s1.pdf]

Table S1. Developmental disease-associated mutations intersect with calcium signaling.

Select genes from the SFARI Gene Database encode protein products that either directly regulate calcium homeostasis and signaling or indirectly impinge on calcium by modulating neuronal excitability or altering membrane potential. We restricted our survey to genes defined as either Category 1 (high confidence) or Category 2 (strong candidate) (see: https://gene.sfari.org/about-gene-scoring/ for the SFARI gene scoring criteria) and list the developmental functions of their gene products and the functional effects (when known) of select disease-associated mutations. This Table only highlights selected genes and is not exhaustive in scope. Notably, due to space constraints, we have not included synaptic structural proteins that regulate ion/calcium channel localization and function at synapses [e.g., ANK2 (Kline et al., 2014), NRXN1-3 (Luo et al., 2020; Missler et al., 2003)], which have been reproducibly implicated in ASD.

Gene	Mutation type	Disease	Relationship to calcium/activity dependent signaling	Electrophysiological characterization of select variants and/or select developmental phenotypes	References
			**CALCIUM CHANNELS**		
ATP2B2	missense, nonsense, truncating variants,	ASD, epilepsy	encodes the PMCA2 ATPase calcium pump	> LoF mutations predicted to result in excess intracellular calcium;<br>> ASD associated variants (e.g., Cys756*, Trp1064* - predicted to lead to nonsense mediated decay);<br>> heterozygous LoF mice: heightened amplitude AMPAR-mediated calcium transients in cerebellar Purkinje cells; increased expression of P/Q-type VGCCs; increased amplitude depolarization-induced calcium influx, decreased Purkinje cell numbers;<br>> KO mice: reduced growth rate, balance and movement problems, deafness and vestibular problems	***Select genetic studies:*** *Carayol et al. 2011; Takata et al., 2018; Iossifov et al. 2014; Yang et al., 2013; Prandini et al., 2012*<br>***Select functional references:*** *Smits et al., 2019; Fakira et al., 2012; Kozel et al., 1998*
CACNA1A	missense, frameshift, stop-gain and LoF variants	ASD, ID, epilepsy (e.g., EE, primary generalized epilepsy), paroxysmal movement disorders; FHM	encodes the pore forming subunit of the VGCC Cav2.1	> GoF mutations (e.g. R192Q mutation implicated in FHM): knock-in mice exhibit elevated calcium current density in cerebellar granule cells, increased neurotransmitter release at neuromuscular synapses, enhanced induction of cortical spreading depression;<br>> epilepsy related mutations: dominant negative, impaired channel function (e.g. C5733T, E147K);<br>> KO mice: ataxia, dystonia and early death, irregular corticothalamic activity;<br>> interneuron-specific LOF: epilepsy, abnormal GABA release from PV+ cells;<br>> pyramidal neuron specific LOF: attenuates excitability	***Select genetic studies:*** *Gulsuner et al., 2020; Epi4k Consortium 2016; Hamdan et al., 2017; Damaj et al., 2015; Jiang, 2015; Jouvenceau et al., 2001; Imbrici et al., 2004; Stam et al., 2009; commentary in Noebels et al., 2002* ***Select functional references:*** *Bomben et al., 2016; Rossignol et al., 2013; Llinás et al., 2007; Todorov et al., 2006; van den Maagdenberg et al., 2004; Kaja et al., 2005; commentary in Noebels et al., 2002*
CACNA1C	missense variants, G406R point mutation in *exon 8a*	classical TS, SCZ, BPD	encodes the pore forming subunit of the VGCC Cav1.2	> GoF TS mutation: impaired channel splicing, loss of voltage-dependent channel inactivation, increased depolarization-dependent calcium elevations, altered cortical projection neuron differentiation, activity-dependent dendritic retraction, deficits in cortical excitatory and inhibitory neuron migration	***Select genetic studies:*** *Splawski, et al. 2004; SCZ Psychiatric GWAS consortium 2011; Cross-disorder group of Psychiatric Genomics Consortium, 2013; SCZ Working Group of the Psychiatric Genomics Consortium, 2014; Ripke, et al. 2013; Ferreira et al, 2008; Purcell, et al. 2014, Green et al., 2010; Green et al., 2013*<br>***Select functional references:*** *Splawski, et al. 2004; Barrett & Tsien, 2008; Pasca et al., 2011; Krey et al., 2011; Birey et al., 2017; Kamijo et al., 2018; Panagiotakos et al., 2019; reviewed by Bhat et al., 2012*
CACNA1D	missense, LOF, and GoF variants	ASD, epilepsy	encodes the pore forming subunit of the VGCC Cav1.3	> GoF mutations: impaired/irregular voltage-dependent channel inactivation (e.g., G407R, G403D, G403R, Q547H, P1336R, A769G, V401L and S652L); hyperpolarized voltage dependence of activation (e.g., V259D, F747L, I750M, V1153G, A769G, V401L and S652L); increased current density (e.g. V401L);<br>> LoF mutations: increased sensitivity to dihydropyridine inhibition (e.g. S652L);<br>> ASD-associated mutation A760G: reduced calcium dependent inactivation, altered channel gating leading to increased intracellular calcium<br>> KO mice: deafness, cardiac irregularities	***Select genetic studies:*** *O'Roak et al., 2012; Klassen et al., 2011*<br>***Select functional references:*** *Pinggera et al., 2015, 2017, 2018; Hofer et al., 2020; reviewed by Ortner et al., 2020; Baig et al. 2011; Scholl et al. 2013; Limpitikul et al., 2016; Platzer et al., 2000*
CACNA1E	missense, synonymous, and GoF/LoF variants	ASD, DEE	encodes the pore forming subunit of the VGCC Cav2.3	> G1209S: top de novo ASD risk mutation;<br>> GoF mutations: slow inactivation, hyperpolarizing shifts in voltage dependent activation, increased current density (e.g. majority of DEE variants in pore domains, S6 variants: F698S, I701V, A702T; S4-5 variant: I603L);<br>> KO mice: CA1 pyramidal cells from KO mice exhibit increased excitability, and altered action potential properties; neurons of the reticular thalamus have significantly reduced high voltage activated calcium currents, and suppressed rhythmic burst discharges.	***Select genetic studies:*** *O'Roak et al., 2012; Takata et al., 2016; Helbig et al., 2018; comment in Carvill et al., 2019; Neale et al., 2012*<br>***Select functional references:*** *Helbig et al., 2018, Zaman et al., 2011, Neale et al., 2012; comment in Carvill et al., 2019; Gutzman et al., 2019*
CACNA1H	missense and LoF/GoF variants	ASD, epilepsy (e.g. CAE)	encodes the pore forming subunit of the VGCC Cav3.2	> missense LoF ASD mutations in voltage sensor (e.g., R212C, R902W), pore-forming domains (e.g., W962C), and c-terminus (e.g., R1871Q + A1874V): significantly reduced Cav3.2 (T-type) calcium currents;<br>> CAE associated variants result in GoF and LoF (e.g., C456S, G773D, R788C, V831M, E282K: altered voltage dependence of activation; F161L, P648L, G773D, R788C: altered voltage dependence of inactivation; C456S, A748V, D1463N: faster activation kinetics; G773D, G784S, R788C, V831M: slower activation kinetics; F161L: faster inactivation kinetics; G499S, P648L, G773D, R788C, V831M, G773D, V831M, G848S, R788C, G773D, R788C: slower inactivation/deactivation kinetics);<br>> modeling predicts net GOF/enhanced burst firing of thalamic neurons for CAE associated variants (e.g., C456S, P648L, G773D, R788C, G773D-R788C, A748V, G784S, G848S, and D1463N);<br>> modeling predicts decreased thalamic neuron burst firing for CAE associated variants (e.g., E282K and V831M);<br>> KO mice have anxiety-like phenotypes and impairments in memory	***Select genetic studies:*** *Chen et al., 2003; Splawski et al., 2006; Vitko et al., 2005; Heron et al., 2007*<br>***Select functional references:*** *Khosravani et al., 2004; reviewed by Perez-Reyes 2006; Vitko et al., 2005; Vitko et al., 2007; Heron et al., 2007; Gackiere et al., 2008, Tao et al., 2008; Becker et al., 2008; Powell et al., 2009; Hu et al., 2009; Gangarossa et al., 2014; Splawski et al., 2006*
CACNA2D3	missense variants, splice site mutations, deletions	ASD, epilepsy	encodes the auxiliary α2δ3 VGCC subunit	> deletion of *CACNA2D3* homolog *unc-36* in c-elegans: altered axon termination;<br>> *Cacna2d3* KO mice: impaired hearing, increased anxiety-related behaviors, reduced sensitivity to thermal pain	***Select genetic studies:*** *Iossifov et al., 2012; Girirajan et al., 2013; De Rubeis et al., 2014; C Yuen et al., 2017*<br>***Select functional references:*** *Buddell et al., 2019; Peng et al., 2021; Landmann et al., 2019; Pirone et al., 2014; Neely et al., 2010*
CACNB2	various missense variants	ASD	encodes the auxiliary β2 VGCC subunit	> GoF ASD-associated mutations (e.g., G167S, S197F): slower time dependent inactivation, altered sensitivity of voltage dependent inactivation, enhanced peak current densities;<br>> LoF ASD-associated mutations (e.g., F240L): accelerated time-dependent inactivation	***Select genetic studies:*** *Cross disorder group of the psychiatric genomics consortium 2013; Ripke et al., 2013; Cross disorder group of the psychiatric genomics consortium 2017; Pardinas et al., 2018, Yuen et al., 2015*<br>***Select functional references:*** *Breitenkamp et al., 2014; Graziano et al, 2021; Despang et al., 2020*
TRPC6	translocations, missense variants, premature stop variant	ASD	encodes a calcium permeable cation channel from the transient receptor potential family	> neural progenitor cells from ASD patient-derived iPSCs: reduced calcium influx;<br>> neurons from ASD patient-derived iPSCs: reduced TRPC6 protein levels, reduced dendritic spine density, altered synaptic properties;<br>> transgenic *Trpc6* GOF: increased dendritic spine density, enhanced spatial memory	***Select genetic studies:*** *Griesi-Oliveira et al., 2014*<br>***Select functional references:*** *Griesi-Oliveira et al. 2015; Zhou et al., 2008*
TRPM1	CNVs, deletions	ASD	encodes a calcium permeable cation channel from the transient receptor potential family	> *Trpm1* KO mice: hyperactive, attenuated anxiety-related behaviors, altered fear memory, abnormal social behavior	***Select genetic studies:*** *Girirajan et al., 2013; Matsunami et al., 2014*<br>***Select functional references:*** *Hori et al., 2021*
			**OTHER ION CHANNELS THAT IMPINGE ON CALCIUM ENTRY AND ACTIVITY DEPENDENT SIGNALING**		
CHRNA7	copy number variants, missense variants, microdeletion	Epilepsy, SCZ, DD, ID, ASD	encodes a calcium permeable nicotinic cholinergic receptor	> KO mice: phenotypes dependent on background strain due to differences in endogenous *Chrna7*;<br>> C3H KO mice: impaired LTP, abnormal auditory processing;<br>> C3H Het mice: reduced GAD65 levels, reduced GABAA receptors in male mice;<br>> reduced *CHRNA7* transcription and function (e.g., *CHRNA7* promoter variants); decreased calcium influx through CHRNA7 (e.g., both 15q13.3 deletions and duplications); increased ER stress (e.g., 15q13.3 duplications)	***Select genetic studies:*** *Mikhail et al., 2011; Shinawi et al,. 2009; Soler-Alfonso et al., 2014; Gillentine et al., 2017; Hoppman-Chaney et al., 2013; Leonard et al., 2002; Gault et al., 2003; Stephens et al., 2009*<br>***Select functional references:*** *Yin et al., 2017; Freund et al., 2016; Adams et al., 2012; Leonard et al., 2002; Sinkus et al., 2011; Gillentine et al., 2017*
KCNJ10	missense variants, synonymous variants, LoF/GoF variants	Seizures, ASD	encodes an ATP-sensitive potassium channel	> KO mice: motor impairments, hypomyelination, axonal abnormalities, retarded growth, dehydration, abnormalities in renal salt handling, premature death;<br>> astrocyte- and oligodendrocyte-specific cKO: seizures, premature death, ataxia, hindleg paralysis, retarded growth, abnormal astrocyte depolarization, impaired uptake of glutamate and potassium, reduced spontaneous neuronal activity, abnormalities in post-tetanic potentiation and short-term potentiation;<br>> oligodendrocyte-specific cKOs: mitochondrial abnormalities, axonal degeneration, loss of axonal integrity, neuronal loss, visual deficits, retinal abnormalities, motor problems, functional abnormalities including slow potassium clearance, delayed axonal recovery post stimuation, seizures;<br>> GoF mutations: enhanced membrane expression; increased current density (e.g., R18Q); pH-dependent current inhibition (e.g., R348H); increased current amplitudes (e.g., R18Q, V84M); increased unit conductance (e.g. V84M);<br>> homozygous missense mutations affecting transmembrane domain (e.g., G65P, G77R) modeled in *Xenopus*: decreased potassium currents	***Select genetic studies:*** *Ferraro et al.,2004; Buono et al., 2004; Sicca et al., 2011; Bockenhauer et al., 2009*<br>***Select functional references:*** *Bockenhauer et al., 2009; Neusch et al., 2001; Djukic et al., 2007; Schirmer et al., 2018; Larson et al., 2018; Sicca et al., 2011*
KCNMA1	missense variant, frame shift variant, GoF variants, LoF variant	Epilepsy, ASD, paroxysmal dyskinesia	encodes the pore-forming α-subunit of the large conductance calcium and voltage-activated potassium channel	> LoF mutations: altered resting membrane potential, decreased channel activity;<br>> KO mice: alterations in suprachiasmatic nucleus function and circadian rhythm, progressive hearing loss, cerebellar abnormalities, motor coordination dysfunction, reduced activity of Purkinje neurons, dysfunctional astrocyte/arteriolar smooth muscle communication;<br>> GoF mutations: enhanced voltage dependent activation and enhanced voltage sensitivity (e.g. N536H); in *Xenopus* oocytes: increased voltage- and calcium-dependent activation, faster activation kinetics in response to depolarizing voltage (e.g., D434G mutation); in CHO cells: increased sensitivity to calcium, activation at lower voltages, increase in open-channel probability (e.g., D434G)	***Select genetic studies:*** *Laumonnier et al., 2006; Neale et al., 2012; Liang et al., 2019; Zhang et al., 2020b; Satterstrom et al., 2020; Zou et al., 2021; Bailey et al., 2019; Du et al., 2005; Tabarki et al., 2016; Zhang et al., 2015*<br>***Select functional references:*** *Plüger et al., 2000; Díez-Sampedro et al., 2006; Meredith et al., 2006; Ruttiger et al., 2004; Sausbier et al., 2004; Filosa et al., 2006; Zhang et al., 2020b; Du et al., 2005*
GRIA2	nonsense variant, missense variant, deletions	ASD, ID, seizures, DEE, DD	encodes the GluA2 AMPAR subunit, whose inclusion in AMPARs renders them calcium-impermeable	> LoF mutations: decrease current amplitudes compared to wild type (WT) channels (e.g., G47E, D302G, del528-530, G609R, A639S, F644L, T646N);<br>> GoF mutations: increase current amplitudes compared to WT channels (e.g., Q607E);<br>> KO mice: increased calcium permeability in hippocampal neurons lacking GluA2, alterations in LTP, impaired exploratory behavior, alterations in reward behaviors	***Select genetic studies:*** *De Rubeis et al., 2014; Deciphering Developmental Disorders Study 2017; C Yuen et al., 2017; Hackmann et al., 2012*<br>***Select functional references:*** *Salpietro et al., 2019; Lu et al., 2009; Jia et al., 1996; Hackmann et al., 2012; Mead et al., 2003*
GRIN1	missense variants	ASD, Polymicrogyria, Epilepsy, ID, DD	encodes the GluN1 subunit of calcium permeable NMDARs	> LoF missense variants in or adjacent to a transmembrane domain (e.g., P557R, S560dup, G618R, G620R, Y647S, G815R, F817L, Q556*, G827R);<br>> GoF mutations: increased calcium currents through NMDARs (e.g., E662K);<br>> *Grin1* KO mice: significantly attenuated NMDA-induced calcium rises, abnormal social behaviors;<br>> *in utero* knockdown in developing cortex: deficits in radial migration;<br>> conditional *Grin1* deletion in PV+ cells: reduced LTP induction and maintenance, impaired social behavior;<br>> interneuron-specific *Grin1* deletion: attenuated NMDA currents;<br>> conditional *Grin1* deletion in pyramidal cells: increased social approach behavior;<br>> *Dlx5/6*-Cre mediated deletion *in utero*: abnormal morphological maturtion of Re+ interneurons and improper circuit integration	***Select genetic studies:*** *Epi4K Consortium et al., 2013; Hamdan et al, 2011; Jiang, 2015*<br>***Select functional references:*** *Hamdan et al, 2011; Gandal et al., 2012; Saunders et al., 2013; Lemke et al. 2016; commentary in Lemke, 2020; Forrest et al., 1994; Ferri et al., 2020; Billingslea et al., 2014; De Marco Garcia et al., 2015; Fry et al., 2018; reviewed by Vieira et al,. 2021*
GRIN2A	copy number variants, nonsense mutation, chromosome translocation breakpoints, missense variant	ASD, various epileptic syndromes (e.g., Landau-Kleffner syndrome, EE, childhood epilepsy, infantile onset EE), polymicrogyria, SCZ	encodes the GluN2A subunit of calcium permeable NMDARs	> GoF mutations: increased GluN1/GluN2A current density (e.g., R1067W); increased sensitivity to glutamate and/or glycine agonists (e.g., P552R, M817V, L812M, V452M, K669N, N447K, V506A, P699S); decreased magnesium block, increased current density (e.g., N447K); impaired zinc inhibition, normal current amplitude, glutamate and glycine affinities and open-state probabilities (e.g., A243V); increased total surface expression (e.g., K590N, K879R, R1067W);<br>> LoF mutations (e.g., R518H, T531M, D731N, V685G, G483R, A716T, C436R, C231Y, A548T, P79R, I694T, M705V, A727T, V734L, K772E, R370W); loss of magnesium block and decreased calcium permeability (e.g., N615K);<br>> *Grin2A* KO mice: altered NMDA/AMPA receptor currents in hippocampal neurons, impairments in LTP and LTD, increased locomotion, deficits in fear conditioning;<br>> *in utero* knock down: no effect of cortical radial migration	***Select genetic studies:*** *Barnby et al., 2005; Endele et al 2010; Lemke et al., 2013; Yoo et al., 2012; Liu et al., 2021; Singh et al., 2022*<br>***Select functional references:*** *Liu et al., 2021; Endele et al 2010; Strehlow et al., 2019; Kannagara et al., 2015; Sakimura et al. 1995; Kiyama et al. 1998; Chen et al., 2017; Ogden et al., 2017; Yuan et al., 2014; Marwick et al., 2019; Bertocchi et al., 2021; Xu et al., 2018; Swanger et al., 2016; Gao et al., 2017; Addis et al., 2017; Serraz et al., 2016; reviewed by Vieira et al., 2021*
GRIN2B	splice site variants, non-synonymous variants, LoF variants, *GRIN2B* GoF (West syndrome), chromosome translocation breakpoints, frameshift mutations, missense mutations,	ASD, SCZ, West Syndrome, ID, DD, Epilepsy	encodes the GluN2B subunit of calcium permeable NMDARs	> GoF mutations: loss of voltage dependent magnesium block (e.g., N615I, V618G); reduced calcium permeability (e.g., N615I);<br>> LoF GluN2B-724 truncation mutant: attenuated calcium influx, impaired trafficking to cell membrane, impaired dendritic morphogenesis;<br>> LoF mutations: mutation in ligand binding domain, impaired glutamate binding, higher glutamate concentrations required for activation (e.g., G689S, G689C), poor expression of G689C variant at cell membrane; overexpression in hippocampal neurons: decreased frequency of NMDAR-dependent mEPSCs with faster deactivation kinetics, no change in frequency of AMPAR-dependent mEPSCs;<br>> KO GluN2B to GluN2A "replacement" mouse: altered synaptic plasticity/development, reduced social exploration, hyperlocomotion;<br>> MADM-mediated *Grin2*b KO: impaired dendritic pruning and patterning in DG granule cells and spiny stellate cells;<br>> Dlx5/6-Cre mediated *Grin2b* deletion: abnormal morphological maturation of Re+ interneurons<br>> GluN2B overexpression in mice: altered synaptic function, enhanced memory/learning	***Select genetic studies:*** *De Rubeis et al., 2014; Iossifov et al., 2015; Ohtsuki et al., 2001; Myers et al., 2011; Lemke et al., 2014; Epi4K Consortium et al., 2013; Endele et al., 2010; Zhao et al.,2011; Tarabeux et al., 2011; O'Roak et al., 2011; O'Roak et al., 2012; Kellner et al., 2021*<br>***Select functional references:*** *Lemke et al., 2014; Swanger et al., 2016; Tang et al., 1999; Freunscht et al., 2013; Fedele et al., 2018; Akashi et al,. 2009; Wang et al., 2009; Wang et al., 2011; Kellner et al., 2021; Bahry et al., 2021; Sceniak et al., 2019; De Marco Garcia et al., 2015; Jiang et al., 2015a; Espinosa et al., 2009; reviewed by Vieira et al., 2021*
GABRB2	missense variants, LoF mutations	ASD, epilepsy (e.g., CAE, DEE, EME)	encodes the β2 subunit of the GABAA receptor, activation elicits calcium influx in cortical NSPCs	> LoF disease-associated variants in N-terminal and transmembrane regions: reduced GABA-induced current amplitudes (e.g., I246T, V282A, I288S, P252L);<br>> *Gabrb2* KO mice: increased susceptibility to seizures and epilepsy, altered startle response, reduced anxiety/depression behaviors, hyperlocomotivity, social behavioral abnormalities, memory deficits	***Select genetic studies:*** *Srivastava et al., 2014b; Iossifov et al. 2014; Satterstrom et al., 2020; Yang et al., 2020; Hamdan et al., 2017*<br>***Select functional references:*** *el Achkar et al, 2021; Yeung et al., 2018*

Development • Supplementary information

GABRB3	de novo LoF mutations, missense mutations, intronic variants, synonymous mutations	ASD, epilepsy (e.g., CAE)	encodes the β3 subunit of the GABAA receptor; activation elicits calcium influx in cortical NSPCs	> LoF CAE variants: reduced current densities compared to wild type channels (e.g., P11S, S15F, G32R); reduced peak current amplitudes and reduced surface expression (e.g., P11S)	***Select genetic studies:*** *Epi4K Consortium et al., 2013; Chen et al., 2014; De Rubeis et al., 2014; Yang et al., 2017; Urak et al., 2006; Tanaka et al., 2008; Sanders et al., 2015; Jiang, 2015*<br>***Select functional references:*** *Delahanty et al., 2012; Urak et al., 2006; Tanaka et al., 2008; Tanaka et al., 2012*
KCNB1	LoF mutations, missense mutation, nonsense variant	DD, epilepsy (e.g., EE, West Syndrome), ASD	encodes the voltage-gated potassium channel Kv2.1	> V378A: altered subcellular localization, altered electrophysiological properties (retained voltage dependent activation with faster inactivation kinetics), altered ion selectivity;<br>> EE-associated mutations (e.g. S347R, G379R, T374I): deficits in ion selectivity;<br>> epilepsy-associated mutations (e.g. R306C, G401R): R306C - altered voltage sensitivity, reduced neuronal firing; G401R - dominant negative mutation in channel pore, G401R abolishes Kv2.1 currents and reduces neuronal firing;<br>> *Kcnb1* KO mice: hyperactivity, abnormalities in spatial learning, accelerated progression of seizures	***Select genetic studies:*** *de Kovel et al., 2017; Torkamani et al., 2014; Parrini et al., 2017; Thiffault et al., 2015; Srivastava et al., 2014a; Allen et al., 2016; Saitsu et al., 2015*<br>***Select functional references:*** *Torkamani et al., 2014; Thiffault et al., 2015; Speca et al., 2014; Saitsu et al., 2015*
KCNQ2	splice site variant, LoF mutations, missense variants, frameshift variants	ASD, BPD, epilepsy (e.g. DEE, benign familial neonatal seizures)	encodes the voltage gated potassium channel Kv7.2	> LoF mutations largely implicated in BFNS;<br>> voltage sensor S4 transmembrane segment mutations: impaired channel function, dominant negative effect at subthreshold voltages, depolarizing shift in activation curve (e.g., I205V and R213Q); reduced current amplitudes, increased channel protein levels (e.g., R213Q);<br>> pore forming S5 and S6 domain mutations: impaired channel function, dominant negative (e.g., A265P, T274M, G290D);<br>> c-terminal mutations: reduced potassium current amplitude (e.g., R532W, M518V);<br>> *Kcnq2* heterozygous null mice (modeling LoF pathogenic mutations): increased susceptibility to seizures and behavioral abnormalities including reduced sociability and hyperactivity;<br>> homozygous null *Kcnq2* LOF: increased pyramidal neuron excitability	***Select genetic studies:*** *Singh et al., 1998; Jiang et al., 2013; Milh et al., 2013; Judy et al., 2013, Borsotto et al., 2007; Lee et al., 2019; Jiang, 2015*<br>***Select functional references:*** *Biervert et al., 1998; Borsotto et al., 2007, Zhang et al., 2020a; Soh et al., 2014; Orhan et al., 2014*
KCNQ3	translocation mutations, missense variant	ASD, epilepsy (e.g., benign familial neonatal seizures)	encodes the voltage gated potassium channel Kv7.3	> LoF variants largely implicated in BFNS;<br>> GoF mutations linked to ASD and DD: channel GOF, not associated with seizures in patients (e.g., R230C, R230H, R230S, R227Q); voltage sensor mutations (e.g., R227 and R230);<br>> LoF mutations: voltage sensor mutations (e.g., M240R; homomeric channels not functional, mutation affects voltage sensitivity, predicted increase in channel resting state stability and destabilization of active state); significantly reduced current density, impaired channel function (e.g., R330L, R330C)	***Select genetic studies:*** *Hirose et al., 2000; Epi4K Consortium et al., 2013; Miceli et al., 2020; Grinton et al.,2015; Gilling et al., 2013; Charlier et al., 1998*<br>***Select functional references:*** *Miceli et al., 2015; Miceli et al., 2020; Gilling et al., 2013; Sands et al., 2019*
P2RX5	gene disrupting mutations, LoF variants	ASD	encodes a calcium permeable purinergic receptor	unknown?	***Select genetic studies:*** *Sanders et al., 2015; Iossifov et al. 2014*<br>***Select functional references:*** *Bo et al., 2003*
SCN1A	protein truncation mutations (LoF), frameshift, nonsense, splice-donor, missense mutations	Asperger Syndrome, ASD, FHM, epilepsy (Dravet Syndrome, Severe myoclonic epilepsy of infancy, GEFS+)	encodes the α subunit of the voltage-gated sodium channel Nav1.1	> *SCN1A* LoF mutations: Dravet syndrome;<br>> LoF mutations: reduced current amplitude, hypersensitivity to steady-state inactivation (e.g., S1328P); abolished sodium currents in HEK cells (e.g., E78X, W384X, E1587K, and R1596C); partial LOF, abnormal recovery after inactivation, reduced sodium current density (e.g., E788K and M909K); slow inactivation recovery, hyperpolarizing shifts in activation/inactivation (e.g., D249E); reduced current density, slow recovery after inactivation (e.g., T1934I), slow recovery from inactivation (e.g., E78D);<br>> interneurons from iPSCs derived from individuals with Dravet Syndrome (heterozygous S1328P mutations): sodium current and AP firing deficits; no electrophysiological abnormalities in excitatory neurons;<br>> mutations causing GEFS+ mainly missense mutations;<br>> select mutations linked to GEFS+ (functional effects influenced by cell type/expression system): increased Nav1.1 channel function in HEKs (D188V), increased Nav1.1 function in *Xenopus* oocytes (W1204R, R1648H, D1866Y); decreased Nav1.1 channel function in *Xenopus* oocytes (R859C, T875M); decreased Nav1.1 channel function in tsA201 (V1353L, I1656M, R1657C, A1685V);<br>> LoF in mice: epileptic seizures	***Select genetic studies:*** *Weiss et al. 2003; Escayg et al., 2000; Barela et al., 2006; Epi4K Consortium et al., 2013; Claes et al., 2001; Osaka et al., 2007; O'Roak et al., 2011; O'Roak et al., 2012; De Rubeis et al., 2014; C Yuen et al., 2017; Hamdan et al., 2017; Jiang et al., 2015b; Kluckova et al., 2020*<br>***Select functional references:*** *Weiss et al., 2003; Sun et al., 2016; Ogiwara et al., 2007; Barela et al., 2006; reviewed by Escayg and Goldin, 2010; Kluckova et al., 2020*
SCN2A	LoF variants (often associated with ASD), missense variants, GoF variants, protein truncating variants (ASD)	ASD, ID, epilepsy (infantile EE, benign infantile seizures), SCZ	encodes the α subunit of the voltage gated sodium channel Nav1.2	> Effects of mutations differ in mature and immature neurons owing to changes in subcellular distribution and splicing;<br>> LoF mutations: often associated with ASD and ID, impaired excitability in immature neurons and altered dendritic dendritic excitability in mature neurons;<br>> GoF mutations: mostly associated with infantile epilepsies, increased neuronal excitability;<br>> K1422E: mutation in selectivity filter, renders channel calcium permeable;<br>> R1902C: ASD-associated mutation, allows calcium-dependent conformational change in Nav1.2 when complexed with CaM;<br>> homozygous KO mice: perinatal lethality;<br>> heterozygous *Scn2a* LoF mice: impaired excitability and action potential initiation in immature neurons; impaired dendritic excitability and disrupted synaptic function and plasticity in mature neurons, with presence of immature dendritic spines; normal PV and SST neuron APs; hyperactivity, absence-like seizures, and learning delays	***Select genetic studies:*** *Hamdan et al., 2017; Jiang et al., 2015b; Sanders et al., 2012; Tavassoli et al., 2014; Jiang et al., 2013; Sundaram et al., 2013; Carroll et al., 2016*<br>***Select functional references:*** *Begemann et al., 2019; Spratt et al., 2019; Spratt et al., 2021; Zhang et al., 2021; Ben-Shalom et al., 2017; Heinemann et al., 1992; Echevarria-Cooper et al., 2022; Fromer et al., 2014; Kim et al., 2004; reviewed by Sanders et al., 2018*
SCN8A	various missense mutations	ASD, DD, ID, epilepsy (e.g. E1EE13)	encodes the voltage-gated α8-subunit of the sodium channel Nav1.6	> majority of patient mutations are GoF: increased channel activity due to alterations in opening and inactivation kinetics;<br>> LoF mutations: cognitive impairment, movement disorders;<br>> GoF mutations: heterozygous missense, increased neuronal excitability in postnatal rat hippocampal neuronal cultures (e.g., N1768D); enhanced channel activation in neurons in vitro, increased excitabillity and spontaneous activity (e.g., T767I); altered voltage dependence of activation, predicted to yield premature neuronal firing (e.g., N984K);<br>> heterozygous knock-in N1768D mice: seizures and sudden unexpected death in Epilepsy, dose dependent effects (homozygous knock-in mice: earlier onset of seizures and death);<br>> LoF mutations: abolishes sodium currents (e.g., G964R, E1218K); reduces protein abundance (e.g., E1218K); significantly reduced channel activity in HEKs, protein product stable and expressed at similar levels to WT, possible off target effects of mutant protein product (e.g., G1451S);<br>> *in vitro* LoF but possible *in vivo* GoF (e.g, R223G: reduced current density and at 37C reduces protein stability; similar activation/inactivation kinetics as WT at 30C with increased ramp current - possible GoF<br>> Conditional *Scn8a* deletion in inhibitory cells using Dlx5/6-Cre: absence epilepsy; no absence seizures with excitatory neuron-specific deletion	***Select genetic studies:*** *Epi4K Consortium et al., 2013; Veeramah et al., 2012; Blanchard et al., 2015; Estacion et al., 2014; de Kovel et al., 2014* ***Select functional references:*** *Veeramah et al., 2012; Wagnon et al., 2015; Blanchard et al., 2015; Estacion et al., 2014; Wagnon and Meisler 2015; de Kovel et al., 2014; Makinson et al., 2017*
SLC6A1	truncating variants, missense variants, splice site variants CNVs, insertions, deletions and synonymous variants, nonsense variant	epilepsy (myoclonic atonic, genetic generalized, non-acquired focal, Lennox-Gastaut syndrome), DD, ASD, ID, speech delay, SCZ	encodes the voltage-dependent GABA transporter protein type 1 responsible for sodium-dependent GABA reuptake	> LoF mutations associated with Lennox-Gastaut syndrome: reduced total protein levels in HEKs and rat cortical neurons, reduced cell surface expression, impaired GABA uptake (e.g., G234S);<br>> P361T: heterozygous LoF mutation associated with epilepsy and autism, lower total protein levels, mutant protein abnormally localized in ER, reduced protein function and surface expression;<br>> KO mice: homozygous LoF yields increased tonic inhibition, reduced phasic inhibition, reduced quantal GABA release, no change in GABA receptor density, tremor, walking abnormalities, decreased strength, seizures, and anxious behaviors; heterozygous LoF yields diminished GABA reuptake	***Select genetic studies:*** *Sanders et al., 2012, Satterstrom et al., 2020, Satterstrom et al., 2019; Cai et al., 2019; Carvill et al., 2015; Rees et al., 2020; Wang et al., 2020*<br>***Select functional references:*** *Cai et al., 2019; Carvill et al., 2015; Jensen et al., 2003; Cope et al., 2009; Chiu et al., 2005*
**SELECT CALCIUM-INTERACTING PROTEINS, CALCIUM SIGNALING MODULATORS, AND ACTIVITY-DEPENDENT PROTEINS**					
CREBBP	coding variants, point mutations, deletion, duplications, frameshift mutation, missense mutations, splice site mutations	ASD, Rubinstein-Taybi syndrome	encodes the calcium regulated transcriptional coactivator CBP, a CREB co-factor critical for activity-dependent gene expression	> CH1 domain mutant mice: deficits in social interaction, ASD-relevant repetitive behaviors, hyperactivity, and abnormal synaptic plasticity;<br>> *Gfap* -cre mediated cKO mice: microcephaly, behavioral anomalies, reduced embryonic NSPC proliferation, deficits in NSPC migration, impaired postnatal neurogenesis, and abnormal pyramidal cell morphology	***Select genetic studies:*** *Petrij et al., 1995; Roelfsema et al., 2005; Barnby et al., 2005; Wincent, et al. 2016; Coupry et al., 2002*<br>***Select functional references:*** *Merk et al., 2018; Zheng et al., 2016; Schoof et al., 2019; Valor et al., 2011*
CASK	missense variants, splice site variant, premature stop variants	ASD	encodes a calcium/calmodulin-dependent serine protein kinase	> iPSC-derived neurons from *CASK* mutation carriers: reduced CASK levels, alterations in presynaptic development, reduced spontaneous calcium events;<br>> neurons from *Cask* KO mice: increased frequency of spontaneous excitatory miniature synaptic events, decreased frequency of spontaneous inhibitory miniature synaptic events	***Select genetic studies:*** *Iossifov et al., 2014; Najm et al. 2008; Stessman et al. 2017; Becker et al., 2020; Mukherjee et al., 2020*<br>***Select functional reference:*** *Atasoy et al., 2007; Becker et al., 2020*
CIB2	copy number variants	ASD	encodes a calcium binding protein that mediates calcium signaling processes	> *Cib2* KO mice: hearing loss, loss of auditory hair cell currents;<br>> CIB2 down regulation in zebrafish and *Drosophila* : deafness and vision deficits	***Select genetic studies:*** *Prasad et al., 2012; Riazuddin et al., 2012*<br>***Select functional references:*** *Wang et al., 2017; Riazuddin et al., 2012*
DYRK1A	truncating variants, nonsense mutations, missense variants, frameshift mutations, splice site variants, copy number variants (DS), translocation	ASD, ID, microcephaly, DD	encodes a kinase that regulates localization and/or activity of calcium-dependent transcription factors (e.g. NFAT, CREB)	> *DYRK1A* in Down syndrome (DS) critical region, copy number increase implicated in DS;<br>> LoF *DYRK1A* mutations: microcephaly, ID and ASD;<br>> kinase domain mutations alter catalytic activity; R467Q: predicted function in protein stability; L245R: prevents DYRK1A autophosphorylation;<br>> LoF mutations (e.g., E396ter: protein product degraded by ubiquitin proteasome pathway, inactive kinase, no dominant negative effect; other predicted LoF mutations: I48K(fs*2), A498P(fs*94), E414V(fs*76) (loss of exon 11, kinase domain mutation);<br>> *in ovo* GoF in chick spinal cord: reduced proliferation; pharmacological LoF: increased proliferation and apoptosis;<br>> embryonic overexpression upregulates p27Kip1 in the embryonic chick spinal cord and mouse cortex;<br>> *in utero* GoF in mouse cortex: reduced proliferation, abnormal radial migration, enhanced neuronal differentiation, phenotypes dependent on timing and degree of over-expression;<br>> Trisomy of *Dyrk1a* (in mBAC TgDyrk1a mice): lengthened G1 and RG cell cycle length, altered numbers of neurons and IPCs during embryonic neurogenesis;<br>> *minibrain* (*Drosophila* ortholog): GoF promotes cell cycle exit, facilitates neuronal differentiation; haploinsufficiency results in microcephaly<br>> *Dyrk1a* homozygous KO mice: growth delays, die at midgestation; *Dyrk1a* heterozygous +/- mice: reduced brain size, increased neuronal density;<br>> cortex-specific *Dyrk1a* deletion: decreased cortical mass, reduced neuronal size, disrupted growth factor signaling; heterozygous mutants: changes in axonal projections and deeper layer neuron morphology; increased ASD-related behaviors;<br>> *in vitro* knockdown: altered dendritic growth and spine development	***Select genetic studies:*** *De Rubeis et al., 2014; Iossifov et al. 2015; Bronicki et al., 2015; Ji et al., 2015; Dang et al., 2018; Moller, et al. 2008; van Bon et al., 2016; Courcet, et al. 2012; van Bon et al., 2011; Lee et al., 2020; Iossifov et al. 2015; Deciphering Developmental Disorders 2015; Iossifov et al., 2012; Ruaud et al., 2015*<br>***Select functional references:*** *Tejedor, 1995; Dang et al., 2018; Fotaki, et al. 2002; Lee et al., 2020; Yabut et al., 2010; Evers et al., 2017; Shaikh et al., 2016; Fotaki et al., 2004; Hammerle et al., 2011; Levy et al., 2021; reviewed by Park and Chung, 2013; Guimera et al., 1996; Najas et al., 2015; Chakrabarti et al., 2007; Kurabayashi and Sanada, 2013*
MEF2C	nonsense mutation, deletions	ASD, DD, mental retardation	encodes a calcium-dependent member of the MADS box family of TFs	> genome wide analyses for TF binding sites: MEF2 controls many activity-dependent genes<br>> LoF mutations in hiPSC-derived cortical neurons: aberrant electrophysiological properties, including elevated synaptic activity and increased excitation;<br>> conditional *Mef2c* deletion in developing interneurons: reduced PV+ cell density, deficits in PV cell maturation;<br>> *Mef2c* cKO in excitatory lineage: attenuated cortical network activity and ASD-, ID- and schizophrenia-related behaviors;<br>> *Gfap* -Cre mediated *Mef2c* KO: increased excitatory synapse numbers, upregulated synaptic transmission, impairments in learning and memory;<br>> *Mef2c* cKO in NSPCs (*Nestin* -cre): abnormal cytoarchitecture/compaction of cells in CP, altered cortical organization;<br>> Mef2c KO mice: immature electrophysiological properties, reduced excitability;<br>> expression of superactivating MEF2C: decreased frequency of mEPSCs	***Select genetic studies:*** *Novara et al., 2010; Neale et al., 2012*<br>***Select functional references:*** *Mayer et al., 2018, Allaway et al., 2021; Harrington et al., 2016; Trudler et al., bioRxiv 2020; Li et al., 2008; Barbosa et al., 2008; Flavell et al., 2008*
RBFOX1	copy number variants	ASD, ID, epilepsy	encodes an RNA binding protein and activity-dependent splicing regulator	> encodes member of a family of splicing factors that regulate ion channels and synaptic proteins (e.g. *Cacna1c*);<br>> 160kb deletion in first exon and intron: reduced expression of *Rbfox1* mRNAs;<br>> knockdown: abnormal alternative splicing, abnormal neuroblast migration, impaired axon extension, impaired dendritic arborization, abnormal membrane and synaptic properties, increased neuronal excitability;<br>> interneuron specific cKO: abnormal alternative splicing, increased PV- and SST-expressing interneuron density, impaired interneuron activity (i.e., decreased mIPSC frequency and amplitude, increased cFos expression), increased seizure susceptibility, altered synaptic connectivity;<br>> CNS-specific knockdown: increased seizure susceptibility, increased neuronal excitability, and abnormal splicing	***Select genetic studies:*** *Hamada et al., 2016; Sebat et al., 2007; Martin et al., 2007; Bhalla et al., 2004; Mikhail et al., 2011; Davis et al., 2012; Zhao, 2013; Gandal et al., 2018; Davies et al., 2015*<br>***Select functional references:*** *Wamsley et al., 2018; Gehman et al., 2011; Martin et al., 2007; reviewed by Bill et al., 2013; Tang et al., 2009*
SRCAP	de novo LoF variants, truncating variants, frameshift variants, missense variants	ASD, FHS	encodes an activator of the calcium-sensitive transcriptional coactivator CREBBP	> FHS variants map to last two exons of SRCAP (33 and 34), DNA methylation profiles vary depending on the location of the mutation	***Select genetic studies:*** *Hood et al, 2012; Iossifov et al., 2014; Stessman et al., 2017; C Yuen et al., 2017; Rots et al., 2021; reviewed by Alonso-Gonzalez et al., 2018*

Footnote: Autism Spectrum Disorders (ASD); Benign Familial Neonatal Seizures (BFNS); Epileptic Encephalopathy (EE); Schizophrenia (SCZ); Intellectual Disability (ID); Developmental and Epileptic Encephalopathy (DEE); Childhood Absence Epilepsy (CAE); Developmental and Epileptic Encephalopathy (DEE); Developmental Delay (DD); Floating-Harbor syndrome (FHS); Timothy syndrome (TS); Bipolar Disorder (BPD); Episodic Ataxia Type 2 (EA2); Familial Hemiplegic Migraine (FHM); Generalized Epilepsy with Febrile Seizures Plus (GEFS+); Early-infantile EE Type 13 (EIEE13); Early Myoclonic Encephalopathy (EME)

Supplemental References

Adams, C. E., Yonchek, J. C., Schulz, K. M., Graw, S. L., Stitzel, J., Teschke, P. U. and Stevens, K. E. (2012). Reduced Chrna7 expression in mice is associated with decreases in hippocampal markers of inhibitory function: implications for neuropsychiatric diseases. *Neuroscience* **207**, 274-282.

Addis, L., Virdee, J. K., Vidler, L. R., Collier, D. A., Pal, D. K. and Ursu, D. (2017). Epilepsy-associated GRIN2A mutations reduce NMDA receptor trafficking and agonist potency – molecular profiling and functional rescue. *Sci. Rep.* **7**, 1-14.

Akashi, K., Kakizaki, T., Kamiya, H., Fukaya, M., Yamasaki, M., Abe, M., Natsume, R., Watanabe, M. and Sakimura, K. (2009). NMDA Receptor GluN2B (GluRε2/NR2B) Subunit Is Crucial for Channel Function, Postsynaptic Macromolecular Organization, and Actin Cytoskeleton at Hippocampal CA3 Synapses. *J. Neurosci.* **29**, 10869-10882.

Allaway, K. C., Gabitto, M. I., Wapinski, O., Saldi, G., Wang, C.-Y., Bandler, R. C., Wu, S. J., Bonneau, R. and Fishell, G. (2021). Genetic and epigenetic coordination of cortical interneuron development. *Nature* **597**, 693-697.

Allen, N. M., Conroy, J., Shahwan, A., Lynch, B., Correa, R. G., Pena, S. D. J., McCreary, D., Magalhães, T. R., Ennis, S., Lynch, S. A., et al. (2016). Unexplained early onset epileptic encephalopathy: Exome screening and phenotype expansion. *Epilepsia* **57**, e12-7.

Alonso-Gonzalez, A., Rodriguez-Fontenla, C. and Carracedo, A. (2018). De novo Mutations (DNMs) in Autism Spectrum Disorder (ASD): Pathway and Network Analysis. *Front. Genet.* **9**, 406.

Atasoy, D., Schoch, S., Ho, A., Nadasy, K. A., Liu, X., Zhang, W., Mukherjee, K., Nosyreva, E. D., Fernandez-Chacon, R., Missler, M., et al. (2007). Deletion of CASK in mice is lethal and impairs synaptic function. *Proc. Natl. Acad. Sci. U. S. A.* **104**, 2525-2530.

Bahry, J. A., Fedder-Semmes, K. N., Sceniak, M. P. and Sabo, S. L. (2021). An Autism-Associated de novo Mutation in GluN2B Destabilizes Growing Dendrites by Promoting Retraction and Pruning. *Front. Cell. Neurosci.* **15**, 692232.

Baig, S. M., Koschak, A., Lieb, A., Gebhart, M., Dafinger, C., Nürnberg, G., Ali, A., Ahmad, I., Sinnegger-Brauns, M. J., Brandt, N., et al. (2011). Loss of Ca(v)1.3 (CACNA1D) function in a human channelopathy with bradycardia and congenital deafness. *Nat. Neurosci.* **14**, 77-84.

Bailey, C. S., Moldenhauer, H. J., Park, S. M., Keros, S. and Meredith, A. L. (2019). KCNMA1-linked channelopathy. *J. Gen. Physiol.* **151**, 1173-1189.

Development • Supplementary information

Barbosa, A. C., Kim, M.-S., Ertunc, M., Adachi, M., Nelson, E. D., McAnally, J., Richardson, J. A., Kavalali, E. T., Monteggia, L. M., Bassel-Duby, R., et al. (2008). MEF2C, a transcription factor that facilitates learning and memory by negative regulation of synapse numbers and function. *Proc. Natl. Acad. Sci. U. S. A.* **105**, 9391-9396.

Barela, A. J., Waddy, S. P., Lickfett, J. G., Hunter, J., Anido, A., Helmers, S. L., Goldin, A. L. and Escayg, A. (2006). An epilepsy mutation in the sodium channel SCN1A that decreases channel excitability. *J. Neurosci.* **26**, 2714-2723.

Barnby, G., Abbott, A., Sykes, N., Morris, A., Weeks, D. E., Mott, R., Lamb, J., Bailey, A. J., Monaco, A. P. and International Molecular Genetics Study of Autism Consortium (2005). Candidate-gene screening and association analysis at the autism-susceptibility locus on chromosome 16p: evidence of association at GRIN2A and ABAT. *Am. J. Hum. Genet.* **76**, 950-966.

Barrett, C. F. and Tsien, R. W. (2008). The Timothy syndrome mutation differentially affects voltage- and calcium-dependent inactivation of CaV1.2 L-type calcium channels. *Proc. Natl. Acad. Sci. U. S. A.* **105**, 2157-2162.

Becker, A. J., Pitsch, J., Sochivko, D., Opitz, T., Staniek, M., Chen, C.-C., Campbell, K. P., Schoch, S., Yaari, Y. and Beck, H. (2008). Transcriptional upregulation of Cav3.2 mediates epileptogenesis in the pilocarpine model of epilepsy. *J. Neurosci.* **28**, 13341-13353.

Becker, M., Mastropasqua, F., Reising, J. P., Maier, S., Ho, M.-L., Rabkina, I., Li, D., Neufeld, J., Ballenberger, L., Myers, L., et al. (2020). Presynaptic dysfunction in CASK-related neurodevelopmental disorders. *Transl. Psychiatry* **10**, 312.

Begemann, A., Acuña, M. A., Zweier, M., Vincent, M., Steindl, K., Bachmann-Gagescu, R., Hackenberg, A., Abela, L., Plecko, B., Kroell-Seger, J., et al. (2019). Further corroboration of distinct functional features in SCN2A variants causing intellectual disability or epileptic phenotypes. *Mol. Med.* **25**, 6.

Ben-Shalom, R., Keeshen, C. M., Berrios, K. N., An, J. Y., Sanders, S. J. and Bender, K. J. (2017). Opposing effects on NaV1.2 function underlie differences between SCN2A variants observed in individuals with autism spectrum disorder or infantile seizures. *Biol. Psychiatry* **82**, 224-232.

Bertocchi, I., Eltokhi, A., Rozov, A., Chi, V. N., Jensen, V., Bus, T., Pawlak, V., Serafino, M., Sonntag, H., Yang, B., et al. (2021). Voltage-independent GluN2A-type NMDA receptor Ca2+ signaling promotes audiogenic seizures, attentional and cognitive deficits in mice. *Commun Biol* **4**, 59.

Bhalla, K., Phillips, H. A., Crawford, J., McKenzie, O. L. D., Mulley, J. C., Eyre, H., Gardner, A. E., Kremmidiotis, G. and Callen, D. F. (2004). The de novo chromosome 16 translocations of two patients with abnormal phenotypes (mental retardation and epilepsy) disrupt the A2BP1 gene. *J. Hum. Genet.* **49**, 308-311.

Bhat, S., Dao, D. T., Terrillion, C. E., Arad, M., Smith, R. J., Soldatov, N. M. and Gould, T. D. (2012). CACNA1C (Cav1.2) in the pathophysiology of psychiatric disease. *Prog. Neurobiol.* **99**, 1-14.

Biervert, C., Schroeder, B. C., Kubisch, C., Berkovic, S. F., Propping, P., Jentsch, T. J. and Steinlein, O. K. (1998). A potassium channel mutation in neonatal human epilepsy. *Science* **279**, 403-406.

Bill, B. R., Lowe, J. K., Dybuncio, C. T. and Fogel, B. L. (2013). Orchestration of neurodevelopmental programs by RBFOX1: implications for autism spectrum disorder. *Int. Rev. Neurobiol.* **113**, 251-267.

Billingslea, E. N., Tatard-Leitman, V. M., Anguiano, J., Jutzeler, C. R., Suh, J., Saunders, J. A., Morita, S., Featherstone, R. E., Ortinski, P. I., Gandal, M. J., et al. (2014). Parvalbumin cell ablation of NMDA-R1 causes increased resting network excitability with associated social and self-care deficits. *Neuropsychopharmacology* **39**, 1603-1613.

Birey, F., Andersen, J., Makinson, C. D., Islam, S., Wei, W., Huber, N., Fan, H. C., Metzler, K. R. C., Panagiotakos, G., Thom, N., et al. (2017). Assembly of functionally integrated human forebrain spheroids. *Nature* **545**, 54-59.

Blanchard, M. G., Willemsen, M. H., Walker, J. B., Dib-Hajj, S. D., Waxman, S. G., Jongmans, M. C. J., Kleefstra, T., van de Warrenburg, B. P., Praamstra, P., Nicolai, J., et al. (2015). De novo gain-of-function and loss-of-function mutations of SCN8A in patients with intellectual disabilities and epilepsy. *J. Med. Genet.* **52**, 330-337.

Bo, X., Jiang, L.-H., Wilson, H. L., Kim, M., Burnstock, G., Surprenant, A. and North, R. A. (2003). Pharmacological and biophysical properties of the human P2X5 receptor. *Mol. Pharmacol.* **63**, 1407-1416.

Bockenhauer, D., Feather, S., Stanescu, H. C., Bandulik, S., Zdebik, A. A., Reichold, M., Tobin, J., Lieberer, E., Sterner, C., Landoure, G., et al. (2009). Epilepsy, Ataxia, Sensorineural Deafness, Tubulopathy, and KCNJ10 Mutations. *N. Engl. J. Med.* **360**, 1960-1970.

Bomben, V. C., Aiba, I., Qian, J., Mark, M. D., Herlitze, S. and Noebels, J. L. (2016). Isolated P/Q Calcium Channel Deletion in Layer VI Corticothalamic Neurons Generates Absence Epilepsy. *J. Neurosci.* **36**, 405-418.

Borsotto, M., Cavarec, L., Bouillot, M., Romey, G., Macciardi, F., Delaye, A., Nasroune, M., Bastucci, M., Sambucy, J.-L., Luan, J.-J., et al. (2007). PP2A-Bgamma subunit and KCNQ2 K+ channels in bipolar disorder. *Pharmacogenomics J.* **7**, 123-132.

Breitenkamp, A. F. S., Matthes, J., Nass, R. D., Sinzig, J., Lehmkuhl, G., Nürnberg, P. and Herzig, S. (2014). Rare mutations of CACNB2 found in autism spectrum disease-affected families alter calcium channel function. *PLoS One* **9**, e95579.

Bronicki, L. M., Redin, C., Drunat, S., Piton, A., Lyons, M., Passemard, S., Baumann, C., Faivre, L., Thevenon, J., Rivière, J.-B., et al. (2015). Ten new cases further delineate the syndromic intellectual disability phenotype caused by mutations in DYRK1A. *Eur. J. Hum. Genet.* **23**, 1482-1487.

Buddell, T., Friedman, V., Drozd, C. J. and Quinn, C. C. (2019). An autism-causing calcium channel variant functions with selective autophagy to alter axon targeting and behavior. *PLoS Genet.* **15**, e1008488.

Buono, R. J., Lohoff, F. W., Sander, T., Sperling, M. R., O'Connor, M. J., Dlugos, D. J., Ryan, S. G., Golden, G. T., Zhao, H., Scattergood, T. M., et al. (2004). Association between variation in the human KCNJ10 potassium ion channel gene and seizure susceptibility. *Epilepsy Res.* **58**, 175-183.

C Yuen, R. K., Merico, D., Bookman, M., L Howe, J., Thiruvahindrapuram, B., Patel, R. V., Whitney, J., Deflaux, N., Bingham, J., Wang, Z., et al. (2017). Whole genome sequencing resource identifies 18 new candidate genes for autism spectrum disorder. *Nat. Neurosci.* **20**, 602-611.

Cai, K., Wang, J., Eissman, J., Wang, J., Nwosu, G., Shen, W., Liang, H.-C., Li, X.-J., Zhu, H.-X., Yi, Y.-H., et al. (2019). A missense mutation in SLC6A1 associated with Lennox-Gastaut syndrome impairs GABA transporter 1 protein trafficking and function. *Exp. Neurol.* **320**, 112973.

Carayol, J., Sacco, R., Tores, F., Rousseau, F., Lewin, P., Hager, J. and Persico, A. M. (2011). Converging evidence for an association of ATP2B2 allelic variants with autism in male subjects. *Biol. Psychiatry* **70**, 880-887.

Carroll, L. S., Woolf, R., Ibrahim, Y., Williams, H. J., Dwyer, S., Walters, J., Kirov, G., O'Donovan, M. C. and Owen, M. J. (2016). Mutation screening of SCN2A in schizophrenia and identification of a novel loss-of-function mutation. *Psychiatr. Genet.* **26**, 60-65.

Carvill, G. L. (2019). Calcium Channel Dysfunction in Epilepsy: Gain of CACNA1E. *Epilepsy Curr.* **19**, 199-201.

Carvill, G. L., McMahon, J. M., Schneider, A., Zemel, M., Myers, C. T., Saykally, J., Nguyen, J., Robbiano, A., Zara, F., Specchio, N., et al. (2015). Mutations in the GABA Transporter SLC6A1 Cause Epilepsy with Myoclonic-Atonic Seizures. *Am. J. Hum. Genet.* **96**, 808-815.

Chakrabarti, L., Galdzicki, Z. and Haydar, T. F. (2007). Defects in embryonic neurogenesis and initial synapse formation in the forebrain of the Ts65Dn mouse model of Down syndrome. *J. Neurosci.* **27**, 11483-11495.

Charlier, C., Singh, N. A., Ryan, S. G., Lewis, T. B., Reus, B. E., Leach, R. J. and Leppert, M. (1998). A pore mutation in a novel KQT-like potassium channel gene in an idiopathic epilepsy family. *Nat. Genet.* **18**, 53-55.

Chen, Y., Lu, J., Pan, H., Zhang, Y., Wu, H., Xu, K., Liu, X., Jiang, Y., Bao, X., Yao, Z., et al. (2003). Association between genetic variation of CACNA1H and childhood absence epilepsy. *Ann. Neurol.* **54**, 239-243.

Chen, C.-H., Huang, C.-C., Cheng, M.-C., Chiu, Y.-N., Tsai, W.-C., Wu, Y.-Y., Liu, S.-K. and Gau, S. S.-F. (2014). Genetic analysis of GABRB3 as a candidate gene of autism spectrum disorders. *Mol. Autism* **5**, 36.

Chen, W., Tankovic, A., Burger, P. B., Kusumoto, H., Traynelis, S. F. and Yuan, H. (2017). Functional Evaluation of a De Novo GRIN2A Mutation Identified in a Patient with Profound Global Developmental Delay and Refractory Epilepsy. *Mol. Pharmacol.* **91**, 317-330.

Chiu, C.-S., Brickley, S., Jensen, K., Southwell, A., Mckinney, S., Cull-Candy, S., Mody, I. and Lester, H. A. (2005). GABA transporter deficiency causes tremor, ataxia, nervousness, and increased GABA-induced tonic conductance in cerebellum. *J. Neurosci.* **25**, 3234-3245.

Claes, L., Del-Favero, J., Ceulemans, B., Lagae, L., Van Broeckhoven, C. and De Jonghe, P. (2001). De novo mutations in the sodium-channel gene SCN1A cause severe myoclonic epilepsy of infancy. *Am. J. Hum. Genet.* **68**, 1327-1332.

Cope, D. W., Di Giovanni, G., Fyson, S. J., Orbán, G., Errington, A. C., Lorincz, M. L., Gould, T. M., Carter, D. A. and Crunelli, V. (2009). Enhanced tonic GABAA inhibition in typical absence epilepsy. *Nat. Med.* **15**, 1392-1398.

Coupry, I., Roudaut, C., Stef, M., Delrue, M.-A., Marche, M., Burgelin, I., Taine, L., Cruaud, C., Lacombe, D. and Arveiler, B. (2002). Molecular analysis of the CBP gene in 60 patients with Rubinstein-Taybi syndrome. *J. Med. Genet.* **39**, 415-421.

Courcet, J.-B., Faivre, L., Malzac, P., Masurel-Paulet, A., Lopez, E., Callier, P., Lambert, L., Lemesle, M., Thevenon, J., Gigot, N., et al. (2012). The DYRK1A gene is a cause of syndromic intellectual disability with severe microcephaly and epilepsy. *J. Med. Genet.* **49**, 731-736.

Development • Supplementary information

Cross-Disorder Group of the Psychiatric Genomics Consortium (2013). Identification of risk loci with shared effects on five major psychiatric disorders: a genome-wide analysis. *Lancet* **381**, 1371-1379.

Damaj, L., Lupien-Meilleur, A., Lortie, A., Riou, É., Ospina, L. H., Gagnon, L., Vanasse, C. and Rossignol, E. (2015). CACNA1A haploinsufficiency causes cognitive impairment, autism and epileptic encephalopathy with mild cerebellar symptoms. *Eur. J. Hum. Genet.* **23**, 1505-1512.

Dang, T., Duan, W. Y., Yu, B., Tong, D. L., Cheng, C., Zhang, Y. F., Wu, W., Ye, K., Zhang, W. X., Wu, M., et al. (2018). Autism-associated Dyrk1a truncation mutants impair neuronal dendritic and spine growth and interfere with postnatal cortical development. *Mol. Psychiatry* **23**, 747-758.

Davies, M. N., Verdi, S., Burri, A., Trzaskowski, M., Lee, M., Hettema, J. M., Jansen, R., Boomsma, D. I. and Spector, T. D. (2015). Generalised Anxiety Disorder--A Twin Study of Genetic Architecture, Genome-Wide Association and Differential Gene Expression. *PLoS One* **10**, e0134865.

Davis, L. K., Maltman, N., Mosconi, M. W., Macmillan, C., Schmitt, L., Moore, K., Francis, S. M., Jacob, S., Sweeney, J. A. and Cook, E. H. (2012). Rare inherited A2BP1 deletion in a proband with autism and developmental hemiparesis. *Am. J. Med. Genet. A* **158A**, 1654-1661.

de Kovel, C. G. F., Meisler, M. H., Brilstra, E. H., van Berkestijn, F. M. C., van 't Slot, R., van Lieshout, S., Nijman, I. J., O'Brien, J. E., Hammer, M. F., Estacion, M., et al. (2014). Characterization of a de novo SCN8A mutation in a patient with epileptic encephalopathy. *Epilepsy Res.* **108**, 1511-1518.

de Kovel, C. G. F., Syrbe, S., Brilstra, E. H., Verbeek, N., Kerr, B., Dubbs, H., Bayat, A., Desai, S., Naidu, S., Srivastava, S., et al. (2017). Neurodevelopmental Disorders Caused by De Novo Variants in KCNB1 Genotypes and Phenotypes. *JAMA Neurol.* **74**, 1228-1236.

De Marco García, N. V., Priya, R., Tuncdemir, S. N., Fishell, G. and Karayannis, T. (2015). Sensory inputs control the integration of neurogliaform interneurons into cortical circuits. *Nat. Neurosci.* **18**, 393-401.

De Rubeis, S., He, X., Goldberg, A. P., Poultney, C. S., Samocha, K., Cicek, A. E., Kou, Y., Liu, L., Fromer, M., Walker, S., et al. (2014). Synaptic, transcriptional and chromatin genes disrupted in autism. *Nature* **515**, 209-215.

Deciphering Developmental Disorders Study (2015). Large-scale discovery of novel genetic causes of developmental disorders. *Nature* **519**, 223-228.

Deciphering Developmental Disorders Study (2017). Prevalence and architecture of de novo mutations in developmental disorders. *Nature* **542**, 433-438.

Development • Supplementary information

Delahanty, R. J., Zhang, Y., Bichell, T. J., Shen, W., Verdier, K., Macdonald, R. L., Xu, L., Boyd, K., Williams, J. and Kang, J.-Q. (2016). Beyond Epilepsy and Autism: Disruption of GABRB3 Causes Ocular Hypopigmentation. *Cell Rep.* **17**, 3115-3124.

Despang, P., Salamon, S., Breitenkamp, A. F., Kuzmenkina, E., Herzig, S. and Matthes, J. (2020). Autism-associated mutations in the CaVβ2 calcium-channel subunit increase Ba2+-currents and lead to differential modulation by the RGK-protein Gem. *Neurobiol. Dis.* **136**, 104721.

Díez-Sampedro, A., Silverman, W. R., Bautista, J. F. and Richerson, G. B. (2006). Mechanism of increased open probability by a mutation of the BK channel. *J. Neurophysiol.* **96**, 1507-1516.

Djukic, B., Casper, K. B., Philpot, B. D., Chin, L.-S. and McCarthy, K. D. (2007). Conditional Knock-Out of Kir4.1 Leads to Glial Membrane Depolarization, Inhibition of Potassium and Glutamate Uptake, and Enhanced Short-Term Synaptic Potentiation. *J. Neurosci.* **27**, 11354-11365.

Docherty, A. R., Moscati, A. A. and Fanous, A. H. (2016). Cross-Disorder Psychiatric Genomics. *Curr Behav Neurosci Rep* **3**, 256-263.

Du, W., Bautista, J. F., Yang, H., Diez-Sampedro, A., You, S.-A., Wang, L., Kotagal, P., Lüders, H. O., Shi, J., Cui, J., et al. (2005). Calcium-sensitive potassium channelopathy in human epilepsy and paroxysmal movement disorder. *Nat. Genet.* **37**, 733-738.

Echevarria-Cooper, D. M., Hawkins, N. A., Misra, S. N., Huffman, A. M., Thaxton, T., Thompson, C. H., Ben-Shalom, R., Nelson, A. D., Lipkin, A. M., George, A. L., et al. (2022). Cellular and behavioral effects of altered NaV1.2 sodium channel ion permeability in Scn2aK1422E mice. *Hum. Mol. Genet.*

El Achkar, C. M., Harrer, M., Smith, L., Kelly, M., Iqbal, S., Maljevic, S., Niturad, C. E., Vissers, L. E. L. M., Poduri, A., Yang, E., et al. (2021). Characterization of the GABRB2-associated neurodevelopmental disorders. *Ann. Neurol.* **89**, 573-586.

Endele, S., Rosenberger, G., Geider, K., Popp, B., Tamer, C., Stefanova, I., Milh, M., Kortüm, F., Fritsch, A., Pientka, F. K., et al. (2010). Mutations in GRIN2A and GRIN2B encoding regulatory subunits of NMDA receptors cause variable neurodevelopmental phenotypes. *Nat. Genet.* **42**, 1021-1026.

Epi4K Consortium (2016). De Novo Mutations in SLC1A2 and CACNA1A Are Important Causes of Epileptic Encephalopathies. *Am. J. Hum. Genet.* **99**, 287-298.

Development • Supplementary information

Epi4K Consortium, Epilepsy Phenome/Genome Project, Allen, A. S., Berkovic, S. F., Cossette, P., Delanty, N., Dlugos, D., Eichler, E. E., Epstein, M. P., Glauser, T., et al. (2013). De novo mutations in epileptic encephalopathies. *Nature* **501**, 217-221.

Escayg, A. and Goldin, A. L. (2010). Sodium channel SCN1A and epilepsy: mutations and mechanisms. *Epilepsia* **51**, 1650-1658.

Escayg, A., MacDonald, B. T., Meisler, M. H., Baulac, S., Huberfeld, G., An-Gourfinkel, I., Brice, A., LeGuern, E., Moulard, B., Chaigne, D., et al. (2000). Mutations of SCN1A, encoding a neuronal sodium channel, in two families with GEFS+2. *Nat. Genet.* **24**, 343-345.

Espinosa, J. S., Wheeler, D. G., Tsien, R. W. and Luo, L. (2009). Uncoupling dendrite growth and patterning: single-cell knockout analysis of NMDA receptor 2B. *Neuron* **62**, 205-217.

Estacion, M., O'Brien, J. E., Conravey, A., Hammer, M. F., Waxman, S. G., Dib-Hajj, S. D. and Meisler, M. H. (2014). A novel de novo mutation of SCN8A (Nav1.6) with enhanced channel activation in a child with epileptic encephalopathy. *Neurobiol. Dis.* **69**, 117-123.

Evers, J. M. G., Laskowski, R. A., Bertolli, M., Clayton-Smith, J., Deshpande, C., Eason, J., Elmslie, F., Flinter, F., Gardiner, C., Hurst, J. A., et al. (2017). Structural analysis of pathogenic mutations in the DYRK1A gene in patients with developmental disorders. *Hum. Mol. Genet.* **26**, 519-526.

Fakira, A. K., Gaspers, L. D., Thomas, A. P., Li, H., Jain, M. R. and Elkabes, S. (2012). Purkinje cell dysfunction and delayed death in plasma membrane calcium ATPase 2-heterozygous mice. *Mol. Cell. Neurosci.* **51**, 22-31.

Fedele, L., Newcombe, J., Topf, M., Gibb, A., Harvey, R. J. and Smart, T. G. (2018). Disease-associated missense mutations in GluN2B subunit alter NMDA receptor ligand binding and ion channel properties. *Nat. Commun.* **9**, 1-15.

Ferraro, T. N., Golden, G. T., Smith, G. G., Martin, J. F., Lohoff, F. W., Gieringer, T. A., Zamboni, D., Schwebel, C. L., Press, Danielle M., KratzerZhao, S. H. O., et al. (2004). Fine mapping of a seizure susceptibility locus on mouse Chromosome 1: nomination of Kcnj10 as a causative gene. *Mamm. Genome* **15**, 239-251.

Ferreira, M. A. R., O'Donovan, M. C., Meng, Y. A., Jones, I. R., Ruderfer, D. M., Jones, L., Fan, J., Kirov, G., Perlis, R. H., Green, E. K., et al. (2008). Collaborative genome-wide association analysis supports a role for ANK3 and CACNA1C in bipolar disorder. *Nat. Genet.* **40**, 1056-1058.

Ferri, S. L., Pallathra, A. A., Kim, H., Dow, H. C., Raje, P., McMullen, M., Bilker, W. B., Siegel, S. J., Abel, T. and Brodkin, E. S. (2020). Sociability development in mice with cell-specific deletion of the NMDA receptor NR1 subunit gene. *Genes Brain Behav.* **19**, e12624.

Development • Supplementary information

Filosa, J. A., Bonev, A. D., Straub, S. V., Meredith, A. L., Wilkerson, M. K., Aldrich, R. W. and Nelson, M. T. (2006). Local potassium signaling couples neuronal activity to vasodilation in the brain. *Nat. Neurosci.* **9**, 1397-1403.

Firth, H. V., Wright, C. F. and DDD Study (2011). The Deciphering Developmental Disorders (DDD) study. *Dev. Med. Child Neurol.* **53**, 702-703.

Flavell, S. W., Kim, T.-K., Gray, J. M., Harmin, D. A., Hemberg, M., Hong, E. J., Markenscoff-Papadimitriou, E., Bear, D. M. and Greenberg, M. E. (2008). Genome-wide analysis of MEF2 transcriptional program reveals synaptic target genes and neuronal activity-dependent polyadenylation site selection. *Neuron* **60**, 1022-1038.

Forrest, D., Yuzaki, M., Soares, H. D., Ng, L., Luk, D. C., Sheng, M., Stewart, C. L., Morgan, J. I., Connor, J. A. and Curran, T. (1994). Targeted disruption of NMDA receptor 1 gene abolishes NMDA response and results in neonatal death. *Neuron* **13**, 325-338.

Fotaki, V., Dierssen, M., Alcántara, S., Martínez, S., Martí, E., Casas, C., Visa, J., Soriano, E., Estivill, X. and Arbonés, M. L. (2002). Dyrk1A haploinsufficiency affects viability and causes developmental delay and abnormal brain morphology in mice. *Mol. Cell. Biol.* **22**, 6636-6647.

Fotaki, V., Martínez De Lagrán, M., Estivill, X., Arbonés, M. and Dierssen, M. (2004). Haploinsufficiency of Dyrk1A in mice leads to specific alterations in the development and regulation of motor activity. *Behav. Neurosci.* **118**, 815-821.

Freund, R. K., Graw, S., Choo, K. S., Stevens, K. E., Leonard, S. and Dell'Acqua, M. L. (2016). Genetic knockout of the α7 nicotinic acetylcholine receptor gene alters hippocampal long-term potentiation in a background strain-dependent manner. *Neurosci. Lett.* **627**, 1-6.

Freunscht, I., Popp, B., Blank, R., Endele, S., Moog, U., Petri, H., Prott, E.-C., Reis, A., Rübo, J., Zabel, B., et al. (2013). Behavioral phenotype in five individuals with de novo mutations within the GRIN2B gene. *Behav. Brain Funct.* **9**, 20.

Fromer, M., Pocklington, A. J., Kavanagh, D. H., Williams, H. J., Dwyer, S., Gormley, P., Georgieva, L., Rees, E., Palta, P., Ruderfer, D. M., et al. (2014). De novo mutations in schizophrenia implicate synaptic networks. *Nature* **506**, 179-184.

Fry, A. E., Fawcett, K. A., Zelnik, N., Yuan, H., Thompson, B. A. N., Shemer-Meiri, L., Cushion, T. D., Mugalaasi, H., Sims, D., Stoodley, N., et al. (2018). De novo mutations in GRIN1 cause extensive bilateral polymicrogyria. *Brain* **141**, 698-712.

Gackière, F., Bidaux, G., Delcourt, P., Van Coppenolle, F., Katsogiannou, M., Dewailly, E., Bavencoffe, A., Van Chuoï-Mariot, M. T., Mauroy, B., Prevarskaya, N., et al. (2008). CaV3.2 T-type calcium channels are involved in calcium-dependent secretion of neuroendocrine prostate cancer cells. *J. Biol. Chem.* **283**, 10162-10173.

Gandal, M. J., Sisti, J., Klook, K., Ortinski, P. I., Leitman, V., Liang, Y., Thieu, T., Anderson, R., Pierce, R. C., Jonak, G., et al. (2012). GABAB-mediated rescue of altered excitatory-inhibitory balance, gamma synchrony and behavioral deficits following constitutive NMDAR-hypofunction. *Transl. Psychiatry* **2**, e142.

Gandal, M. J., Zhang, P., Hadjimichael, E., Walker, R. L., Chen, C., Liu, S., Won, H., van Bakel, H., Varghese, M., Wang, Y., et al. (2018). Transcriptome-wide isoform-level dysregulation in ASD, schizophrenia, and bipolar disorder. *Science* **362**,.

Gangarossa, G., Laffray, S., Bourinet, E. and Valjent, E. (2014). T-type calcium channel Cav3.2 deficient mice show elevated anxiety, impaired memory and reduced sensitivity to psychostimulants. *Front. Behav. Neurosci.* **8**, 92.

Gao, K., Tankovic, A., Zhang, Y., Kusumoto, H., Zhang, J., Chen, W., XiangWei, W., Shaulsky, G. H., Hu, C., Traynelis, S. F., et al. (2017). A de novo loss-of-function GRIN2A mutation associated with childhood focal epilepsy and acquired epileptic aphasia. *PLoS One* **12**, e0170818.

Gault, J., Hopkins, J., Berger, R., Drebing, C., Logel, J., Walton, C., Short, M., Vianzon, R., Olincy, A., Ross, R. G., et al. (2003). Comparison of polymorphisms in the alpha7 nicotinic receptor gene and its partial duplication in schizophrenic and control subjects. *Am. J. Med. Genet. B Neuropsychiatr. Genet.* **123B**, 39-49.

Gehman, L. T., Stoilov, P., Maguire, J., Damianov, A., Lin, C.-H., Shiue, L., Ares, M., Jr, Mody, I. and Black, D. L. (2011). The splicing regulator Rbfox1 (A2BP1) controls neuronal excitation in the mammalian brain. *Nat. Genet.* **43**, 706-711.

Gillentine, M. A., Yin, J., Bajic, A., Zhang, P., Cummock, S., Kim, J. J. and Schaaf, C. P. (2017). Functional Consequences of CHRNA7 Copy-Number Alterations in Induced Pluripotent Stem Cells and Neural Progenitor Cells. *Am. J. Hum. Genet.* **101**, 874-887.

Gilling, M., Rasmussen, H. B., Calloe, K., Sequeira, A. F., Baretto, M., Oliveira, G., Almeida, J., Lauritsen, M. B., Ullmann, R., Boonen, S. E., et al. (2013). Dysfunction of the Heteromeric KV7.3/KV7.5 Potassium Channel is Associated with Autism Spectrum Disorders. *Front. Genet.* **4**, 54.

Girirajan, S., Dennis, M. Y., Baker, C., Malig, M., Coe, B. P., Campbell, C. D., Mark, K., Vu, T. H., Alkan, C., Cheng, Z., et al. (2013). Refinement and discovery of new hotspots of copy-number variation associated with autism spectrum disorder. *Am. J. Hum. Genet.* **92**, 221-237.

Graziano, C., Despang, P., Palombo, F., Severi, G., Posar, A., Cassio, A., Pippucci, T., Isidori, F., Matthes, J. and Bonora, E. (2021). A New Homozygous CACNB2 Mutation has Functional Relevance and Supports a Role for Calcium Channels in Autism Spectrum Disorder. *J. Autism Dev. Disord.* **51**, 377-381.

Green, E. K., Grozeva, D., Jones, I., Jones, L., Kirov, G., Caesar, S., Gordon-Smith, K., Fraser, C., Forty, L., Russell, E., et al. (2010). The bipolar disorder risk allele at CACNA1C also confers risk of recurrent major depression and of schizophrenia. *Mol. Psychiatry* **15**, 1016-1022.

Green, E. K., Hamshere, M., Forty, L., Gordon-Smith, K., Fraser, C., Russell, E., Grozeva, D., Kirov, G., Holmans, P., Moran, J. L., et al. (2013). Replication of bipolar disorder susceptibility alleles and identification of two novel genome-wide significant associations in a new bipolar disorder case-control sample. *Mol. Psychiatry* **18**, 1302-1307.

Griesi-Oliveira, K., Acab, A., Gupta, A. R., Sunaga, D. Y., Chailangkarn, T., Nicol, X., Nunez, Y., Walker, M. F., Murdoch, J. D., Sanders, S. J., et al. (2015). Modeling non-syndromic autism and the impact of TRPC6 disruption in human neurons. *Mol. Psychiatry* **20**, 1350-1365.

Grinton, B. E., Heron, S. E., Pelekanos, J. T., Zuberi, S. M., Kivity, S., Afawi, Z., Williams, T. C., Casalaz, D. M., Yendle, S., Linder, I., et al. (2015). Familial neonatal seizures in 36 families: Clinical and genetic features correlate with outcome. *Epilepsia* **56**, 1071-1080.

Guimerá, J., Casas, C., Pucharcòs, C., Solans, A., Domènech, A., Planas, A. M., Ashley, J., Lovett, M., Estivill, X. and Pritchard, M. A. (1996). A human homologue of Drosophila minibrain (MNB) is expressed in the neuronal regions affected in Down syndrome and maps to the critical region. *Hum. Mol. Genet.* **5**, 1305-1310.

Gulsuner, S., Stein, D. J., Susser, E. S., Sibeko, G., Pretorius, A., Walsh, T., Majara, L., Mndini, M. M., Mqulwana, S. G., Ntola, O. A., et al. (2020). Genetics of schizophrenia in the South African Xhosa. *Science* **367**, 569-573.

Gutzmann, J. J., Lin, L. and Hoffman, D. A. (2019). Functional Coupling of Cav2.3 and BK Potassium Channels Regulates Action Potential Repolarization and Short-Term Plasticity in the Mouse Hippocampus. *Front. Cell. Neurosci.* **13**, 27.

Hackmann, K., Matko, S., Gerlach, E.-M., von der Hagen, M., Klink, B., Schrock, E., Rump, A. and Di Donato, N. (2013). Partial deletion of GLRB and GRIA2 in a patient with intellectual disability. *Eur. J. Hum. Genet.* **21**, 112-114.

Hamada, N., Ito, H., Nishijo, T., Iwamoto, I., Morishita, R., Tabata, H., Momiyama, T. and Nagata, K.-I. (2016). Essential role of the nuclear isoform of RBFOX1, a candidate gene for autism spectrum disorders, in the brain development. *Sci. Rep.* **6**, 30805.

Development • Supplementary information

Hamdan, F. F., Gauthier, J., Araki, Y., Lin, D.-T., Yoshizawa, Y., Higashi, K., Park, A.-R., Spiegelman, D., Dobrzeniecka, S., Piton, A., et al. (2011). Excess of de novo deleterious mutations in genes associated with glutamatergic systems in nonsyndromic intellectual disability. *Am. J. Hum. Genet.* **88**, 306-316.

Hamdan, F. F., Myers, C. T., Cossette, P., Lemay, P., Spiegelman, D., Laporte, A. D., Nassif, C., Diallo, O., Monlong, J., Cadieux-Dion, M., et al. (2017). High Rate of Recurrent De Novo Mutations in Developmental and Epileptic Encephalopathies. *Am. J. Hum. Genet.* **101**, 664-685.

Hämmerle, B., Ulin, E., Guimera, J., Becker, W., Guillemot, F. and Tejedor, F. J. (2011). Transient expression of Mnb/Dyrk1a couples cell cycle exit and differentiation of neuronal precursors by inducing p27KIP1 expression and suppressing NOTCH signaling. *Development* **138**, 2543-2554.

Harrington, A. J., Raissi, A., Rajkovich, K., Berto, S., Kumar, J., Molinaro, G., Raduazzo, J., Guo, Y., Loerwald, K., Konopka, G., et al. (2016). MEF2C regulates cortical inhibitory and excitatory synapses and behaviors relevant to neurodevelopmental disorders. *Elife* **5**,.

Heinemann, S. H., Terlau, H., Stühmer, W., Imoto, K. and Numa, S. (1992). Calcium channel characteristics conferred on the sodium channel by single mutations. *Nature* **356**, 441-443.

Helbig, K. L., Lauerer, R. J., Bahr, J. C., Souza, I. A., Myers, C. T., Uysal, B., Schwarz, N., Gandini, M. A., Huang, S., Keren, B., et al. (2018). De Novo Pathogenic Variants in CACNA1E Cause Developmental and Epileptic Encephalopathy with Contractures, Macrocephaly, and Dyskinesias. *Am. J. Hum. Genet.* **103**, 666-678.

Heron, S. E., Khosravani, H., Varela, D., Bladen, C., Williams, T. C., Newman, M. R., Scheffer, I. E., Berkovic, S. F., Mulley, J. C. and Zamponi, G. W. (2007). Extended spectrum of idiopathic generalized epilepsies associated with CACNA1H functional variants. *Ann. Neurol.* **62**, 560-568.

Hirose, S., Zenri, F., Akiyoshi, H., Fukuma, G., Iwata, H., Inoue, T., Yonetani, M., Tsutsumi, M., Muranaka, H., Kurokawa, T., et al. (2000). A novel mutation ofKCNQ3 (c.925T?C) in a Japanese family with benign familial neonatal convulsions. *Ann. Neurol.* **47**, 822-826.

Hofer, N. T., Tuluc, P., Ortner, N. J., Nikonishyna, Y. V., Fernándes-Quintero, M. L., Liedl, K. R., Flucher, B. E., Cox, H. and Striessnig, J. (2020). Biophysical classification of a CACNA1D de novo mutation as a high-risk mutation for a severe neurodevelopmental disorder. *Mol. Autism* **11**, 4.

Development • Supplementary information

Hood, R. L., Lines, M. A., Nikkel, S. M., Schwartzentruber, J., Beaulieu, C., Nowaczyk, M. J. M., Allanson, J., Kim, C. A., Wieczorek, D., Moilanen, J. S., et al. (2012). Mutations in SRCAP, encoding SNF2-related CREBBP activator protein, cause Floating-Harbor syndrome. *Am. J. Hum. Genet.* **90**, 308-313.

Hoppman-Chaney, N., Wain, K., Seger, P. R., Superneau, D. W. and Hodge, J. C. (2013). Identification of single gene deletions at 15q13.3: further evidence that CHRNA7 causes the 15q13.3 microdeletion syndrome phenotype. *Clin. Genet.* **83**, 345-351.

Hori, T., Ikuta, S., Hattori, S., Takao, K., Miyakawa, T. and Koike, C. (2021). Mice with mutations in Trpm1, a gene in the locus of 15q13.3 microdeletion syndrome, display pronounced hyperactivity and decreased anxiety-like behavior. *Mol. Brain* **14**, 61.

Hu, C., Depuy, S. D., Yao, J., McIntire, W. E. and Barrett, P. Q. (2009). Protein kinase A activity controls the regulation of T-type CaV3.2 channels by Gbetagamma dimers. *J. Biol. Chem.* **284**, 7465-7473.

Imbrici, P., Jaffe, S. L., Eunson, L. H., Davies, N. P., Herd, C., Robertson, R., Kullmann, D. M. and Hanna, M. G. (2004). Dysfunction of the brain calcium channel CaV2.1 in absence epilepsy and episodic ataxia. *Brain* **127**, 2682-2692.

Iossifov, I., Ronemus, M., Levy, D., Wang, Z., Hakker, I., Rosenbaum, J., Yamrom, B., Lee, Y.-H., Narzisi, G., Leotta, A., et al. (2012). De novo gene disruptions in children on the autistic spectrum. *Neuron* **74**, 285-299.

Iossifov, I., O'Roak, B. J., Sanders, S. J., Ronemus, M., Krumm, N., Levy, D., Stessman, H. A., Witherspoon, K. T., Vives, L., Patterson, K. E., et al. (2014). The contribution of de novo coding mutations to autism spectrum disorder. *Nature* **515**, 216-221.

Iossifov, I., Levy, D., Allen, J., Ye, K., Ronemus, M., Lee, Y.-H., Yamrom, B. and Wigler, M. (2015). Low load for disruptive mutations in autism genes and their biased transmission. *Proceedings of the National Academy of Sciences* **112**, E5600-E5607.

Jensen, K., Chiu, C.-S., Sokolova, I., Lester, H. A. and Mody, I. (2003). GABA transporter-1 (GAT1)-deficient mice: differential tonic activation of GABAA versus GABAB receptors in the hippocampus. *J. Neurophysiol.* **90**, 2690-2701.

Ji, J., Lee, H., Argiropoulos, B., Dorrani, N., Mann, J., Martinez-Agosto, J. A., Gomez-Ospina, N., Gallant, N., Bernstein, J. A., Hudgins, L., et al. (2015). DYRK1A haploinsufficiency causes a new recognizable syndrome with microcephaly, intellectual disability, speech impairment, and distinct facies. *Eur. J. Hum. Genet.* **23**, 1473-1481.

Development • Supplementary information

Jia, Z., Agopyan, N., Miu, P., Xiong, Z., Henderson, J., Gerlai, R., Taverna, F. A., Velumian, A., MacDonald, J., Carlen, P., et al. (1996). Enhanced LTP in mice deficient in the AMPA receptor GluR2. *Neuron* **17**, 945-956.

Jiang, H., Jiang, W., Zou, J., Wang, B., Yu, M., Pan, Y., Lin, Y., Mao, Y. and Wang, Y. (2015a). The GluN2B subunit of N-methy-D-asparate receptor regulates the radial migration of cortical neurons in vivo. *Brain Res.* **1610**, 20-32.

Jiang, R. (2015). Walking on multiple disease-gene networks to prioritize candidate genes. *J. Mol. Cell Biol.* **7**, 214-230.

Jiang, Y.-H., Yuen, R. K. C., Jin, X., Wang, M., Chen, N., Wu, X., Ju, J., Mei, J., Shi, Y., He, M., et al. (2013). Detection of clinically relevant genetic variants in autism spectrum disorder by whole-genome sequencing. *Am. J. Hum. Genet.* **93**, 249-263.

Jiang, Y., Han, Y., Petrovski, S., Owzar, K., Goldstein, D. B. and Allen, A. S. (2015b). Incorporating Functional Information in Tests of Excess De Novo Mutational Load. *Am. J. Hum. Genet.* **97**, 272-283.

Jiang, X., Lachance, M. and Rossignol, E. (2016). Involvement of cortical fast-spiking parvalbumin-positive basket cells in epilepsy. *Prog. Brain Res.* **226**, 81-126.

Jiang, X., Raju, P. K., D'Avanzo, N., Lachance, M., Pepin, J., Dubeau, F., Mitchell, W. G., Bello-Espinosa, L. E., Pierson, T. M., Minassian, B. A., et al. (2019). Both gain-of-function and loss-of-function de novo CACNA1A mutations cause severe developmental epileptic encephalopathies in the spectrum of Lennox-Gastaut syndrome. *Epilepsia* **60**, 1881-1894.

Jouvenceau, A., Eunson, L. H., Spauschus, A., Ramesh, V., Zuberi, S. M., Kullmann, D. M. and Hanna, M. G. (2001). Human epilepsy associated with dysfunction of the brain P/Q-type calcium channel. *Lancet* **358**, 801-807.

Judy, J. T., Seifuddin, F., Pirooznia, M., Mahon, P. B., Bipolar Genome Study Consortium, Jancic, D., Goes, F. S., Schulze, T., Cichon, S., Noethen, M., et al. (2013). Converging Evidence for Epistasis between ANK3 and Potassium Channel Gene KCNQ2 in Bipolar Disorder. *Front. Genet.* **4**, 87.

Kaja, S., van de Ven, R. C. G., Broos, L. A. M., Veldman, H., van Dijk, J. G., Verschuuren, J. J. G. M., Frants, R. R., Ferrari, M. D., van den Maagdenberg, A. M. J. M. and Plomp, J. J. (2005). Gene dosage-dependent transmitter release changes at neuromuscular synapses of CACNA1A R192Q knockin mice are non-progressive and do not lead to morphological changes or muscle weakness. *Neuroscience* **135**, 81-95.

Development • Supplementary information

Kamijo, S., Ishii, Y., Horigane, S.-I., Suzuki, K., Ohkura, M., Nakai, J., Fujii, H., Takemoto-Kimura, S. and Bito, H. (2018). A Critical Neurodevelopmental Role for L-Type Voltage-Gated Calcium Channels in Neurite Extension and Radial Migration. *J. Neurosci.* **38**, 5551-5566.

Kannangara, T. S., Eadie, B. D., Bostrom, C. A., Morch, K., Brocardo, P. S. and Christie, B. R. (2014). GluN2A−/− Mice Lack Bidirectional Synaptic Plasticity in the Dentate Gyrus and Perform Poorly on Spatial Pattern Separation Tasks. *Cereb. Cortex* **25**, 2102-2113.

Kellner, S., Abbasi, A., Carmi, I., Heinrich, R., Garin-Shkolnik, T., Hershkovitz, T., Giladi, M., Haitin, Y., Johannesen, K. M., Steensbjerre Møller, R., et al. (2021). Two de novo GluN2B mutations affect multiple NMDAR-functions and instigate severe pediatric encephalopathy. *Elife* **10**,.

Khosravani, H., Altier, C., Simms, B., Hamming, K. S., Snutch, T. P., Mezeyova, J., McRory, J. E. and Zamponi, G. W. (2004). Gating effects of mutations in the Cav3.2 T-type calcium channel associated with childhood absence epilepsy. *J. Biol. Chem.* **279**, 9681-9684.

Kim, J., Ghosh, S., Liu, H., Tateyama, M., Kass, R. S. and Pitt, G. S. (2004). Calmodulin mediates Ca2+ sensitivity of sodium channels. *J. Biol. Chem.* **279**, 45004-45012.

Kiyama, Y., Manabe, T., Sakimura, K., Kawakami, F., Mori, H. and Mishina, M. (1998). Increased thresholds for long-term potentiation and contextual learning in mice lacking the NMDA-type glutamate receptor epsilon1 subunit. *J. Neurosci.* **18**, 6704-6712.

Klassen, T., Davis, C., Goldman, A., Burgess, D., Chen, T., Wheeler, D., McPherson, J., Bourquin, T., Lewis, L., Villasana, D., et al. (2011). Exome sequencing of ion channel genes reveals complex profiles confounding personal risk assessment in epilepsy. *Cell* **145**, 1036-1048.

Kline, C. F., Scott, J., Curran, J., Hund, T. J. and Mohler, P. J. (2014). Ankyrin-B regulates Cav2.1 and Cav2.2 channel expression and targeting. *J. Biol. Chem.* **289**, 5285-5295.

Kluckova, D., Kolnikova, M., Lacinova, L., Jurkovicova-Tarabova, B., Foltan, T., Demko, V., Kadasi, L., Ficek, A. and Soltysova, A. (2020). A study among the genotype, functional alternations, and phenotype of 9 SCN1A mutations in epilepsy patients. *Sci. Rep.* **10**, 10288.

Kozel, P. J., Friedman, R. A., Erway, L. C., Yamoah, E. N., Liu, L. H., Riddle, T., Duffy, J. J., Doetschman, T., Miller, M. L., Cardell, E. L., et al. (1998). Balance and hearing deficits in mice with a null mutation in the gene encoding plasma membrane Ca2+-ATPase isoform 2. *J. Biol. Chem.* **273**, 18693-18696.

Krey, J. F., Paşca, S. P., Shcheglovitov, A., Yazawa, M., Schwemberger, R., Rasmusson, R. and Dolmetsch, R. E. (2013). Timothy syndrome is associated with activity-dependent dendritic retraction in rodent and human neurons. *Nat. Neurosci.* **16**, 201-209.

Kurabayashi, N. and Sanada, K. (2013). Increased dosage of DYRK1A and DSCR1 delays neuronal differentiation in neocortical progenitor cells. *Genes Dev.* **27**, 2708-2721.

Landmann, J., Richter, F., Oros-Peusquens, A.-M., Shah, N. J., Classen, J., Neely, G. G., Richter, A., Penninger, J. M. and Bechmann, I. (2018). Neuroanatomy of pain-deficiency and cross-modal activation in calcium channel subunit (CACN) α2δ3 knockout mice. *Brain Struct. Funct.* **223**, 111-130.

Larson, V. A., Mironova, Y., Vanderpool, K. G., Waisman, A., Rash, J. E., Agarwal, A. and Bergles, D. E. (2018). Oligodendrocytes control potassium accumulation in white matter and seizure susceptibility. *Elife* **7**,.

Laumonnier, F., Roger, S., Guérin, P., Molinari, F., M'rad, R., Cahard, D., Belhadj, A., Halayem, M., Persico, A. M., Elia, M., et al. (2006). Association of a functional deficit of the BKCa channel, a synaptic regulator of neuronal excitability, with autism and mental retardation. *Am. J. Psychiatry* **163**, 1622-1629.

Lee, I.-C., Chang, T.-M., Liang, J.-S. and Li, S.-Y. (2019). KCNQ2 mutations in childhood nonlesional epilepsy: Variable phenotypes and a novel mutation in a case series. *Mol Genet Genomic Med* **7**, e00816.

Lee, K.-S., Choi, M., Kwon, D.-W., Kim, D., Choi, J.-M., Kim, A.-K., Ham, Y., Han, S.-B., Cho, S. and Cheon, C. K. (2020). A novel de novo heterozygous DYRK1A mutation causes complete loss of DYRK1A function and developmental delay. *Sci. Rep.* **10**, 9849.

Lemke, J. R. (2020). Predicting incidences of neurodevelopmental disorders. *Brain* **143**, 1046-1048.

Lemke, J. R., Lal, D., Reinthaler, E. M., Steiner, I., Nothnagel, M., Alber, M., Geider, K., Laube, B., Schwake, M., Finsterwalder, K., et al. (2013). Mutations in GRIN2A cause idiopathic focal epilepsy with rolandic spikes. *Nat. Genet.* **45**, 1067-1072.

Lemke, J. R., Hendrickx, R., Geider, K., Laube, B., Schwake, M., Harvey, R. J., James, V. M., Pepler, A., Steiner, I., Hörtnagel, K., et al. (2014). GRIN2B mutations in West syndrome and intellectual disability with focal epilepsy. *Ann. Neurol.* **75**, 147-154.

Lemke, J. R., Geider, K., Helbig, K. L., Heyne, H. O., Schütz, H., Hentschel, J., Courage, C., Depienne, C., Nava, C., Heron, D., et al. (2016). Delineating the GRIN1 phenotypic spectrum: A distinct genetic NMDA receptor encephalopathy. *Neurology* **86**, 2171-2178.

Leonard, S., Gault, J., Hopkins, J., Logel, J., Vianzon, R., Short, M., Drebing, C., Berger, R., Venn, D., Sirota, P., et al. (2002). Association of promoter variants in the α7 nicotinic acetylcholine receptor subunit gene with an inhibitory deficit found in schizophrenia. *Arch. Gen. Psychiatry* **59**, 1085-1096.

Levy, J. A., LaFlamme, C. W., Tsaprailis, G., Crynen, G. and Page, D. T. (2021). Dyrk1a Mutations Cause Undergrowth of Cortical Pyramidal Neurons via Dysregulated Growth Factor Signaling. *Biol. Psychiatry* **90**, 295-306.

Li, H., Radford, J. C., Ragusa, M. J., Shea, K. L., McKercher, S. R., Zaremba, J. D., Soussou, W., Nie, Z., Kang, Y.-J., Nakanishi, N., et al. (2008). Transcription factor MEF2C influences neural stem/progenitor cell differentiation and maturation in vivo. *Proc. Natl. Acad. Sci. U. S. A.* **105**, 9397-9402.

Liang, L., Li, X., Moutton, S., Schrier Vergano, S. A., Cogné, B., Saint-Martin, A., Hurst, A. C. E., Hu, Y., Bodamer, O., Thevenon, J., et al. (2019). De novo loss-of-function KCNMA1 variants are associated with a new multiple malformation syndrome and a broad spectrum of developmental and neurological phenotypes. *Hum. Mol. Genet.* **28**, 2937-2951.

Limpitikul, W. B., Dick, I. E., Ben-Johny, M. and Yue, D. T. (2016). An autism-associated mutation in CaV1.3 channels has opposing effects on voltage- and Ca(2+)-dependent regulation. *Sci. Rep.* **6**, 27235.

Liu, X.-R., Xu, X.-X., Lin, S.-M., Fan, C.-Y., Ye, T.-T., Tang, B., Shi, Y.-W., Su, T., Li, B.-M., Yi, Y.-H., et al. (2021). GRIN2A Variants Associated With Idiopathic Generalized Epilepsies. *Front. Mol. Neurosci.* **14**, 720984.

Llinás, R. R., Choi, S., Urbano, F. J. and Shin, H.-S. (2007). Gamma-band deficiency and abnormal thalamocortical activity in P/Q-type channel mutant mice. *Proc. Natl. Acad. Sci. U. S. A.* **104**, 17819-17824.

Lu, W., Shi, Y., Jackson, A. C., Bjorgan, K., During, M. J., Sprengel, R., Seeburg, P. H. and Nicoll, R. A. (2009). Subunit composition of synaptic AMPA receptors revealed by a single-cell genetic approach. *Neuron* **62**, 254-268.

Luo, F., Sclip, A., Jiang, M. and Südhof, T. C. (2020). Neurexins cluster Ca2+ channels within the presynaptic active zone. *EMBO J.* **39**, e103208.

Makinson, C. D., Tanaka, B. S., Sorokin, J. M., Wong, J. C., Christian, C. A., Goldin, A. L., Escayg, A. and Huguenard, J. R. (2017). Regulation of Thalamic and Cortical Network Synchrony by Scn8a. *Neuron* **93**, 1165-1179.e6.

Martin, C. L., Duvall, J. A., Ilkin, Y., Simon, J. S., Arreaza, M. G., Wilkes, K., Alvarez-Retuerto, A., Whichello, A., Powell, C. M., Rao, K., et al. (2007). Cytogenetic and molecular characterization of A2BP1/FOX1 as a candidate gene for autism. *Am. J. Med. Genet. B Neuropsychiatr. Genet.* **144B**, 869-876.

Marwick, K. F. M., Skehel, P. A., Hardingham, G. E. and Wyllie, D. J. A. (2019). The human NMDA receptor GluN2AN615K variant influences channel blocker potency. *Pharmacol Res Perspect* **7**, e00495.

Matsunami, N., Hensel, C. H., Baird, L., Stevens, J., Otterud, B., Leppert, T., Varvil, T., Hadley, D., Glessner, J. T., Pellegrino, R., et al. (2014). Identification of rare DNA sequence variants in high-risk autism families and their prevalence in a large case/control population. *Mol. Autism* **5**, 5.

Mayer, C., Hafemeister, C., Bandler, R. C., Machold, R., Batista Brito, R., Jaglin, X., Allaway, K., Butler, A., Fishell, G. and Satija, R. (2018). Developmental diversification of cortical inhibitory interneurons. *Nature* **555**, 457-462.

Mead, A. N. and Stephens, D. N. (2003). Involvement of AMPA Receptor GluR2 Subunits in Stimulus-Reward Learning: Evidence from Glutamate Receptor gria2 Knock-Out Mice. *J. Neurosci.* **23**, 9500-9507.

Meredith, A. L., Wiler, S. W., Miller, B. H., Takahashi, J. S., Fodor, A. A., Ruby, N. F. and Aldrich, R. W. (2006). BK calcium-activated potassium channels regulate circadian behavioral rhythms and pacemaker output. *Nat. Neurosci.* **9**, 1041-1049.

Merk, D. J., Ohli, J., Merk, N. D., Thatikonda, V., Morrissy, S., Schoof, M., Schmid, S. N., Harrison, L., Filser, S., Ahlfeld, J., et al. (2018). Opposing Effects of CREBBP Mutations Govern the Phenotype of Rubinstein-Taybi Syndrome and Adult SHH Medulloblastoma. *Dev. Cell* **44**, 709-724.e6.

Miceli, F., Soldovieri, M. V., Ambrosino, P., De Maria, M., Migliore, M., Migliore, R. and Taglialatela, M. (2015). Early-onset epileptic encephalopathy caused by gain-of-function mutations in the voltage sensor of Kv7.2 and Kv7.3 potassium channel subunits. *J. Neurosci.* **35**, 3782-3793.

Miceli, F., Carotenuto, L., Barrese, V., Soldovieri, M. V., Heinzen, E. L., Mandel, A. M., Lippa, N., Bier, L., Goldstein, D. B., Cooper, E. C., et al. (2020). A Novel Kv7.3 Variant in the Voltage-Sensing S4 Segment in a Family With Benign Neonatal Epilepsy: Functional Characterization and in vitro Rescue by β-Hydroxybutyrate. *Front. Physiol.* **11**, 1040.

Mikhail, F. M., Lose, E. J., Robin, N. H., Descartes, M. D., Rutledge, K. D., Rutledge, S. L., Korf, B. R. and Carroll, A. J. (2011). Clinically relevant single gene or intragenic deletions encompassing critical neurodevelopmental genes in patients with developmental delay, mental retardation, and/or autism spectrum disorders. *Am. J. Med. Genet. A* **155A**, 2386-2396.

Milh, M., Boutry-Kryza, N., Sutera-Sardo, J., Mignot, C., Auvin, S., Lacoste, C., Villeneuve, N., Roubertie, A., Heron, B., Carneiro, M., et al. (2013). Similar early characteristics but variable neurological outcome of patients with a de novo mutation of KCNQ2. *Orphanet J. Rare Dis.* **8**, 80.

Missler, M., Zhang, W., Rohlmann, A., Kattenstroth, G., Hammer, R. E., Gottmann, K. and Südhof, T. C. (2003). Alpha-neurexins couple Ca2+ channels to synaptic vesicle exocytosis. *Nature 423*, 939-948.

Møller, R. S., Kübart, S., Hoeltzenbein, M., Heye, B., Vogel, I., Hansen, C. P., Menzel, C., Ullmann, R., Tommerup, N., Ropers, H.-H., et al. (2008). Truncation of the Down syndrome candidate gene DYRK1A in two unrelated patients with microcephaly. *Am. J. Hum. Genet.* **82**, 1165-1170.

Development • Supplementary information

Mukherjee, K., Patel, P. A., Rajan, D. S., LaConte, L. E. W. and Srivastava, S. (2020). Survival of a male patient harboring CASK Arg27Ter mutation to adolescence. *Mol Genet Genomic Med* **8**, e1426.

Myers, R. A., Casals, F., Gauthier, J., Hamdan, F. F., Keebler, J., Boyko, A. R., Bustamante, C. D., Piton, A. M., Spiegelman, D., Henrion, E., et al. (2011). A population genetic approach to mapping neurological disorder genes using deep resequencing. *PLoS Genet.* **7**, e1001318.

Najas, S., Arranz, J., Lochhead, P. A., Ashford, A. L., Oxley, D., Delabar, J. M., Cook, S. J., Barallobre, M. J. and Arbonés, M. L. (2015). DYRK1A-mediated Cyclin D1 Degradation in Neural Stem Cells Contributes to the Neurogenic Cortical Defects in Down Syndrome. *EBioMedicine* **2**, 120-134.

Najm, J., Horn, D., Wimplinger, I., Golden, J. A., Chizhikov, V. V., Sudi, J., Christian, S. L., Ullmann, R., Kuechler, A., Haas, C. A., et al. (2008). Mutations of CASK cause an X-linked brain malformation phenotype with microcephaly and hypoplasia of the brainstem and cerebellum. *Nat. Genet.* **40**, 1065-1067.

Neale, B. M., Kou, Y., Liu, L., Ma'ayan, A., Samocha, K. E., Sabo, A., Lin, C.-F., Stevens, C., Wang, L.-S., Makarov, V., et al. (2012). Patterns and rates of exonic de novo mutations in autism spectrum disorders. *Nature* **485**, 242-245.

Neely, G. G., Hess, A., Costigan, M., Keene, A. C., Goulas, S., Langeslag, M., Griffin, R. S., Belfer, I., Dai, F., Smith, S. B., et al. (2010). A genome-wide Drosophila screen for heat nociception identifies α2δ3 as an evolutionarily conserved pain gene. *Cell* **143**, 628-638.

Neusch, C., Rozengurt, N., Jacobs, R. E., Lester, H. A. and Kofuji, P. (2001). Kir4.1 potassium channel subunit is crucial for oligodendrocyte development and in vivo myelination. *J. Neurosci.* **21**, 5429-5438.

Noebels, J. L. (2002). Human Epilepsy Can Be Linked to a Defective Calcium Channel. *Epilepsy Curr.* **2**, 95.

Novara, F., Beri, S., Giorda, R., Ortibus, E., Nageshappa, S., Darra, F., Dalla Bernardina, B., Zuffardi, O. and Van Esch, H. (2010). Refining the phenotype associated with MEF2C haploinsufficiency. *Clin. Genet.* **78**, 471-477.

O'Roak, B. J., Deriziotis, P., Lee, C., Vives, L., Schwartz, J. J., Girirajan, S., Karakoc, E., Mackenzie, A. P., Ng, S. B., Baker, C., et al. (2011). Exome sequencing in sporadic autism spectrum disorders identifies severe de novo mutations. *Nat. Genet.* **43**, 585-589.

Development • Supplementary information

O'Roak, B. J., Vives, L., Girirajan, S., Karakoc, E., Krumm, N., Coe, B. P., Levy, R., Ko, A., Lee, C., Smith, J. D., et al. (2012a). Sporadic autism exomes reveal a highly interconnected protein network of de novo mutations. *Nature* **485**, 246-250.

O'Roak, B. J., Vives, L., Fu, W., Egertson, J. D., Stanaway, I. B., Phelps, I. G., Carvill, G., Kumar, A., Lee, C., Ankenman, K., et al. (2012b). Multiplex targeted sequencing identifies recurrently mutated genes in autism spectrum disorders. *Science* **338**, 1619-1622.

Ogden, K. K., Chen, W., Swanger, S. A., McDaniel, M. J., Fan, L. Z., Hu, C., Tankovic, A., Kusumoto, H., Kosobucki, G. J., Schulien, A. J., et al. (2017). Molecular Mechanism of Disease-Associated Mutations in the Pre-M1 Helix of NMDA Receptors and Potential Rescue Pharmacology. *PLoS Genet.* **13**, e1006536.

Ogiwara, I., Miyamoto, H., Morita, N., Atapour, N., Mazaki, E., Inoue, I., Takeuchi, T., Itohara, S., Yanagawa, Y., Obata, K., et al. (2007). Nav1.1 localizes to axons of parvalbumin-positive inhibitory interneurons: a circuit basis for epileptic seizures in mice carrying an Scn1a gene mutation. *J. Neurosci.* **27**, 5903-5914.

Ohtsuki, T., Sakurai, K., Dou, H., Toru, M., Yamakawa-Kobayashi, K. and Arinami, T. (2001). Mutation analysis of the NMDAR2B (GRIN2B) gene in schizophrenia. *Mol. Psychiatry* **6**, 211-216.

Orhan, G., Bock, M., Schepers, D., Ilina, E. I., Reichel, S. N., Löffler, H., Jezutkovic, N., Weckhuysen, S., Mandelstam, S., Suls, A., et al. (2014). Dominant-negative effects of KCNQ2 mutations are associated with epileptic encephalopathy. *Ann. Neurol.* **75**, 382-394.

Ortner, N. J., Kaserer, T., Copeland, J. N. and Striessnig, J. (2020). De novo CACNA1D Ca2+ channelopathies: clinical phenotypes and molecular mechanism. *Pflugers Arch.* **472**, 755-773.

Osaka, H., Ogiwara, I., Mazaki, E., Okamura, N., Yamashita, S., Iai, M., Yamada, M., Kurosawa, K., Iwamoto, H., Yasui-Furukori, N., et al. (2007). Patients with a sodium channel alpha 1 gene mutation show wide phenotypic variation. *Epilepsy Res.* **75**, 46-51.

Panagiotakos, G., Haveles, C., Arjun, A., Petrova, R., Rana, A., Portmann, T., Paşca, S. P., Palmer, T. D. and Dolmetsch, R. E. (2019). Aberrant calcium channel splicing drives defects in cortical differentiation in Timothy syndrome. *Elife* **8**,.

Pardiñas, A. F., Holmans, P., Pocklington, A. J., Escott-Price, V., Ripke, S., Carrera, N., Legge, S. E., Bishop, S., Cameron, D., Hamshere, M. L., et al. (2018). Common schizophrenia alleles are enriched in mutation-intolerant genes and in regions under strong background selection. *Nat. Genet.* **50**, 381-389.

Park, J. and Chung, K. C. (2013). New Perspectives of Dyrk1A Role in Neurogenesis and Neuropathologic Features of Down Syndrome. *Exp. Neurobiol.* **22**, 244-248.

Development • Supplementary information

Parrini, E., Marini, C., Mei, D., Galuppi, A., Cellini, E., Pucatti, D., Chiti, L., Rutigliano, D., Bianchini, C., Virdò, S., et al. (2017). Diagnostic Targeted Resequencing in 349 Patients with Drug-Resistant Pediatric Epilepsies Identifies Causative Mutations in 30 Different Genes. *Hum. Mutat.* **38**, 216-225.

Paşca, S. P., Portmann, T., Voineagu, I., Yazawa, M., Shcheglovitov, A., Paşca, A. M., Cord, B., Palmer, T. D., Chikahisa, S., Nishino, S., et al. (2011). Using iPSC-derived neurons to uncover cellular phenotypes associated with Timothy syndrome. *Nat. Med.* **17**, 1657-1662.

Peng, J., Zhou, Y. and Wang, K. (2021). Multiplex gene and phenotype network to characterize shared genetic pathways of epilepsy and autism. *Sci. Rep.* **11**, 952.

Perez-Reyes, E. (2006). Molecular characterization of T-type calcium channels. *Cell Calcium* **40**, 89-96.

Petrij, F., Giles, R. H., Dauwerse, H. G., Saris, J. J., Hennekam, R. C., Masuno, M., Tommerup, N., van Ommen, G. J., Goodman, R. H. and Peters, D. J. (1995). Rubinstein-Taybi syndrome caused by mutations in the transcriptional co-activator CBP. *Nature* **376**, 348-351.

Pinggera, A., Lieb, A., Benedetti, B., Lampert, M., Monteleone, S., Liedl, K. R., Tuluc, P. and Striessnig, J. (2015). CACNA1D de novo mutations in autism spectrum disorders activate Cav1.3 L-type calcium channels. *Biol. Psychiatry* **77**, 816-822.

Pinggera, A., Mackenroth, L., Rump, A., Schallner, J., Beleggia, F., Wollnik, B. and Striessnig, J. (2017). New gain-of-function mutation shows CACNA1D as recurrently mutated gene in autism spectrum disorders and epilepsy. *Hum. Mol. Genet.* **26**, 2923-2932.

Pinggera, A., Negro, G., Tuluc, P., Brown, M. J., Lieb, A. and Striessnig, J. (2018). Gating defects of disease-causing de novo mutations in Cav1.3 Ca2+ channels. *Channels* **12**, 388-402.

Pirone, A., Kurt, S., Zuccotti, A., Rüttiger, L., Pilz, P., Brown, D. H., Franz, C., Schweizer, M., Rust, M. B., Rübsamen, R., et al. (2014). α2δ3 is essential for normal structure and function of auditory nerve synapses and is a novel candidate for auditory processing disorders. *J. Neurosci.* **34**, 434-445.

Platzer, J., Engel, J., Schrott-Fischer, A., Stephan, K., Bova, S., Chen, H., Zheng, H. and Striessnig, J. (2000). Congenital deafness and sinoatrial node dysfunction in mice lacking class D L-type Ca2+ channels. *Cell* **102**, 89-97.

Plüger, S., Faulhaber, J., Fürstenau, M., Löhn, M., Waldschütz, R., Gollasch, M., Haller, H., Luft, F. C., Ehmke, H. and Pongs, O. (2000). Mice with disrupted BK channel beta1 subunit gene feature abnormal Ca(2+) spark/STOC coupling and elevated blood pressure. *Circ. Res.* **87**, E53-60.

Development • Supplementary information

Powell, K. L., Cain, S. M., Ng, C., Sirdesai, S., David, L. S., Kyi, M., Garcia, E., Tyson, J. R., Reid, C. A., Bahlo, M., et al. (2009). A Cav3.2 T-type calcium channel point mutation has splice-variant-specific effects on function and segregates with seizure expression in a polygenic rat model of absence epilepsy. *J. Neurosci.* **29**, 371-380.

Prandini, P., Pasquali, A., Malerba, G., Marostica, A., Zusi, C., Xumerle, L., Muglia, P., Da Ros, L., Ratti, E., Trabetti, E., et al. (2012). The association of rs4307059 and rs35678 markers with autism spectrum disorders is replicated in Italian families. *Psychiatr. Genet.* **22**, 177-181.

Prasad, A., Merico, D., Thiruvahindrapuram, B., Wei, J., Lionel, A. C., Sato, D., Rickaby, J., Lu, C., Szatmari, P., Roberts, W., et al. (2012). A discovery resource of rare copy number variations in individuals with autism spectrum disorder. *G3* **2**, 1665-1685.

Purcell, S. M., Moran, J. L., Fromer, M., Ruderfer, D., Solovieff, N., Roussos, P., O'Dushlaine, C., Chambert, K., Bergen, S. E., Kähler, A., et al. (2014). A polygenic burden of rare disruptive mutations in schizophrenia. *Nature* **506**, 185-190.

Rees, E., Han, J., Morgan, J., Carrera, N., Escott-Price, V., Pocklington, A. J., Duffield, M., Hall, L. S., Legge, S. E., Pardiñas, A. F., et al. (2020). De novo mutations identified by exome sequencing implicate rare missense variants in SLC6A1 in schizophrenia. *Nat. Neurosci.* **23**, 179-184.

Riazuddin, S., Belyantseva, I. A., Giese, A. P. J., Lee, K., Indzhykulian, A. A., Nandamuri, S. P., Yousaf, R., Sinha, G. P., Lee, S., Terrell, D., et al. (2012). Alterations of the CIB2 calcium- and integrin-binding protein cause Usher syndrome type 1J and nonsyndromic deafness DFNB48. *Nat. Genet.* **44**, 1265-1271.

Ripke, S., O'Dushlaine, C., Chambert, K., Moran, J. L., Kähler, A. K., Akterin, S., Bergen, S. E., Collins, A. L., Crowley, J. J., Fromer, M., et al. (2013). Genome-wide association analysis identifies 13 new risk loci for schizophrenia. *Nat. Genet.* **45**, 1150-1159.

Roelfsema, J. H., White, S. J., Ariyürek, Y., Bartholdi, D., Niedrist, D., Papadia, F., Bacino, C. A., den Dunnen, J. T., van Ommen, G.-J. B., Breuning, M. H., et al. (2005). Genetic heterogeneity in Rubinstein-Taybi syndrome: mutations in both the CBP and EP300 genes cause disease. *Am. J. Hum. Genet.* **76**, 572-580.

Rossignol, E., Kruglikov, I., van den Maagdenberg, A. M. J. M., Rudy, B. and Fishell, G. (2013). CaV 2.1 ablation in cortical interneurons selectively impairs fast-spiking basket cells and causes generalized seizures. *Ann. Neurol.* **74**, 209-222.

Rots, D., Chater-Diehl, E., Dingemans, A. J. M., Goodman, S. J., Siu, M. T., Cytrynbaum, C., Choufani, S., Hoang, N., Walker, S., Awamleh, Z., et al. (2021). Truncating SRCAP variants outside the Floating-Harbor syndrome locus cause a distinct neurodevelopmental disorder with a specific DNA methylation signature. *Am. J. Hum. Genet.* **108**, 1053-1068.

Ruaud, L., Mignot, C., Guët, A., Ohl, C., Nava, C., Héron, D., Keren, B., Depienne, C., Benoit, V., Maystadt, I., et al. (2015). DYRK1A mutations in two unrelated patients. *Eur. J. Med. Genet.* **58**, 168-174.

Rüttiger, L., Sausbier, M., Zimmermann, U., Winter, H., Braig, C., Engel, J., Knirsch, M., Arntz, C., Langer, P., Hirt, B., et al. (2004). Deletion of the Ca2+-activated potassium (BK) alpha-subunit but not the BKbeta1-subunit leads to progressive hearing loss. *Proc. Natl. Acad. Sci. U. S. A.* **101**, 12922-12927.

Saitsu, H., Akita, T., Tohyama, J., Goldberg-Stern, H., Kobayashi, Y., Cohen, R., Kato, M., Ohba, C., Miyatake, S., Tsurusaki, Y., et al. (2015). De novo KCNB1 mutations in infantile epilepsy inhibit repetitive neuronal firing. *Sci. Rep.* **5**, 15199.

Sakimura, K., Kutsuwada, T., Ito, I., Manabe, T., Takayama, C., Kushiya, E., Yagi, T., Aizawa, S., Inoue, Y. and Sugiyama, H. (1995). Reduced hippocampal LTP and spatial learning in mice lacking NMDA receptor epsilon 1 subunit. *Nature* **373**, 151-155.

Salpietro, V., Dixon, C. L., Guo, H., Bello, O. D., Vandrovcova, J., Efthymiou, S., Maroofian, R., Heimer, G., Burglen, L., Valence, S., et al. (2019). AMPA receptor GluA2 subunit defects are a cause of neurodevelopmental disorders. *Nat. Commun.* **10**, 3094.

Sanders, S. J., Murtha, M. T., Gupta, A. R., Murdoch, J. D., Raubeson, M. J., Willsey, A. J., Ercan-Sencicek, A. G., DiLullo, N. M., Parikshak, N. N., Stein, J. L., et al. (2012). De novo mutations revealed by whole-exome sequencing are strongly associated with autism. *Nature* **485**, 237-241.

Sanders, S. J., He, X., Willsey, A. J., Ercan-Sencicek, A. G., Samocha, K. E., Cicek, A. E., Murtha, M. T., Bal, V. H., Bishop, S. L., Dong, S., et al. (2015). Insights into Autism Spectrum Disorder Genomic Architecture and Biology from 71 Risk Loci. *Neuron* **87**, 1215-1233.

Sanders, S. J., Campbell, A. J., Cottrell, J. R., Moller, R. S., Wagner, F. F., Auldridge, A. L., Bernier, R. A., Catterall, W. A., Chung, W. K., Empfield, J. R., et al. (2018). Progress in understanding and treating SCN2A-mediated disorders. *Trends Neurosci.* **41**, 442-456.

Sands, T. T., Miceli, F., Lesca, G., Beck, A. E., Sadleir, L. G., Arrington, D. K., Schönewolf-Greulich, B., Moutton, S., Lauritano, A., Nappi, P., et al. (2019). Autism and developmental disability caused by KCNQ3 gain-of-function variants. *Ann. Neurol.* **86**, 181-192.

Satterstrom, F. K., Walters, R. K., Singh, T., Wigdor, E. M., Lescai, F., Demontis, D., Kosmicki, J. A., Grove, J., Stevens, C., Bybjerg-Grauholm, J., et al. (2019). Autism spectrum disorder and attention deficit hyperactivity disorder have a similar burden of rare protein-truncating variants. *Nat. Neurosci.* **22**, 1961-1965.

Sun, Y., Paşca, S. P., Portmann, T., Goold, C., Worringer, K. A., Guan, W., Chan, K. C., Gai, H., Vogt, D., Chen, Y.-J. J., et al. (2016). A deleterious Nav1.1 mutation selectively impairs telencephalic inhibitory neurons derived from Dravet Syndrome patients. *Elife* **5**, e13073.

Satterstrom, F. K., Kosmicki, J. A., Wang, J., Breen, M. S., De Rubeis, S., An, J.-Y., Peng, M., Collins, R., Grove, J., Klei, L., et al. (2020). Large-Scale Exome Sequencing Study Implicates Both Developmental and Functional Changes in the Neurobiology of Autism. *Cell* **180**, 568-584.e23.

Saunders, J. A., Tatard-Leitman, V. M., Suh, J., Billingslea, E. N., Roberts, T. P. and Siegel, S. J. (2013). Knockout of NMDA receptors in parvalbumin interneurons recreates autism-like phenotypes. *Autism Res.* **6**, 69-77.

Sausbier, M., Hu, H., Arntz, C., Feil, S., Kamm, S., Adelsberger, H., Sausbier, U., Sailer, C. A., Feil, R., Hofmann, F., et al. (2004). Cerebellar ataxia and Purkinje cell dysfunction caused by Ca^{2+}-activated K^{+} channel deficiency. *Proceedings of the National Academy of Sciences* **101**, 9474-9478.

Sceniak, M. P., Fedder, K. N., Wang, Q., Droubi, S., Babcock, K., Patwardhan, S., Wright-Zornes, J., Pham, L. and Sabo, S. L. (2019). An autism-associated mutation in GluN2B prevents NMDA receptor trafficking and interferes with dendrite growth. *J. Cell Sci.* **132**,.

Schirmer, L., Möbius, W., Zhao, C., Cruz-Herranz, A., Ben Haim, L., Cordano, C., Shiow, L. R., Kelley, K. W., Sadowski, B., Timmons, G., et al. (2018). Oligodendrocyte-encoded Kir4.1 function is required for axonal integrity. *Elife* **7**,.

Schizophrenia Psychiatric Genome-Wide Association Study (GWAS) Consortium (2011). Genome-wide association study identifies five new schizophrenia loci. *Nat. Genet.* **43**, 969-976.

Schizophrenia Working Group of the Psychiatric Genomics Consortium (2014). Biological insights from 108 schizophrenia-associated genetic loci. *Nature* **511**, 421-427.

Scholl, U. I., Goh, G., Stölting, G., de Oliveira, R. C., Choi, M., Overton, J. D., Fonseca, A. L., Korah, R., Starker, L. F., Kunstman, J. W., et al. (2013). Somatic and germline CACNA1D calcium channel mutations in aldosterone-producing adenomas and primary aldosteronism. *Nat. Genet.* **45**, 1050-1054.

Schoof, M., Launspach, M., Holdhof, D., Nguyen, L., Engel, V., Filser, S., Peters, F., Immenschuh, J., Hellwig, M., Niesen, J., et al. (2019). The transcriptional coactivator and histone acetyltransferase CBP regulates neural precursor cell development and migration. *Acta Neuropathol Commun* **7**, 199.

Sebat, J., Lakshmi, B., Malhotra, D., Troge, J., Lese-Martin, C., Walsh, T., Yamrom, B., Yoon, S., Krasnitz, A., Kendall, J., et al. (2007). Strong association of de novo copy number mutations with autism. *Science* **316**, 445-449.

Serraz, B., Grand, T. and Paoletti, P. (2016). Altered zinc sensitivity of NMDA receptors harboring clinically-relevant mutations. *Neuropharmacology* **109**, 196-204.

Shaikh, M. N., Gutierrez-Aviño, F., Colonques, J., Ceron, J., Hämmerle, B. and Tejedor, F. J. (2016). Minibrain drives the Dacapo-dependent cell cycle exit of neurons in the Drosophila brain by promoting asense and prospero expression. *Development* **143**, 3195-3205.

Shinawi, M., Schaaf, C. P., Bhatt, S. S., Xia, Z., Patel, A., Cheung, S. W., Lanpher, B., Nagl, S., Herding, H. S., Nevinny-Stickel, C., et al. (2009). A small recurrent deletion within 15q13.3 is associated with a range of neurodevelopmental phenotypes. *Nat. Genet.* **41**, 1269-1271.

Sicca, F., Imbrici, P., D'Adamo, M. C., Moro, F., Bonatti, F., Brovedani, P., Grottesi, A., Guerrini, R., Masi, G., Santorelli, F. M., et al. (2011). Autism with seizures and intellectual disability: possible causative role of gain-of-function of the inwardly-rectifying K+ channel Kir4.1. *Neurobiol. Dis.* **43**, 239-247.

Singh, N. A., Charlier, C., Stauffer, D., DuPont, B. R., Leach, R. J., Melis, R., Ronen, G. M., Bjerre, I., Quattlebaum, T., Murphy, J. V., et al. (1998). A novel potassium channel gene, KCNQ2, is mutated in an inherited epilepsy of newborns. *Nat. Genet.* **18**, 25-29.

Singh, T., Poterba, T., Curtis, D., Akil, H., Al Eissa, M., Barchas, J. D., Bass, N., Bigdeli, T. B., Breen, G., Bromet, E. J., et al. (2022). Rare coding variants in ten genes confer substantial risk for schizophrenia. *Nature* **604**, 509-516.

Sinkus, M. L., Wamboldt, M. Z., Barton, A., Fingerlin, T. E., Laudenslager, M. L. and Leonard, S. (2011). The α7 nicotinic acetylcholine receptor and the acute stress response: maternal genotype determines offspring phenotype. *Physiol. Behav.* **104**, 321-326.

Smits, J. J., Oostrik, J., Beynon, A. J., Kant, S. G., de Koning Gans, P. A. M., Rotteveel, L. J. C., Klein Wassink-Ruiter, J. S., Free, R. H., Maas, S. M., van de Kamp, J., et al. (2019). De novo and inherited loss-of-function variants of ATP2B2 are associated with rapidly progressive hearing impairment. *Hum. Genet.* **138**, 61-72.

Soh, H., Pant, R., LoTurco, J. J. and Tzingounis, A. V. (2014). Conditional deletions of epilepsy-associated KCNQ2 and KCNQ3 channels from cerebral cortex cause differential effects on neuronal excitability. *J. Neurosci.* **34**, 5311-5321.

Soler-Alfonso, C., Carvalho, C. M. B., Ge, J., Roney, E. K., Bader, P. I., Kolodziejska, K. E., Miller, R. M., Lupski, J. R., Stankiewicz, P., Cheung, S. W., et al. (2014). CHRNA7 triplication associated with cognitive impairment and neuropsychiatric phenotypes in a three-generation pedigree. *Eur. J. Hum. Genet.* **22**, 1071-1076.

Speca, D. J., Ogata, G., Mandikian, D., Bishop, H. I., Wiler, S. W., Eum, K., Wenzel, H. J., Doisy, E. T., Matt, L., Campi, K. L., et al. (2014). Deletion of the Kv2.1 delayed rectifier potassium channel leads to neuronal and behavioral hyperexcitability. *Genes Brain Behav.* **13**, 394-408.

Splawski, I., Timothy, K. W., Sharpe, L. M., Decher, N., Kumar, P., Bloise, R., Napolitano, C., Schwartz, P. J., Joseph, R. M., Condouris, K., et al. (2004). Ca(V)1.2 calcium channel dysfunction causes a multisystem disorder including arrhythmia and autism. *Cell* **119**, 19-31.

Splawski, I., Yoo, D. S., Stotz, S. C., Cherry, A., Clapham, D. E. and Keating, M. T. (2006). CACNA1H mutations in autism spectrum disorders. *J. Biol. Chem.* **281**, 22085-22091.

Spratt, P. W. E., Ben-Shalom, R., Keeshen, C. M., Burke, K. J., Jr, Clarkson, R. L., Sanders, S. J. and Bender, K. J. (2019). The autism-associated gene Scn2a contributes to dendritic excitability and synaptic function in the prefrontal cortex. *Neuron* **103**, 673-685.e5.

Spratt, P. W. E., Alexander, R. P. D., Ben-Shalom, R., Sahagun, A., Kyoung, H., Keeshen, C. M., Sanders, S. J. and Bender, K. J. (2021). Paradoxical hyperexcitability from NaV1.2 sodium channel loss in neocortical pyramidal cells. *Cell Rep.* **36**,.

Srivastava, S., Cohen, J. S., Vernon, H., Barañano, K., McClellan, R., Jamal, L., Naidu, S. and Fatemi, A. (2014a). Clinical whole exome sequencing in child neurology practice. *Ann. Neurol.* **76**, 473-483.

Srivastava, S., Cohen, J., Pevsner, J., Aradhya, S., McKnight, D., Butler, E., Johnston, M. and Fatemi, A. (2014b). A novel variant in GABRB2 associated with intellectual disability and epilepsy. *Am. J. Med. Genet. A* **164A**, 2914-2921.

Stam, A. H., Luijckx, G.-J., Poll-Thé, B. T., Ginjaar, I. B., Frants, R. R., Haan, J., Ferrari, M. D., Terwindt, G. M. and van den Maagdenberg, A. M. J. M. (2009). Early seizures and cerebral oedema after trivial head trauma associated with the CACNA1A S218L mutation. *J. Neurol. Neurosurg. Psychiatry* **80**, 1125-1129.

Stephens, S. H., Logel, J., Barton, A., Franks, A., Schultz, J., Short, M., Dickenson, J., James, B., Fingerlin, T. E., Wagner, B., et al. (2009). Association of the 5’-upstream regulatory region of the alpha7 nicotinic acetylcholine receptor subunit gene (CHRNA7) with schizophrenia. *Schizophr. Res.* **109**, 102-112.

Stessman, H. A. F., Xiong, B., Coe, B. P., Wang, T., Hoekzema, K., Fenckova, M., Kvarnung, M., Gerdts, J., Trinh, S., Cosemans, N., et al. (2017). Targeted sequencing identifies 91 neurodevelopmental-disorder risk genes with autism and developmental-disability biases. *Nat. Genet.* **49**, 515-526.

Strehlow, V., Heyne, H. O., Vlaskamp, D. R. M., Marwick, K. F. M., Rudolf, G., de Bellescize, J., Biskup, S., Brilstra, E. H., Brouwer, O. F., Callenbach, P. M. C., et al. (2019). GRIN2A-related disorders: genotype and functional consequence predict phenotype. *Brain* **142**, 80-92.

Sundaram, S. K., Chugani, H. T., Tiwari, V. N. and Huq, A. H. M. M. (2013). SCN2A mutation is associated with infantile spasms and bitemporal glucose hypometabolism. *Pediatr. Neurol.* **49**, 46-49.

Swanger, S. A., Chen, W., Wells, G., Burger, P. B., Tankovic, A., Bhattacharya, S., Strong, K. L., Hu, C., Kusumoto, H., Zhang, J., et al. (2016). Mechanistic Insight into NMDA Receptor Dysregulation by Rare Variants in the GluN2A and GluN2B Agonist Binding Domains. *Am. J. Hum. Genet.* **99**, 1261-1280.

Tabarki, B., AlMajhad, N., AlHashem, A., Shaheen, R. and Alkuraya, F. S. (2016). Homozygous KCNMA1 mutation as a cause of cerebellar atrophy, developmental delay and seizures. *Hum. Genet.* **135**, 1295-1298.

Takata, A., Ionita-Laza, I., Gogos, J. A., Xu, B. and Karayiorgou, M. (2016). De Novo Synonymous Mutations in Regulatory Elements Contribute to the Genetic Etiology of Autism and Schizophrenia. *Neuron* **89**, 940-947.

Takata, A., Miyake, N., Tsurusaki, Y., Fukai, R., Miyatake, S., Koshimizu, E., Kushima, I., Okada, T., Morikawa, M., Uno, Y., et al. (2018). Integrative Analyses of De Novo Mutations Provide Deeper Biological Insights into Autism Spectrum Disorder. *Cell Rep.* **22**, 734-747.

Tanaka, M., Olsen, R. W., Medina, M. T., Schwartz, E., Alonso, M. E., Duron, R. M., Castro-Ortega, R., Martinez-Juarez, I. E., Pascual-Castroviejo, I., Machado-Salas, J., et al. (2008). Hyperglycosylation and reduced GABA currents of mutated GABRB3 polypeptide in remitting childhood absence epilepsy. *Am. J. Hum. Genet.* **82**, 1249-1261.

Tanaka, M., DeLorey, T. M., Delgado-Escueta, A. and Olsen, R. W. (2012). GABRB3, Epilepsy, and Neurodevelopment. In *Jasper's Basic Mechanisms of the Epilepsies* (ed. Noebels, J. L.), Avoli, M.), Rogawski, M. A.), Olsen, R. W.), and Delgado-Escueta, A. V.), Bethesda (MD): National Center for Biotechnology Information (US).

Tang, Y. P., Shimizu, E., Dube, G. R., Rampon, C., Kerchner, G. A., Zhuo, M., Liu, G. and Tsien, J. Z. (1999). Genetic enhancement of learning and memory in mice. *Nature* **401**, 63-69.

Tang, Z. Z., Zheng, S., Nikolic, J. and Black, D. L. (2009). Developmental control of CaV1.2 L-type calcium channel splicing by Fox proteins. *Mol. Cell. Biol.* **29**, 4757-4765.

Tao, J., Hildebrand, M. E., Liao, P., Liang, M. C., Tan, G., Li, S., Snutch, T. P. and Soong, T. W. (2008). Activation of corticotropin-releasing factor receptor 1 selectively inhibits CaV3.2 T-type calcium channels. *Mol. Pharmacol.* **73**, 1596-1609.

Tarabeux, J., Kebir, O., Gauthier, J., Hamdan, F. F., Xiong, L., Piton, A., Spiegelman, D., Henrion, É., Millet, B., S2D team, et al. (2011). Rare mutations in N-methyl-D-aspartate glutamate receptors in autism spectrum disorders and schizophrenia. *Transl. Psychiatry* **1**, e55.

Tavassoli, T., Kolevzon, A., Wang, A. T., Curchack-Lichtin, J., Halpern, D., Schwartz, L., Soffes, S., Bush, L., Grodberg, D., Cai, G., et al. (2014). De novo SCN2A splice site mutation in a boy with Autism spectrum disorder. *BMC Med. Genet.* **15**, 35.

Tejedor, F., Zhu, X. R., Kaltenbach, E., Ackermann, A., Baumann, A., Canal, I., Heisenberg, M., Fischbach, K. F. and Pongs, O. (1995). minibrain: a new protein kinase family involved in postembryonic neurogenesis in Drosophila. *Neuron* **14**, 287-301.

Thiffault, I., Speca, D. J., Austin, D. C., Cobb, M. M., Eum, K. S., Safina, N. P., Grote, L., Farrow, E. G., Miller, N., Soden, S., et al. (2015). A novel epileptic encephalopathy mutation in KCNB1 disrupts Kv2.1 ion selectivity, expression, and localization. *J. Gen. Physiol.* **146**, 399-410.

Todorov, B., van de Ven, R. C. G., Kaja, S., Broos, L. A. M., Verbeek, S. J., Plomp, J. J., Ferrari, M. D., Frants, R. R. and van den Maagdenberg, A. M. J. M. (2006). Conditional inactivation of the Cacna1a gene in transgenic mice. *Genesis* **44**, 589-594.

Torkamani, A., Bersell, K., Jorge, B. S., Bjork, R. L., Jr, Friedman, J. R., Bloss, C. S., Cohen, J., Gupta, S., Naidu, S., Vanoye, C. G., et al. (2014). De novo KCNB1 mutations in epileptic encephalopathy. *Ann. Neurol.* **76**, 529-540.

Trudler, D., Ghatak, S., Parker, J., Talantova, M., Grabauskas, T., Noveral, S. M., Teranaka, M., Luevanos, M., Dolatabadi, N., Bakker, C., et al. (2020). Aberrant gliogenesis and excitation in MEF2C autism patient hiPSC-neurons and cerebral organoids. *bioRxiv* 2020.11.19.387639.

Urak, L., Feucht, M., Fathi, N., Hornik, K. and Fuchs, K. (2006). A GABRB3 promoter haplotype associated with childhood absence epilepsy impairs transcriptional activity. *Hum. Mol. Genet.* **15**, 2533-2541.

Valor, L. M., Pulopulos, M. M., Jimenez-Minchan, M., Olivares, R., Lutz, B. and Barco, A. (2011). Ablation of CBP in forebrain principal neurons causes modest memory and transcriptional defects and a dramatic reduction of histone acetylation but does not affect cell viability. *J. Neurosci.* **31**, 1652-1663.

van Bon, B. W. M., Hoischen, A., Hehir-Kwa, J., de Brouwer, A. P. M., Ruivenkamp, C., Gijsbers, A. C. J., Marcelis, C. L., de Leeuw, N., Veltman, J. A., Brunner, H. G., et al. (2011). Intragenic deletion in DYRK1A leads to mental retardation and primary microcephaly. *Clin. Genet.* **79**, 296-299.

van Bon, B. W. M., Coe, B. P., Bernier, R., Green, C., Gerdts, J., Witherspoon, K., Kleefstra, T., Willemsen, M. H., Kumar, R., Bosco, P., et al. (2016). Disruptive de novo mutations of DYRK1A lead to a syndromic form of autism and ID. *Mol. Psychiatry* **21**, 126-132.

van den Maagdenberg, A. M. J. M., Pietrobon, D., Pizzorusso, T., Kaja, S., Broos, L. A. M., Cesetti, T., van de Ven, R. C. G., Tottene, A., van der Kaa, J., Plomp, J. J., et al. (2004). A Cacna1a knockin migraine mouse model with increased susceptibility to cortical spreading depression. *Neuron* **41**, 701-710.

Veeramah, K. R., O'Brien, J. E., Meisler, M. H., Cheng, X., Dib-Hajj, S. D., Waxman, S. G., Talwar, D., Girirajan, S., Eichler, E. E., Restifo, L. L., et al. (2012). De novo pathogenic SCN8A mutation identified by whole-genome sequencing of a family quartet affected by infantile epileptic encephalopathy and SUDEP. *Am. J. Hum. Genet.* **90**, 502-510.

Vieira, M. M., Jeong, J. and Roche, K. W. (2021). The role of NMDA receptor and neuroligin rare variants in synaptic dysfunction underlying neurodevelopmental disorders. *Curr. Opin. Neurobiol.* **69**, 93-104.

Vitko, I., Chen, Y., Arias, J. M., Shen, Y., Wu, X.-R. and Perez-Reyes, E. (2005). Functional characterization and neuronal modeling of the effects of childhood absence epilepsy variants of CACNA1H, a T-type calcium channel. *J. Neurosci.* **25**, 4844-4855.

Vitko, I., Bidaud, I., Arias, J. M., Mezghrani, A., Lory, P. and Perez-Reyes, E. (2007). The I-II loop controls plasma membrane expression and gating of Ca(v)3.2 T-type Ca2+ channels: a paradigm for childhood absence epilepsy mutations. *J. Neurosci.* **27**, 322-330.

Wagnon, J. L. and Meisler, M. H. (2015). Recurrent and Non-Recurrent Mutations of SCN8A in Epileptic Encephalopathy. *Front. Neurol.* **6**, 104.

Wagnon, J. L., Korn, M. J., Parent, R., Tarpey, T. A., Jones, J. M., Hammer, M. F., Murphy, G. G., Parent, J. M. and Meisler, M. H. (2015). Convulsive seizures and SUDEP in a mouse model of SCN8A epileptic encephalopathy. *Hum. Mol. Genet.* **24**, 506-515.

Wamsley, B., Jaglin, X. H., Favuzzi, E., Quattrocolo, G., Nigro, M. J., Yusuf, N., Khodadadi-Jamayran, A., Rudy, B. and Fishell, G. (2018). Rbfox1 Mediates Cell-type-Specific Splicing in Cortical Interneurons. *Neuron* **100**, 846-859.e7.

Wang, H.-X. and Gao, W.-J. (2009). Cell type-specific development of NMDA receptors in the interneurons of rat prefrontal cortex. *Neuropsychopharmacology* **34**, 2028-2040.

Wang, W., Mei, X.-P., Wei, Y.-Y., Zhang, M.-M., Zhang, T., Wang, W., Xu, L.-X., Wu, S.-X. and Li, Y.-Q. (2011). Neuronal NR2B-containing NMDA receptor mediates spinal astrocytic c-Jun N-terminal kinase activation in a rat model of neuropathic pain. *Brain Behav. Immun.* **25**, 1355-1366.

Wang, Y., Li, J., Yao, X., Li, W., Du, H., Tang, M., Xiong, W., Chai, R. and Xu, Z. (2017). Loss of CIB2 Causes Profound Hearing Loss and Abolishes Mechanoelectrical Transduction in Mice. *Front. Mol. Neurosci.* **10**, 401.

Wang, J., Poliquin, S., Mermer, F., Eissman, J., Delpire, E., Wang, J., Shen, W., Cai, K., Li, B.-M., Li, Z.-Y., et al. (2020). Endoplasmic reticulum retention and degradation of a mutation in SLC6A1 associated with epilepsy and autism. *Mol. Brain* **13**, 76.

Weiss, L. A., Escayg, A., Kearney, J. A., Trudeau, M., MacDonald, B. T., Mori, M., Reichert, J., Buxbaum, J. D. and Meisler, M. H. (2003). Sodium channels SCN1A, SCN2A and SCN3A in familial autism. *Mol. Psychiatry* **8**, 186-194.

Wincent, J., Luthman, A., van Belzen, M., van der Lans, C., Albert, J., Nordgren, A. and Anderlid, B.-M. (2016). CREBBP and EP300 mutational spectrum and clinical presentations in a cohort of Swedish patients with Rubinstein-Taybi syndrome. *Mol Genet Genomic Med* **4**, 39-45.

Xu, X.-X., Liu, X.-R., Fan, C.-Y., Lai, J.-X., Shi, Y.-W., Yang, W., Su, T., Xu, J.-Y., Luo, J.-H. and Liao, W.-P. (2018). Functional Investigation of a GRIN2A Variant Associated with Rolandic Epilepsy. *Neurosci. Bull.* **34**, 237-246.

Yabut, O., Domogauer, J. and D'Arcangelo, G. (2010). Dyrk1A overexpression inhibits proliferation and induces premature neuronal differentiation of neural progenitor cells. *J. Neurosci.* **30**, 4004-4014.

Yang, W., Liu, J., Zheng, F., Jia, M., Zhao, L., Lu, T., Ruan, Y., Zhang, J., Yue, W., Zhang, D., et al. (2013). The evidence for association of ATP2B2 polymorphisms with autism in Chinese Han population. *PLoS One* **8**, e61021.

Yang, S., Guo, X., Dong, X., Han, Y., Gao, L., Su, Y., Dai, W. and Zhang, X. (2017). GABAA receptor subunit gene polymorphisms predict symptom-based and developmental deficits in Chinese Han children and adolescents with autistic spectrum disorders. *Sci. Rep.* **7**, 3290.

Yang, Y., Xiangwei, W., Zhang, X., Xiao, J., Chen, J., Yang, X., Jia, T., Yang, Z., Jiang, Y. and Zhang, Y. (2020). Phenotypic spectrum of patients with GABRB2 variants: from mild febrile seizures to severe epileptic encephalopathy. *Dev. Med. Child Neurol.* **62**, 1213-1220.

Yeung, R. K., Xiang, Z.-H., Tsang, S.-Y., Li, R., Ho, T. Y. C., Li, Q., Hui, C.-K., Sham, P.-C., Qiao, M.-Q. and Xue, H. (2018). Gabrb2-knockout mice displayed schizophrenia-like and comorbid phenotypes with interneuron–astrocyte–microglia dysregulation. *Transl. Psychiatry* **8**, 1-14.

Yin, J., Chen, W., Yang, H., Xue, M. and Schaaf, C. P. (2017). Chrna7 deficient mice manifest no consistent neuropsychiatric and behavioral phenotypes. *Sci. Rep.* **7**, 1-10.

Yoo, H. J., Cho, I. H., Park, M., Yang, S. Y. and Kim, S. A. (2012). Family based association of GRIN2A and GRIN2B with Korean autism spectrum disorders. *Neurosci. Lett.* **512**, 89-93.

Yuan, H., Hansen, K. B., Zhang, J., Pierson, T. M., Markello, T. C., Fajardo, K. V. F., Holloman, C. M., Golas, G., Adams, D. R., Boerkoel, C. F., et al. (2014). Functional analysis of a de novo GRIN2A missense mutation associated with early-onset epileptic encephalopathy. *Nat. Commun.* **5**, 3251.

Development • Supplementary information

Yuen, R. K. C., Thiruvahindrapuram, B., Merico, D., Walker, S., Tammimies, K., Hoang, N., Chrysler, C., Nalpathamkalam, T., Pellecchia, G., Liu, Y., et al. (2015). Whole-genome sequencing of quartet families with autism spectrum disorder. *Nat. Med.* **21**, 185-191.

Zaman, T., Lee, K., Park, C., Paydar, A., Choi, J. H., Cheong, E., Lee, C. J. and Shin, H.-S. (2011). Cav2.3 channels are critical for oscillatory burst discharges in the reticular thalamus and absence epilepsy. *Neuron* **70**, 95-108.

Zhang, Z.-B., Tian, M.-Q., Gao, K., Jiang, Y.-W. and Wu, Y. (2015). De novo KCNMA1 mutations in children with early-onset paroxysmal dyskinesia and developmental delay. *Mov. Disord.* **30**, 1290-1292.

Zhang, J., Kim, E. C., Chen, C., Procko, E., Pant, S., Lam, K., Patel, J., Choi, R., Hong, M., Joshi, D., et al. (2020a). Identifying mutation hotspots reveals pathogenetic mechanisms of KCNQ2 epileptic encephalopathy. *Sci. Rep.* **10**, 1-19.

Zhang, G., Gibson, R. A., McDonald, M., Liang, P., Kang, P. W., Shi, J., Yang, H., Cui, J. and Mikati, M. A. (2020b). A gain-of-function mutation in KCNMA1 causes dystonia spells controlled with stimulant therapy. *Mov. Disord.* **35**, 1868-1873.

Zhang, J., Chen, X., Eaton, M., Wu, J., Ma, Z., Lai, S., Park, A., Ahmad, T. S., Que, Z., Lee, J. H., et al. (2021). Severe deficiency of the voltage-gated sodium channel NaV1.2 elevates neuronal excitability in adult mice. *Cell Rep.* **36**,.

Zhao, W.-W. (2013). Intragenic deletion of RBFOX1 associated with neurodevelopmental/neuropsychiatric disorders and possibly other clinical presentations. *Mol. Cytogenet.* **6**, 26.

Zhao, Q., Che, R., Zhang, Z., Wang, P., Li, J., Li, Y., Huang, K., Tang, W., Feng, G., Lindpaintner, K., et al. (2011). Positive association between GRIN2B gene and bipolar disorder in the Chinese Han Population. *Psychiatry Res.* **185**, 290-292.

Zheng, F., Kasper, L. H., Bedford, D. C., Lerach, S., Teubner, B. J. W. and Brindle, P. K. (2016). Mutation of the CH1 Domain in the Histone Acetyltransferase CREBBP Results in Autism-Relevant Behaviors in Mice. *PLoS One* **11**, e0146366.

Zhou, J., Du, W., Zhou, K., Tai, Y., Yao, H., Jia, Y., Ding, Y. and Wang, Y. (2008). Critical role of TRPC6 channels in the formation of excitatory synapses. *Nat. Neurosci.* **11**, 741-743.

Zou, D., Wang, L., Liao, J., Xiao, H., Duan, J., Zhang, T., Li, J., Yin, Z., Zhou, J., Yan, H., et al. (2021). Genome sequencing of 320 Chinese children with epilepsy: a clinical and molecular study. *Brain* **144**, 3623-3634.

Development • Supplementary information
